# Flavour Volatiles of Fermented Vegetable and Fruit Substrates: A Review

**DOI:** 10.3390/molecules28073236

**Published:** 2023-04-04

**Authors:** Sarathadevi Rajendran, Patrick Silcock, Phil Bremer

**Affiliations:** 1 Department of Food Science, University of Otago, Dunedin 9054, New Zealand; 2 Department of Agricultural Chemistry, Faculty of Agriculture, University of Jaffna, Kilinochchi 42400, Sri Lanka

**Keywords:** volatile organic compounds (VOCs), plant-based substrates, lactic acid bacteria (LAB)

## Abstract

Health, environmental and ethical concerns have resulted in a dramatic increase in demand for plant-based dairy analogues. While the volatile organic compounds (VOCs) responsible for the characteristic flavours of dairy-based products have been extensively studied, little is known about how to reproduce such flavours using only plant-based substrates. As a first step in their development, this review provides an overview of the VOCs associated with fermented (bacteria and/or fungi/yeast) vegetable and fruit substrates. Following PRISMA guidelines and using two English databases (Web of Science and Scopus), thirty-five suitable research papers were identified. The number of fermentation-derived VOCs detected ranged from 32 to 118 (across 30 papers), while 5 papers detected fewer (10 to 25). Bacteria, including lactic acid bacteria (LAB), fungi, and yeast were the micro-organisms used, with LAB being the most commonly reported. Ten studies used a single species, 21 studies used a single type (bacteria, fungi or yeast) of micro-organisms and four studies used mixed fermentation. The nature of the fermentation-derived VOCs detected (alcohols, aldehydes, esters, ketones, acids, terpenes and norisoprenoids, phenols, furans, sulphur compounds, alkenes, alkanes, and benzene derivatives) was dependent on the composition of the vegetable/fruit matrix, the micro-organisms involved, and the fermentation conditions.

## 1. Introduction

The demand for plant-based foods is rapidly increasing. Consumers’ reasons for eating more plant-based foods are varied but mainly revolve around a desire to enhance their health by reducing their risk of diseases such as heart disease, cancer, and type 2 diabetes [1], concerns around the sustainability of meat and dairy-based food production systems [2], and/or a desire to move away from the exploitation of animals [3]. Accordingly, the global market for plant-based foods is predicted to reach USD 480.43 billion by 2024 with a predicted CAGR (compound annual growth rate) of 13.82% between 2019 and 2024 [4]. Despite plant-based diets becoming increasingly popular, many consumers still prefer to prepare their meals from foods that are familiar to them in terms of their flavour, appearance, preparation, and cooking method [3]. In response to these needs, the number and range of dairy analogues available on the market are rapidly increasing [5]. While currently available dairy analogues generally have a realistic texture and appearance, their flavour is often uncharacteristic of the dairy products they are attempting to mimic.

Flavour is an important sensory attribute of food, and it is a multimodal sensory experience with the major modality contributors being taste and smell (retronasal). A wide range of non-volatile organic compounds (NVOCs) along with multiple odour-active volatile organic compounds (VOCs) contribute to the taste, aroma, and overall perceived flavour of a food [6,7]. VOCs are typically small compounds (up to C_20_), which have a low molecular weight (<300–400 Da), and a relatively high vapour pressure at ambient temperature. Such compounds can be easily transferred into the vapour phase, which means depending on their concentration, they have the potential to induce an odour sensation [8,9].

Fat, protein (casein, whey/amino acids), sugars (lactose), and citrate are the key components of raw/processed milk, and they are responsible for a wide range of flavour VOCs. The sensory perception of fresh milk is largely determined by a pleasant mouthfeel owing to the physical composition of milk, a slight sweet/salty taste derived from lactose and milk salts, and a delicate aroma due to the VOCs present. In addition, lactic acid bacteria (LAB), when present in fermented dairy products, produce flavour VOCs from sugars or citrate (acetoin, diacetyl, propionic acid, and acetaldehyde), amino acids (benzaldehyde, 3-methylbutanal/3-methyl butanol, methional, methanethiol, phenyl acetic acid, dimethyl sulphide, dimethyl disulphide, and dimethyl trisulphide), and lipids (butanoic acid, butanone, octene-3-ol, hexanal, and δ-decalactone) [10,11].

As many consumers wish to avoid dairy-derived products completely, dairy-derived flavours are generally not acceptable components of dairy analogues. For this reason, the flavours or the substrates needed to generate appropriate flavours need to originate from other sources, with plants owing to diversity, availability and affordability being the most logical choice. However, as dairy flavours contain specific VOCs, as outlined above, producing these flavours and simulating the desired flavour from plant substrates is challenging. In addition, when using plant-based substrates, the flavours of interest may also be associated with other less desirable flavours contained in the plant material matrix, as plants typically contain complex mixtures of substrates [10,12].

The use of micro-organisms to produce desirable flavours from plant-based substrates has gained interest owing to the diversity of the micro-organisms, and the wide range of metabolic reactions they can carry out. Micro-organisms growing on plant-based substrates utilise the available sugars, lipids, and proteins for their energy, synthesis, and growth needs and, in this process, produce a wide range of volatile secondary metabolites through various metabolic pathways [13].

Microbial flavours can therefore be produced in a relatively simple and environmentally friendly/food-grade manner, and such approaches are generally scalable and affordable [14]. The VOCs (composition and concentration) produced by microbial fermentation are dependent on the substrate, type of fermenting micro-organisms (bacteria, LAB, fungi, and yeast), substrate treatments prior to fermentation, and fermentation conditions (aerobic/anaerobic, time, temperature, moisture content, and pH) [9,15].

A better understanding of the substrates available in plants and the metabolic pathways present in micro-organisms will facilitate the use of microbial biosynthesis/fermentation as a means of synthesising VOCs from plant-based substrates that are able to be used as dairy flavours or flavour precursor compounds. As the first step in developing this understanding, the current review presents the range of VOCs reported to be produced during the fermentation of fruit or vegetable substrates, with a focus on flavour VOCs that are known to contribute to dairy-like flavours.

## 2. Research Methods

**Figure 1:**
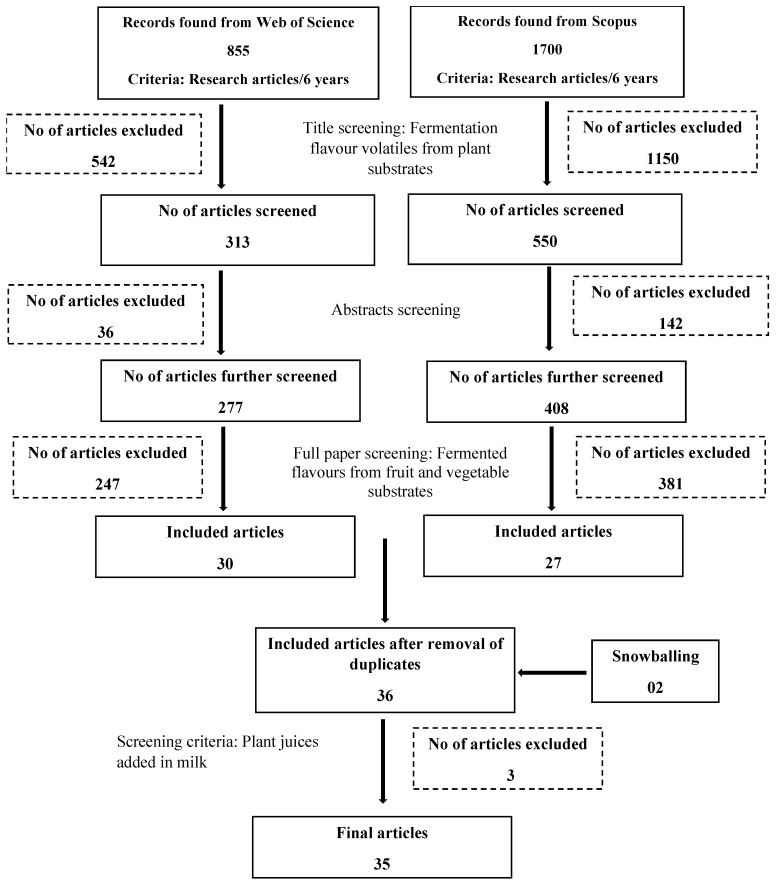
PRISMA flowchart of the review on flavour volatiles of fermented vegetable and fruit substrates.

A review based on PRISMA (Preferred Reporting Items for Systematic Reviews and Meta-Analyses) guidelines [16] was carried out to search for research articles describing the VOCs obtained from the microbial fermentation of plant-based substrates. The goal of the review was to determine the main VOCs reported as being generated from fermented fruit or vegetable substrates, the range of fruit and vegetable substrates used, the different micro-organisms used in previous studies, the fermentation conditions used for VOC production, and the methods used for VOC detection. A search of the electronic databases, Web of Science and Scopus for original research articles written in English over the last six years (from 2017 to April 2022) using the following keywords; “Fermentation flavor volatiles”, “Fermentation flavour volatiles”, “Fermentative aromatic compounds”, “Fermentation flavours vegetable and fruit juices”, “Fermentation flavours vegetable and fruit substrates”, “Fermentation flavours plant substrates” (Figure 1) generated 855, and 1700 research papers, respectively. The titles and then abstracts of these papers were screened to identify potentially relevant publications. This gave 277 and 408 initially screened articles from Web of Science and Scopus, respectively, for which the full texts were assessed against inclusion and exclusion criteria (Table 1). A technique known as “snowballing” identified a further two papers. This approach simply involved checking the reference list or the citations of an identified paper that meets the selection criteria to see if any relevant papers had been missed. At the end of this process (Figure 1), the resulting 35 papers dealing with fermented flavours of vegetable and fruit substrates were taken into consideration for this review. Note, in order to help the reader on the few occasions where a manuscript reported nomenclature that is no longer current, the name of the micro-organism(s) in question has been reported according to the currently accepted norms.

## 3. Results

### 3.1. Research Profiles

**Figure 2:**
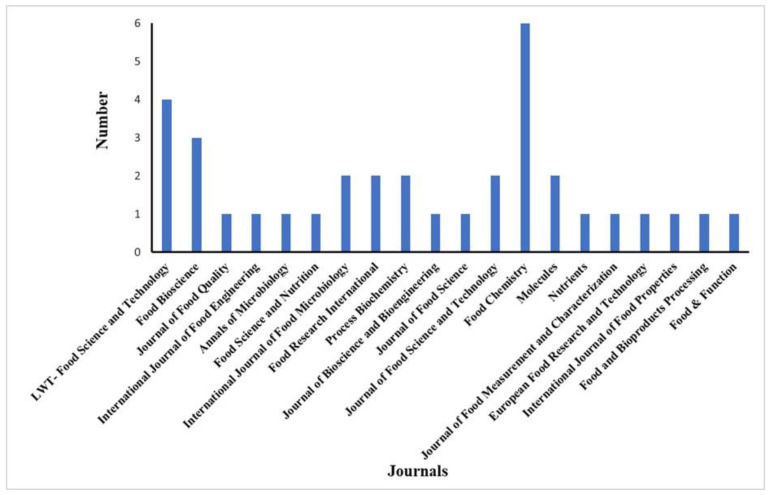
The journals in which the papers were published and the number of papers in each journal.

A total of 35 scientific papers met the criteria used for the review. An overview of the study conditions reported in the papers is presented in Table 2. The number of papers published by year was: 2017 (4 articles, 11.44%); 2018 (10 articles, 28.57%); 2019 (7 articles, 20%); 2020 (2 articles, 5.71%); 2021 (10 articles, 28.57%); and 2022 (2 articles, 5.71%). The papers were published in 20 different journals, which had an impact factor ranging from 1.713 to 7.514 (Figure 2). The greatest number of papers (6) were published in Food Chemistry.

### 3.2. Fermented Flavours of Vegetables and Fruits

Twenty-three of the thirty-five papers described VOCs originating from the fermentation of fruit juices (with 2 of the 23 on goji/wolfberry juice, 2 on jujube juice/pulp, 3 on cashew apple juice, and 3 on apple juice). Nine of the papers described VOCs originating from the fermentation of vegetable substrates (with 2 of the 9 on okara (soybean pulp)), with one investigating tomato and red pepper pomace. Three articles investigated the VOCs generated from a mixture of vegetables and mixtures of vegetables and fruits, with carrot juice being a component in all three studies and apple juice in two.

A wide range of micro-organisms were reported as being used, encompassing bacteria, LAB, fungi, and yeast. Thirty-three papers described the use of LAB, with twenty-nine studies only referring to LAB strains, one paper referring to LAB combined with other bacteria, and three papers referring to LAB and yeasts. In the remaining two studies, one study referred to the use of fungi, while the other referred to fungi in combination with yeast.

The most commonly mentioned LAB was *Lactiplantibacillus plantarum* (*L. plantarum*) (28 papers), followed by *Lacticaseibacillus casei* (*L. casei*) (12 papers), *Lactobacillus acidophilus* (*L. acidophilus*) (9 papers), *Lacticaseibacillus rhamnosus* (*L. rhamnosus*) (9 papers), *Lactobacillus helveticus* (*L. helveticus*) (4 papers), and *Streptococcus thermophilus* (*S. thermophilus*) (4 papers). Only four of the papers investigated the use of yeast in combination with LAB and fungi.

The most common fermentation temperature and time combination was 37 °C for 48 h (10 papers), as shown in Table 2. As additional examples, 9 papers described studies carried out at 37 °C with fermentation times (excluding 48 h) ranging from 20 to 120 h. Six papers described studies carried out at 30 °C with fermentation times ranging from 24 to 120 h. In 4 papers where multiple LAB strains were investigated, fermentation temperatures of 37 °C and 30 °C were used at different times.

Thirty-three of the papers isolated VOCs using headspace solid-phase microextraction (HS-SPME), and one study using a purge and trap method, while other one using a static headspace technique. HS-SPME is a simple, rapid, solvent-free method that can extract a diverse mixture of VOCs using a fibre coated with an adsorbent resin. The fibre most frequently stated as being used was 50/30 µm DVB/CAR/PDMS (Divinylbenzene-Carboxen/polydimethylsiloxane) (17 papers), followed by 75 µm CAR/PDMS (6 papers), 75 µm DVB/CAR/PDMS (1 paper), and 50/30 µm DVB/CAR (2 papers). For SPME, the most frequently reported adsorption time was 30 min (19 papers) for temperatures ranging from 35 to 85 °C, with 9 papers using 30 min at 40 °C. The remaining papers reported the use of a wide range of time and temperature combinations, ranging from 7 to 60 min at 40 to 80 °C. Gas chromatography-mass spectrometry (GC-MS) was used in 34 papers to detect the extracted VOCs, and the remaining paper reporting using gas chromatography-ion mobility spectrometry (GC-IMS).

In the papers reviewed, the main VOCs detected due to fermentation were alcohols, esters, aldehydes, ketones, acids, terpenes and norisoprenoids, sulphur compounds, phenols, furans, alkanes, alkenes, and benzene derivatives (Table 2 and Table S1). The flavour notes of the major VOCs from each of the above classes are listed in Table 3. Additional details about these different classes of compounds are presented in the following sections. Note that the type of information available from the publications reviewed ranged from the reporting of concentration values to peak area comparisons to simply reporting the presence or absence of a compound; in all cases, the maximum amount of information available in the reported studies has been presented.

### 3.3. Alcohols

Alcohols, with their characteristic aromas, comprised the largest volatile group detected in 33 of the 35 studies reviewed. Alcohols are produced from carbohydrate degradation or amino acid catabolism [52]. Across the 33 studies, the alcohols most commonly detected after fermentation were ethanol, 3-methyl-1-butanol (isoamyl alcohol/isopentyl alcohol), 2-methyl-1-butanol (amyl alcohol), 3-methyl-3-buten-1-ol (isoprenol), 2, 3-butanediol, 2-ethylhexanol, 1-hexanol, 2-hexen-1-ol, 3 hexen-1-ol, 2,6-dimethyl-4-heptanol, benzyl alcohol (phenyl methanol/benzene methanol), 2-phenylethyl alcohol (2-phenyl ethanol/benzene ethanol), 4-ethylphenol, 2-(4-methylphenyl)-2-propanol, 1-octanol, 1-octen-3-ol, (Z)-1,5-octadien-3-ol, octenol, 2-octenol, 1-nonanol, (Z)-3-nonen-1-ol, 2-undecanol, 3,7,11-trimethyl-1-dodecanol, and 2-tridecanol.

Ethanol is synthesised from sugars naturally present in plants; LAB utilise sugars via the phosphoketolase (PK) pathway, and yeast utilise sugars through the Embden–Meyerhof–Parnas (EMP) pathway [13]. In mango slurry, fermentation involving the yeast *Saccharomyces cerevisiae* (*S. cerevisiae*) generated 30–100 times more ethanol than LAB fermentation [41]. When *Williopsis saturnus* var. *saturnus* (*W. saturnus*) yeast was combined with LAB for fermentation, there was also a marked increase in the ethanol concentration (six-fold increase) compared to LAB alone in durian pulp [30]. However, ethanol can be generated by heterofermentative LAB, which possess the alcohol dehydrogenase enzyme that converts acetaldehyde into ethanol [26]. In kiwifruit juice (cultivars of *Actinidia deliciosa* cv. Xuxiang and *Actinidia chinensis* cv. Hongyang), the ethanol concentration was 10,316.5, 17,249.2, and 8652.7 ng/mL in Xuxiang cultivar juice fermented by either *L. acidophilus*, *L. helveticus,* or *L. plantarum*, respectively, compared to 6242.9 ng/mL in the unfermented juice. However, the ethanol concentration was 13,042.5, 7004.2, and 9551.9 ng/mL in Hongyang cultivar juice fermented by either *L. acidophilus*, *L. helveticus,* or *L. plantarum*, respectively, compared to 19,642.6 ng/mL in the unfermented juice [49]. The different ethanol concentrations produced from the two kiwifruit cultivars after LAB fermentation could be a result of the different substrate compositions of the cultivars, which are subjected to various metabolic pathways by LAB. After fermentation of orange pomace by *L. rhamnosus*, 0.3 µg/mL of ethanol was detected in a distillate prepared using vacuum distillation to extract VOCs. However, ethanol was not detected in a fermented orange pomace distillate prepared using a simple distillation method. In the same study, the ethanol concentration detected in distillates prepared from *L. rhamnosus* fermented melon by-product using vacuum distillation was 6.5 µg/mL, compared to 1.3 µg/mL in the distillate from the unfermented melon by-products, while ethanol was not detected in distillates prepared from the same samples using simple distillation [44]. In papaya juice, the ethanol concentration was significantly (*p* < 0.05) increased by 7 and 11 times after fermentation by either *L. plantarum* or *L. acidophilus*, respectively, compared to the concentration in the unfermented juice [23]. On the other hand, only small changes in ethanol were observed in two studies: (1) Ricci et al. [29] found that LAB fermented cherry juice had an ethanol concentration of 4.1–8.5 ng/mL, compared to 3.1–3.7 ng/mL in the unfermented juice, and (2) in watermelon juice fermented by either *L. rhamnosus, L. plantarum*, *L. casei,* or *Pediococcus pentosaceus* (*P. pentosaceus*), the ethanol concentration was 16.8, 15.2, 15.1, and 15 ng/mL, respectively, compared to 14.6 ng/mL in the unfermented juice; however, after fermentation by *Levilactobacillus brevis* (*L. brevis*), the ethanol concentration was 13.9 ng/mL [48]. Six studies reported that LAB fermentation reduced the ethanol concentration in fermented fruit and vegetable juices, compared to the unfermented juices, with the synthesis of various esters speculated to have caused this decrease: (1) In unfermented Chinese wolfberry juice, the ethanol concentration was 5501.3 µg/mL, compared to 1364.7 µg/mL in the *L. acidophilus* fermented juice, where it was not detected in the juice fermented by other LAB strains [50]; (2) in two varieties of unfermented jujube (Muzao, and Hetain) juices, the ethanol concentration was 6850, and 6130 ng/mL, respectively, compared to 5740, 5100, and 1530 ng/mL in Muzao fermented with either *L. helveticus*, *L. casei,* or *L. plantarum*, respectively, and 5380, 4400, 2660, and 2410 ng/mL in Hetain fermented with either *L. casei, L. acidophilus, L. plantarum,* or *L. helveticus*, respectively [20]; (3) in okara, the initial ethanol concentration was 44 µg/g which reduced to 20.4 and 13.8 µg/g after fermentation with LAB monoculture (*L. rhamnosus* or *Pediococcus acidilactic* (*P. acidilactic*), respectively) and to 19.6 µg/g after co-culture fermentation (*L. acidophilus*, *L. rhamnosus,* and *P. acidilactic*). However, in okara fermented with an *L. acidophilus* monoculture, the ethanol concentration increased from 44 to 57.4 µg/g [46]; (4) in unfermented apple juice, the ethanol concentration was 188.4 ng/mL, compared to 83.4–123.5 ng/mL after fermentation with various LAB strains [19]; (5) in non-pH-adjusted (2.7) sea buckthorn juice, the ethanol concentration was 170.7 ng/mL, which reduced after fermentation for 36 and 72 h by *L. plantarum* to 165.4 and 152 ng/mL, respectively. However, if the pH of the juice was adjusted to pH 3.5, the initial ethanol concentration of 166.3 ng/mL increased after *L. plantarum* fermentation for 36 and 72 h to 194.6 and 206.5 ng/mL, respectively [42]; and (6) in tomato juice, the ethanol concentration after fermentation with either *L. plantarum* or *L. casei* was 2.7 and 1.2 times lower, respectively, compared to its concentration in the unfermented juice [32].

1-Octanol is a fatty alcohol produced by micro-organisms utilizing glucose as a substrate through a fatty acid synthesis pathway using various enzymes [53]. The 1-octanol concentration was generally reported to increase after LAB fermentation in the 5 studies reviewed: (1) In Chinese wolfberry juice fermented by either *L. plantarum*, *L. casei*, *Lacticaseibacillus paracasei* (*L. paracasei*), *L. acidophilus*, *L. helveticus*, or *Bifidobacterium Lactis* (*B. lactis*), the 1-octanol concentration was 172.9, 119.1, 137.2, 209.3, 131.4, and 142.4 µg/mL, where it was not detected in the unfermented juice [50]; (2) the 1-octanol concentration in a distillate prepared using vacuum distillation from orange pomace fermented by *L. rhamnosus* was 1.5 µg/mL, compared to 0.1 µg/mL in the distillate from unfermented pomace; in distillates prepared using a simple distillation method, the 1-octanol concentration in the fermented orange pomace distillate was 2.1 µg/mL, compared to 1.8 µg/mL in the unfermented pomace distillate. Interestingly in the same study, using the simple distillation method, the 1-octanol concentration in the fermented melon by-product distillate was 21.5 µg/mL, compared to 7.1 µg/mL in the unfermented by-product distillate, whereas in extracts prepared by vacuum distillation, the 1-octanol concentration in the distillate from melon by-product fermented by *L. rhamnosus* was 1.1 µg/mL, compared to 0.2 µg/mL in the unfermented by-product distillate [44]; (3) in kiwifruit juice (Xuxiang and Hongyang cultivars), the 1-octanol concentration was 285.5 and 325.5 ng/mL in Xuxiang cultivar juice fermented by either *L. helveticus* or *L. plantarum*, respectively, compared to 146.4 ng/mL in the unfermented juice, where it was not detected in Xuxiang cultivar juice fermented by *L. acidophilus*. Interestingly, with the Hongyang cultivar juice, 1-octanol was not detected in the unfermented juice or in any of the LAB fermented juices. [49]; (4) in cherry juice fermented by either *L. plantarum*, *L. rhamnosus,* or *L. paracasei*, the 1-octanol concentration was 4.5–7.8, 8.4, and 5.2 ng/mL, respectively, compared to 3.4–3.6 ng/mL in the unfermented juice [29]; and (5) in apple juice fermented by either *L. acidophilus*, *L. rhamnosus*, *L. casei* or *L. plantarum*, the 1-octanol concentration was 4.2, 3.5, 3.8, and 4.0 ng/g, respectively, compared to 1.0 ng/g in the unfermented juice [21]. However, in okara fermented by LAB monoculture of either *L. rhamnosus*, *P. acidilactic* or co-culture (*L. acidophilus*, *L. rhamnosus,* and *P. acidilactic*), the 1-octanol concentration was 11.7, 17.8, and 1.7 µg/g, respectively, compared to 30.0 µg/g in the unfermented okara, whereas okara fermented with *L. acidophilus* had an 1-octanol concentration of 34.7 µg/g [46].

1-Hexanol is produced from the enzymatic oxidation of the fatty acid linoleic acid [54]. In 11 experiments, the 1-hexanol concentration increased after fermentation: (1) In Chinese wolfberry juice fermented by either *L. paracasei*, *L. acidophilus*, or *B. lactis*, the 1-hexanol concentration was 566.3, 728.2, and 682.2 µg/mL, respectively, where it was not detected in the unfermented juice and the juice fermented by other LAB strains [50]; (2) for kiwifruit juice (Xuxiang, and Hongyang cultivars), the 1-hexanol concentration in the Xuxiang cultivar juice fermented by either *L. acidophilus*, *L. helveticus,* or *L. plantarum* was 11,239.2, 13,280.9, and 11,713.8 ng/mL, respectively, compared to 7054.8 ng/mL in the unfermented juice, where for the Hongyang cultivar juice fermented by either *L. acidophilus*, *L. helveticus,* or *L. plantarum*, the 1-hexanol concentration was 12,313.5, 12,357.1, and 11,461.1 ng/mL, respectively, compared to 2462.9 ng/mL in the unfermented juice [49]; (3) the 1-hexanol concentration in a distillate prepared using simple distillation of orange pomace fermented by *L. rhamnosus* was 1.1 µg/mL, compared to 0.1 µg/mL in the unfermented orange pomace distillate. In the same study, using simple distillation, the 1-hexanol concentration in fermented melon by-product distillate was 21.2 µg/mL, compared to 10.2 µg/mL in the unfermented melon by-product distillate, whereas, when using vacuum distillation, the 1-hexanol concentration in fermented melon by-product distillate was 4.3 µg/mL, compared to 0.4 µg/mL in the unfermented melon by-product distillate [44]; (4) in watermelon juice fermented by either *L. rhamnosus, L. casei, L. plantarum*, *L. brevis,* or *P. pentosaceus*, the 1-hexanol concentration was 79.9, 121.0, 141.0, 170.0, and 171.0 ng/mL, respectively, compared to 74.7 ng/mL in the unfermented juice [48]; (5) in cherry juice fermented by either *L. plantarum*, *L. rhamnosus,* or *L. paracasei*, the 1-hexanol concentration was 1.9–3.5, 2.1, and 1.1 ng/mL, respectively, compared to 0.7–0.8 ng/mL in the unfermented juice [29]; (6) in okara fermented by either *L. acidophilus, L. rhamnosus,* or *P. acidilactic*, the 1-hexanol concentration was 206.7, 217, and 217.5 µg/g, respectively, compared to 64.2 µg/g in the unfermented okara. However, for okara fermented with LAB co-cultures (*L. acidophilus*, *L. rhamnosus*, and *P. acidilactic*), the 1-hexanol concentration reduced to 16.0 µg/g [46]; (7) in jujube juice fermented by a mixture of *L. plantarum*, *L. rhamnosus*, and *S. thermophilus*, the 1-hexanol concentration was 824 ng/g, and it was not detected in the unfermented juice [47]; (8) in apple juice fermented by either *L. plantarum*, *L. rhamnosus, L. acidophilus,* or *L. casei*, the 1-hexanol concentration was 21.1, 22.0, 47.4, and 52.4 ng/g, respectively, compared to 1.2 ng/g in the unfermented juice [21]; (9) in grape juice fermented by LAB, the 1-hexanol concentration was 3.3-fold higher compared to the concentration in the unfermented juice [43]; (10) in papaya juice fermented by either *L. acidophilus* or *L. plantarum*, the 1-hexanol concentration was 3 and 4 times higher, respectively, compared to its concentration in the unfermented juice [23]; and (11) in *Momordica charantia* juice fermented by *L. plantarum*, the 1-hexanol concentration was 2.5 times higher compared to the concentration in the unfermented juice [18]. However, the 1-hexanol concentration has also been reported to reduce after fermentation in 3 studies: (1) In goji juice fermented by different combinations of bacterial strains (either *L. plantarum*, *L. rhamnosus*, *Limosilactobacillus reuteri* (*L. reuteri*), *Bacillus velezensis* (*B. velezensis*), or *Bacillus licheniformis* (*B. licheniformis*)), the 1-hexanol concentration was 43.0–61.4 ng/g, compared to 80.2 ng/g in the unfermented juice [25]; (2) in tomato juice fermented by *L. plantarum*, the 1-hexanol concentration was reduced by half, compared to its concentration in the unfermented juice [32]; and (3) in mung bean fermented by *L. plantarum*, 1-hexanol was not detected, where it was detected in the unfermented bean [45].

Benzyl alcohol is an aromatic alcohol produced by microbial fermentation either from glucose [55] or the amino acid phenylalanine [56]. Benzyl alcohol was detected after bacterial fermentation in 7 studies [19,25,29,34,35,45,47] with concentrations ranging from 2.3 ng/mL to 270 ng/mL.

3-Methyl-1-butanol and 2-phenylethyl alcohol were the most common amino acid-derived alcohols detected after fermentation. 3-Methyl-1-butanol and 2-phenylethyl alcohol are synthesised from the catabolism of the amino acids leucine [57] and phenylalanine [58], respectively. 3-Methyl-1-butanol has a malt/alcoholic/whiskey odour that is considered unpleasant when present in concentrations greater than 400 µg/mL [59]. In fermented fruit and vegetable juices, 3-methyl-1-butanol was detected in 10 studies [21,22,23,24,29,31,38,44,45,49]. However, in fermented fruit and vegetable juices, the 3-methyl-1-butanol concentration was reduced in 5 studies: (1) In Chinese wolfberry juice fermented by either *L. casei*, *L. paracasei*, *L. acidophilus*, *L. helveticus,* or *B. lactis*, the 3-methyl-1-butanol concentration was 825.9, 674.0, 833.6, 799.6, and 820.6 µg/mL, respectively, compared to 2065.3 µg/mL in the unfermented juice, and it was not detected in the juice fermented by *L. plantarum* [50]; (2) in non-pH-adjusted (2.7) sea buckthorn juice, the 3-methyl-1-butanol concentration was 122.9 ng/mL, which was reduced after fermentation for 36 and 72 h by *L. plantarum* to 98.9 and 91.4 ng/mL, respectively. However, if the pH of the juice was adjusted to pH 3.5, the initial 3-methyl-1-butanol concentration of 121.4 ng/mL increased after *L. plantarum* fermentation for 36 and 72 h to 217.9 and 233 ng/mL, respectively [42]; (3) in apple juice fermented by different LAB strains, the 3-methyl-1-butanol concentration ranged from 4.5–16.9 ng/mL, compared to 73.2 ng/mL in the unfermented juice, among the strains studied, *L. acidophilus* fermented juice had a 93% reduction in 3-methyl-1-butanol concentration [19]; (4) in apple juice fermented by the yeast *S. cerevisiae*, the 3-methyl-1-butanol concentration was 644 ng/mL; however, the concentration of 3-methyl-1-butanol reduced to 42.1 ng/mL after *L. plantarum* sequential fermentation [51]; and (5) in cashew apple juice fermented by *L. acidophilus*, the 3-methyl-1-butanol concentration reduced by 2 times, compared to other LAB strains (either *L. plantarum* or *L. casei*) studied [26].

In 10 studies, the concentration of 2-phenylethyl alcohol, which has a flowery smell, was increased or it was detected after fermentation: (1) In Chinese wolfberry juice fermented by *L. plantarum*, the 2-phenylethyl alcohol concentration was 246.4 µg/mL, where it was not detected in the unfermented juice or the fermented juice by other LAB strains [50]; (2) in non-pH-adjusted (2.65) bog bilberry juice fermented by two strains of *L. plantarum*, the 2-phenylethyl alcohol concentration was 1731.2 and 1775.8 ng/mL, compared to 663.5 ng/mL in the unfermented juice. However, if the pH of the juice was adjusted to pH 3.5, the initial 2-phenylethyl alcohol concentration of 617.5 ng/mL was decreased after fermentation by two strains of *L. plantarum* to 459.7 and 463.4 ng/mL [34]; (3) in grape juice fermented by LAB, the 2-phenylethyl alcohol concentration was 40.6 ng/mL, where it was not detected in the unfermented juice [43]; (4) in horse gram sprouts fermented by two *L. plantarum* strains, the 2-phenylethyl alcohol concentration was 1290 and 780 ng/g, compared to 40 ng/g in raw seed [37]; (5) in goji juice fermented by different combinations of bacterial strains (either *L. plantarum*, *L. rhamnosus*, *L. reuteri*, *B. velezensis,* or *B. licheniformis*), the 2-phenylethyl alcohol concentration ranged from 362.6 to 494.0 ng/g, compared to 103.1 ng/g in the unfermented juice [25]; (6) in jujube pulp fermented by a mixture of *L. plantarum*, *L. rhamnosus*, and *S. thermophilus*, the 2-phenylethyl alcohol concentration was 283 ng/g, where it was not detected in the unfermented juice [47]; (7) in okara fermented with a monoculture of *Rhizopus oligosporus* (*R. oligosporus*) fungi, the 2-phenylethyl alcohol concentration increased by 20 times, compared to its concentration in the unfermented okara, whereas with a mixed culture of *R. oligosporus* fungi and *Yarrowia lipolytica* (*Y. lipolytica*) yeast, the concentration increased by 8.5 times [38]; (8) in mango slurry fermented by yeast *S. cerevisiae*, the 2-phenylethyl alcohol concentration was 4 to 23 times higher, compared the concentration after LAB fermentation, where it was not detected in the unfermented mango slurry [41]; (9) in papaya juice fermented by *L. plantarum*, the 2-phenylethyl alcohol concentration was doubled, compared to the concentration in *L. acidophilus* fermented juice, where it was not detected in the unfermented juice [23]; and (10) in durian pulp fermented by *L. casei* mixed with the yeast *W. saturnus*, 2-phenylethyl alcohol was detected, where it was not detected in the unfermented pulp, or in the pulp fermented by a *L. casei* monoculture [30].

Furthermore, compared to unfermented juice, one study reported that almost half of the alcohols detected decreased in *L. casei* fermented tomato juice, likewise in *L. plantarum* fermented tomato juice, most of the alcohols detected decreased. However, due to the generation of new alcohols, the relative peak area (RPA) for total alcohols increased to 59.9% and 49.7% in juice fermented by either *L. casei* or *L. plantarum*, respectively, compared to a 49.3% RPA in the unfermented juice [32]. On the other hand, LAB fermentation increased the overall combined alcohol concentration of fruit and vegetable juices in 4 studies: (1) LAB fermentation of apple juice increased the overall combined alcohol concentration by 10 times compared to its concentration in the unfermented juice, demonstrating that most of the alcohols were produced during fermentation [21]; (2) LAB fermentation of grape juice increased the total combined alcohol concentration by 102.4% [43]; (3) LAB fermentation of kiwifruit juice (Xuxiang and Hongyang cultivars) increased the total combined alcohol concentration by 39, 107, and 56% in Xuxiang cultivar juice fermented by either *L. acidophilus*, *L. helveticus,* or *L. plantarum*, respectively, and by 25, 30, and 26% in Hongyang cultivar juice fermented by either *L. acidophilus*, *L. helveticus* or *L. plantarum*, respectively [49]; and (4) LAB fermentation increased the total combined alcohol concentration of jujube juice (Varieties of Muzao and Hetian) by 66.5% in *L. acidophilus* fermented Muzao juice and 33.7% in *L. casei* fermented Hetian juice [20]. In another study, the total combined alcohol concentration of mango slurry fermented with yeast strains was nearly 10 times higher compared to mango slurry fermented with LAB strains [41].

Overall, alcohols such as 1-hexanol (11 papers), 3-methyl-1-butanol (10 papers), 2-phenylethyl alcohol (10 papers), benzyl alcohol (7 papers), ethanol (7 papers), and 1-octanol (5 papers) have been reported to have increased or were only detected after the fermentation of fruit and vegetable juices, mainly by LAB. However, it is important to note that for some substrates, the concentration of ethanol (6 papers), 3-methyl-1-butanol (5 papers), 1-hexanol (3 papers), and 1-octanol (1 paper) has been reported to have decreased after fermentation.

### 3.4. Esters

Esters, which have sweet and fruity notes, are formed when carboxylic acids linked with coenzyme-A (CoA) are esterified with alcohols [60]. Volatile esters were found in fermented fruit and vegetable juices in 27 of the 35 papers reviewed in this report. The sensory detection threshold for esters is lower than that of the corresponding alcohol or acid [61]. The majority of esters reported were either ethyl esters or acetate esters. Ethanol and acyl-CoA derivatives of fatty acids combine to form ethyl esters. Acetyl-CoA and alcohols, such as ethanol or higher alcohols, produced from amino acid metabolism, combine to form acetate esters [62]. The key ester compounds reported in the reviewed studies were the acetate esters: ethyl acetate (ethyl ethanoate); 3-methylbutyl acetate (isoamyl acetate/isopentyl acetate); 2-phenylethyl acetate and hexyl acetate, and ethyl esters: ethyl butanoate (ethyl butyrate); ethyl-3-methyl butanoate (ethyl isovalerate/ethyl isopentanoate); ethyl hexanoate (ethyl caproate); ethyl octanoate (ethyl caprylate); ethyl dodecanoate (ethyl laurate); ethyl propanoate (ethyl propionate); ethyl-2-methyl-butanoate; ethyl hexadecanoate (ethyl palmitate) and others: hexyl formate; methyl 3-methylbutanoate (methyl isovalerate/methyl isopentanoate); 3-methylbutyl 3-methylbutanoate (isoamyl isovalerate/isopentyl isopentanoate) and methyl 2-hydroxybenzoate (methyl salicylate).

Eleven of the papers stated that ethyl acetate, which is formed by alcohol acetyltransferases from the reaction between acetyl Co-A and ethanol [20], was primarily responsible for the fruity flavour of fermented fruits and vegetable juices. A variety of LAB strains [19,20,21,22,23,33,44,45,50] were used in all studies except two; one used LAB combined with a yeast [30], and the other used fungi combined with a yeast [38]. In these studies, it was reported that for Chinese wolfberry juice fermented by either *L. plantarum*, *L. paracasei*, *L. acidophilus,* or *L. helveticus*, the ethyl acetate concentration was 6931, 4827.1, 4925.4, and 7323.3 µg/mL, respectively, compared to 774.5 µg/mL in the unfermented juice, where it was not detected in the juice fermented by *L. casei* or *B. lactis* [50]. In Muzao jujube juice fermented by either *L. plantarum* or *L. acidophilus*, the ethyl acetate concentration was 111.7 and 64.2 µg/mL, respectively, where it was not detected in the unfermented juice or juice fermented by other LAB [20]. Further, in durian pulp fermented by *L. casei* combined with a yeast *W. saturnus*, ethyl acetate was detected, whereas it was not detected in sole *L. casei* fermentation or unfermented pulp [30]. However, Liu et al. [32] found that in tomato juice, prior to fermentation, ethyl acetate was detected, where it was not detected after fermentation by LAB, and in mango slurry fermented by either yeast or LAB, the ethyl acetate concentration was reduced by 1.2–1.8 times, compared to its concentration in the unfermented slurry [41].

In 3 experiments, ethyl butanoate, which is formed by a reaction between ethanol and butyryl-CoA during LAB fermentation [21], was the next most common ester compound: (1) The ethyl butanoate concentration in a distillate prepared using simple distillation of *L. rhamnosus* fermented melon by-product was 8760 ng/mL, compared to 700 ng/mL in the unfermented melon by-product distillate, and the ethyl butanoate concentrations of both the fermented and unfermented by-product distillates produced using vacuum distillation were at least 10 times lower than in the simple distillation distillates [44]; (2) in apple juice fermented by different LAB, the ethyl butanoate concentration ranged from 16.3 to 23.1 ng/g, compared to 2.1 ng/g in the unfermented juice [21]; and (3) in papaya juice fermented by LAB, ethyl butanoate was detected, where it was not detected in the unfermented juice [23].

2-Phenylethyl acetate, which is formed by a reaction between 2-phenylethyl alcohol and acetyl CoA, was the third most commonly reported (3 studies) ester in juices after fermentation: (1) In horse gram sprouts fermented by *L. plantarum*, the 2-phenylethyl acetate concentration was 220 ng/g, where it was not detected in raw seeds [37]; (2) in grape juice fermented by a mixed culture of *L. plantarum* and *L. brevis*, the 2-phenylethyl acetate concentration was 3.5 ng/mL, compared to its concentration in the unfermented juice (1.6 ng/mL) [43]; and (3) in durian pulp fermented by a combination of *L. casei* and *W. saturnus* yeast, 2-phenylethyl acetate was detected, whereas it was not detected during *L. casei* only fermentation or in the unfermented pulp [30].

Propyl acetate, which is formed by a reaction between propanol and acetyl-CoA, was detected in cherry juice fermented by either *L. plantarum*, *L. rhamnosus*, or *L. paracasei*, where the propyl acetate concentration was 18.5–83.4, 1186.7, and 201.6 ng/mL, respectively, compared to about 0.01 ng/mL in the unfermented juice. In this study, the formation of propyl acetate during fermentation appeared to correlate with acetic acid production. As there was a low concentration of acetic acid after fermentation by *L. rhamnosus* or *L. paracasei*, this was taken as evidence of the conversion of acetic acid to the corresponding ester. In contrast, in the same study, fermentation by *L. plantarum* resulted in a high concentration of acetic acid and a lower concentration of propyl acetate [29].

Overall the total combined ester concentration increased in 6 studies after fermentation due to the availability of alcohol precursors [52]: (1) In Muzao jujube juice fermented by either *L. acidophilus* or *L. plantarum*, the total combined ester concentration was 65.4 and 156.7 µg/mL, respectively, compared to 4.7 µg/mL in the unfermented juice [20]; (2) in mixed juices (apple juice, orange juice, carrot juice, and Chinese jujube juice) fermented by LAB mixed culture (*L. plantarum*, *Bifidobacterium breve* (*B. breve*), and *S. thermophilus*), the total combined ester concentration was 415 ng/mL, compared to 239 ng/mL in the unfermented juice [35]; (3) in apple juice fermented by different LAB, the total combined ester concentration ranged from 81.8 to 92.9 ng/g, compared to 33.7 ng/g in the unfermented juice [21]; (4) in grape juice fermented by LAB, the total combined ester concentration increased by 83.76%, compared to its concentration in the unfermented juice [43]; (5) in mango slurry fermented by LAB and yeast, the total combined ester concentration increased, compared to the unfermented slurry, with the yeast *S. cerevisiae* generating a significantly (*p* < 0.05) higher number of esters present at a high concentration than LAB [41]; and (6) in pomegranate juice fermented by LAB, the total combined ester concentration increased, compared to the concentration in the unfermented juice [24].

On the other hand, 3 studies reported a reduction in the total combined ester concentration after fermentation, possibly due to hydrolysis into their corresponding acids and alcohols [47]: (1) In apple juice fermented by either *L. plantarum*, *L. helveticus*, *L. casei*, *L. paracasei*, *L. acidophilus* or *B. lactis*, the total combined ester concentration was 1090, 1279.6, 787.5, 695.9, 702.3, and 643.1 ng/mL, respectively, compared to 1410.7 ng/mL in the unfermented juice [19]; (2) in jujube juice fermented by a mixture of *L. plantarum*, *L. rhamnosus*, and *S. thermophilus*, the total combined ester concentration was 1541 ng/g, compared to 5814 ng/g in the unfermented juice [47]; and (3) in tomato juice fermented by either *L. casei* or *L. plantarum*, the total combined ester concentration was 1.6 times and 7 times lower, respectively, compared to the concentration in the unfermented juice [32].

Overall, esters such as ethyl acetate (11 papers), ethyl butanoate (3 papers), 2-phenylethyl acetate (3 papers), and propyl acetate (1 paper) have been reported to increase or were only detected after fermentation of fruit and vegetable juices, mainly by LAB. However, the concentration of ethyl acetate did decrease after fermentation in 2 studies.

### 3.5. Ketones

A number of ketones were identified in 26 studies investigating the fermentation of vegetable and fruit juices. 3-Hydroxy-2-butanone (acetoin), 2,3-butanedione (diacetyl), 2-propanone (acetone), 1-hydroxy-2-propanone (hydroxy acetone), 2-butanone (methyl ethyl ketone), 2-pentanone, 2-hydroxy-3-pentanone, 3-methyl-4-methylene-2-hexanone, 2-heptanone, 4-heptanone, 4-methyl-2-heptanone, 6-methyl-5-hepten-2-one (sulcatone), 2-octanone, 2-nonanone, 2-dodecanone, 2-undecanone, 2-tridecanone, 2-tetradecanone, (E)-6,10-dimethylundeca-5,9-dien-2-one (geranyl acetone), 4-cyclopentene-1,3-dione, and 1-phenylethanone (acetophenone) were identified in the reviewed papers.

**Figure 3:**
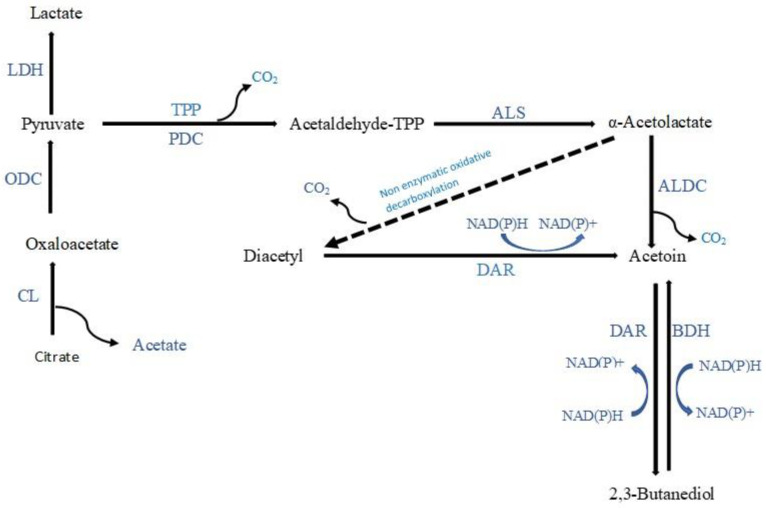
Citrate metabolic pathway by LAB. CL: citrate lyase, ODC: oxaloacetate decarboxylase, LDH: lactate dehydrogenase, PDC: pyruvate decarboxylase, TPP: thiamine pyrophosphate, ALS: α-acetolactate synthase, ALDC: α-acetolactate decarboxylase, DAR: diacetyl acetoin reductase, and BDH: 2,3-butanediol dehydrogenase [63,64,65,66].

**Figure 4:**
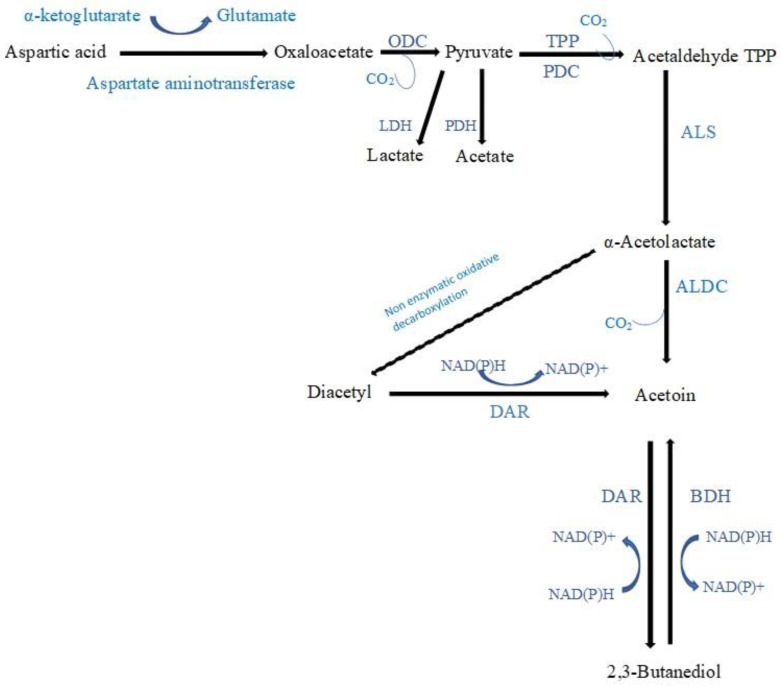
Catabolism of aspartic amino acid by *Lactococcus lactis.* ODC: oxaloacetate decarboxylase, LDH: lactate dehydrogenase, PDH: pyruvate dehydrogenase, PDC: pyruvate decarboxylase, TPP: thiamine pyrophosphate, ALS: α-acetolactate synthase, ALDC: α-acetolactate decarboxylase, DAR: diacetyl acetoin reductase, and BDH: 2,3-butanediol dehydrogenase [67].

3-Hydroxy-2-butanone (acetoin), which imparts a creamy/buttery note, was the most frequently detected ketone produced during the fermentation of vegetables and fruits. Citrate in vegetable and fruit juices can be directly converted to acetoin (Figure 3) by some LAB strains exhibiting citrate permease and citrate lyase activities. Citrate can be converted by LAB to pyruvate via oxaloacetate, then to acetaldehyde-thiamine pyrophosphate (TPP) through a decarboxylation process, and finally to acetaldehyde-TPP through an enzymatic reaction involving α-acetolactate synthase, resulting in the synthesis of α-acetolactate. α-Acetolactate synthase has a low affinity for pyruvate; therefore, an excess of pyruvate is required to favour this reaction. In the presence of citrate and sugars, homofermentative LAB will convert pyruvate directly to α-acetolactate when less NADH is generated than pyruvate. Heterofermentative LAB will, however, accumulate pyruvate at low pH when citrate is the sole carbon source. Further, due to the instability of α-acetolactate, it is readily decarboxylated enzymatically or chemically to yield acetoin. Acetoin can also be synthesised from diacetyl via the enzyme diacetyl acetoin reductase [63,64,65,66]. In addition, when the pH of the medium is between 5 and 8, *Lactococcus lactis* can also produce acetoin from the catabolism of aspartic amino acid [67] (Figure 4). The acetoin concentration increased after LAB fermentation of fruit and vegetable juices in 8 studies: (1) In Chinese wolfberry juice fermented by either *L. plantarum*, *L. paracasei*, *L. helveticus*, or *B. lactis*, the acetoin concentration was 346.3, 267.4, 528.1, and 422.1 µg/mL, respectively, compared to 29.1 µg/mL in the unfermented juice, where it was not detected in the juice fermented by *L. casei* or *L. acidophilus* [50]; (2) in Muzao jujube juice fermented either *L. plantarum* or *L. helveticus*, the acetoin concentration was 29.9 and 30.8 µg/mL, respectively, compared to 17.5 µg/mL in the unfermented juice. However, the acetoin concentration was reduced to 10.5 µg/mL in *L. acidophilus* fermented juice and acetoin was not detected in *L. casei* fermented juice [20]; (3) in kiwifruit juice (Xuxiang, and Hongyang cultivars), the acetoin concentration was 2621.6 and 1348.9 ng/mL in the Xuxiang cultivar juice fermented by either *L. helveticus* or *L. plantarum*, respectively, where it was not detected in the *L. acidophilus* fermented juice or in the unfermented juice. The acetoin concentration was 8431.7 and 4390.6 ng/mL in Hongyang cultivar juice fermented by either *L. helveticus* or *L. plantarum*, respectively, where it was not detected in the *L. acidophilus* fermented juice or in the unfermented juice [49]; (4) in elderberry juice fermented by either *L. plantarum*, *L. casei*, or *L. rhamnosus*, the acetoin concentration was 83.1–496.7, 90.7–314.5, and 41.4–456.2 ng/mL, respectively, compared to 1.4–22.1 ng/mL in the unfermented juice [22]; (5) in cherry juice fermented by either *L. rhamnosus* or *L. paracasei*, the acetoin concentration was 260.7 and 5.9 ng/mL, respectively, compared to 0.001 ng/mL in the unfermented juice, and in cherry juice fermented by different *L. plantarum* strains, the acetoin concentration ranged from 44 to 287.9 ng/mL, compared to 0.002 ng/mL in the unfermented juice [29]; (6) the acetoin concentration in a distillate prepared using vacuum distillation from orange pomace fermented by *L. rhamnosus* was 450 ng/mL, compared to 110 ng/mL in the unfermented pomace distillate, where acetoin was not detected in distillates from either fermented or unfermented pomace using the simple distillation method [44]; (7) in mung beans fermented by two *L. plantarum* strains, the acetoin concentration was 2.8 and 7.5 times higher, compared to the concentration in the unfermented mung beans [45]; and (8) in papaya juice fermented by either *L. plantarum* or *L. acidophilus*, the acetoin concentration was 2.2 and 3.7 times higher, respectively, compared to the unfermented juice [23]. In 5 studies, acetoin was only detected after fermentation of juices: (1) In okara fermented by a LAB co-culture (*L. acidophilus*, *L. rhamnosus*, and *P. acidilactici*), the acetoin concentration was 166.3 µg/g, where it was not detected in LAB monocultures [46]; (2) in horse gram sprouts fermented by *L. plantarum*, the acetoin concentration was 440 ng/g [37]; (3) in goji juice fermented by a bacterial mixture of *L. rhamnosus*, *L. reuteri*, and *B. velezensis*, the acetoin concentration was 87.2 ng/g juice [25]; (4) in mango slurry fermented by *S. thermophilus*, acetoin was detected, where it was not detected after fermentation by yeast *S. cerevisiae* or other LAB [41]; and (5) in durian pulp fermented by *L. casei* in sequential co-culture with *W. saturnus* yeast, acetoin was detected, where it was not detected in *L. casei* monoculture [30]. Note, that in apple juice fermented by either *L. acidophilus*, *L. helveticus,* or *L. paracasei*, the acetoin concentration was reported to have decreased to 0.8 ng/mL, 1.2 ng/mL, and 4.3 ng/mL, respectively, compared to 5.4 ng/mL in the unfermented juice, where it was not detected in *L. plantarum*, *L. casei*, or *B. lactis* fermented apple juice [19].

The second most commonly reported ketone was 2,3-butanedione (diacetyl), which imparts creamy/buttery notes. It is produced by LAB from citrate present in juice (Figure 3). As discussed for the acetoin production pathway, α-acetolactate can be directly converted into diacetyl through nonenzymatic oxidative carboxylation in the presence of molecular oxygen [63,65]. Diacetyl was reported to be increased by the presence of some LAB during fermentation of juices in 6 studies: (1) In Chinese wolfberry juice fermented by either *L. plantarum*, *L. casei*, *L. paracasei*, or *L. acidophilus*, the diacetyl concentration was 45.1, 51, 71.9, and 28.1 µg/mL, respectively, where it was not detected in the unfermented juice nor the juice fermented by *L. helveticus* or *B. lactis* [50]; (2) in elderberry juice fermented by either *L. rhamnosus*, *L. plantarum*, or *L. casei* strains, the diacetyl concentration ranged from 37–586.8, 16.2–400.7, and 221.9–276.6 ng/mL, respectively, compared to 3.3–12.2 ng/mL in the unfermented juice [22]; (3) in kiwifruit juice (Xuxiang, and Hongyang cultivars), the diacetyl concentration was 261.1 ng/mL in Xuxiang cultivar juice fermented by *L. helveticus*, where it was not detected in Xuxiang cultivar juice fermented by other LAB or in the unfermented juice. Interestingly, with the Hongyang cultivar juice, diacetyl was not detected in the unfermented juice or in any of the LAB fermented juices [49]; (4) in watermelon juice fermented by either *L. plantarum*, *L. brevis*, *L. casei,* or *L. rhamnosus*, the diacetyl concentration was 1.46, 1.47, 62.5, and 85.7 ng/mL, respectively, where it was not detected in the *P. pentosaceus* fermented juice and the unfermented juice [48]; (5) in mango slurry fermented by *L. casei*, diacetyl was detected, whereas it was not detected in other LAB or yeast fermentations [41]; and (6) in pomegranate juice fermented by *L. plantarum* strains, the diacetyl concentration increased, compared to the concentration in the unfermented juice [24]. However, in tomato and pepper pomace fermented by either *Trichoderma atroviride* (*T. atroviride*) or *Aspergillus sojae* (*A. sojae*), the diacetyl concentration was reduced compared to the concentration present in the unfermented pomace [40].

Overall, after LAB fermentation, the total combined ketone concentration increased in 5 studies: (1) In okara fermented by LAB co-culture with *L. acidophilus*, *L. rhamnosus*, and *P. acidilactici*, the total combined ketone concentration was 2355.6 µg/g, compared to 116.1 µg/g in the unfermented okara; however, in okara fermented by monocultures of either *L. acidophilus*, *L. rhamnosus,* or *P. acidilactici*, the total combined ketone concentration was 98.8, 64.3, and 57.8 µg/g, respectively. During a monoculture fermentation, unstable aldehydes and ketones may be reduced to primary and secondary alcohols, whereas in a co-culture fermentation, synergic interactions between strains may instead result in the production of higher levels of ketones, which could be linked to the oxidation of alcohols [46]; (2) in cherry juice fermented by either *L. rhamnosus* or *L. paracasei*, the total combined ketone concentration was 285.1 and 11.3 ng/mL, respectively, compared to 7.2 ng/mL in the unfermented juice. In the cherry juice fermented by different *L. plantarum* strains, the total combined ketone concentration ranged from 48.3 to 292.4 ng/mL, compared to 6.9 ng/mL in the unfermented juice [29]; (3) in apple juice fermented by either *L. plantarum*, *L. helveticus*, *L. casei*, *L. paracasei*, *L. acidophilus,* or *B. lactis*, the total combined ketone concentration was 17.0, 27.6, 29.6, 56.6, 22.5, and 26.4 ng/mL, respectively, compared to 16.6 ng/mL in the unfermented juice [19]; (4) in another study, using apple juice fermented by different LAB strains, the total combined ketone concentration ranged from 10.1 to 11.7 ng/g, compared to 2.6 ng/g in the unfermented juice [21]; and (5) LAB fermentation of kiwifruit juice (Xuxiang, and Hongyang cultivars) increased the total combined ketone concentration by 2.6, 5.2, and 2.6 times in Xuxiang cultivar juice fermented by either *L. acidophilus*, *L. helveticus*, or *L. plantarum*, respectively, and by 6.3, 75, and 37 times in Hongyang cultivar juice fermented by either *L. acidophilus*, *L. helveticus*, or *L. plantarum*, respectively [49]. Moreover, in mango slurry fermented by LAB, the total combined ketone concentration was 1.2–1.8 times higher compared to the concentration in the unfermented juice, whereas in mango slurry fermented by the yeast *S. cerevisiae*, the total combined ketone concentration was 1.4–2.7 times lower compared to the concentration in the unfermented juice [41].

Overall, ketones, which are key contributors to dairy notes, such as acetoin (13 papers) and diacetyl (6 papers) have been reported to increase or were only detected after the fermentation of fruit and vegetable juices. However, in two studies, the concentration of acetoin (1 paper), and diacetyl (1 paper) decreased after fermentation by LAB and fungi, respectively.

In 8 studies, *L. plantarum* was the main LAB producing acetoin in fermented juices, followed by *L. helveticus* (3 studies) and *L. rhamnosus* (3 studies). The two main LAB that produced high diacetyl concentrations in fermented juices were *L. plantarum* (4 papers) and *L. casei* (4 papers). Overall, for the papers reviewed, *L. plantarum* produced more of the creamy flavours of acetoin and diacetyl compared to other LAB strains studied.

### 3.6. Aldehydes

Aldehydes were present at lower concentrations after the fermentation of fruit and vegetables in 26 out of 35 studies. During the fermentation process, aldehydes are generated via alcohol oxidation or acid decarboxylation. The main aldehyde compounds detected after fermentation included ethanal (acetaldehyde), phenyl methanal (benzaldehyde), 2-methyl butanal, 3-methyl butanal (isovaleraldehyde), pentanal (valeraldehyde), hexanal (caproaldehyde), (E)-2-hexenal, heptanal (enanthaldehyde), octanal (caprylaldehyde), (E)-2-octenal, nonanal (pelargonaldehyde), (E)-2-nonenal, decanal (capraldehyde), dodecanal (lauraldehyde), 2,4-dimethyl-benzaldehyde, octadecanal (stearaldehyde), 2-undecenal, tridecanal, 3,5-dimethyl-benzaldehyde, and benzeneacetaldehyde/phenylacetaldehyde.

Acetaldehyde provides fermented juices their distinct flavour, and it is produced by LAB from the amino acid threonine [68] or from sugars via the PK (phosphoketolase) pathway, and by yeast from sugars via the EMP (Embden–Meyerhof–Parnas) pathway [13]. At lower concentrations, acetaldehyde improves the flavour of fermented juice; however, at higher concentrations (200 µg/g or 200 µg/mL or above) [20,21], it may negatively influence the flavour of fermented juices. Acetaldehyde was detected in 6 studies after LAB fermentation: (1) In Muzao jujube juice fermented by *L. acidophilus*, the acetaldehyde concentration was 19.9 µg/mL, compared to 1.5 µg/mL in the unfermented juice, with other LAB strains generating slightly higher or lower acetaldehyde concentrations compared to the unfermented juice [20]; (2) in kiwifruit juice (Xuxiang and Hongyang cultivars), Xuxiang cultivar juice fermented by either *L. plantarum*, *L. acidophilus*, or *L. helveticus* the acetaldehyde concentration was 1013.1, 136.1, and 124.3 ng/mL, respectively, compared to 109.5 ng/mL in the unfermented juice, whereas in the Hongyang cultivar juice fermented by either *L. plantarum* or *L. acidophilus*, the acetaldehyde concentration was 1075.6 and 159.7 ng/mL, respectively, compared to 95.7 and 25.5 ng/mL in the unfermented juice and fermented juice by *L. helveticus*, respectively [49]; (3) in apple juice fermented by either *L. casei, L. rhamnosus*, *L. plantarum*, or *L. acidophilus*, the acetaldehyde concentration was 40.4, 15.0, 27.5, and 21.9 ng/g, respectively, whereas it was not detected in the unfermented juice [21]; (4) in apple juice fermented by either *L. plantarum*, *L. helveticus*, *L. casei*, *L. paracasei*, *L. acidophilus,* or *B. lactis*, the acetaldehyde concentration was 5.4, 2.1, 4.5, 2.4, 1.9, and 3.0 ng/mL, respectively, whereas it was not detected in the unfermented juice [19]; (5) in non-pH-adjusted (2.7) sea buckthorn juice, the acetaldehyde concentration was 10.6 ng/mL, which increased after fermentation for 36 and 72 h by *L. plantarum* to 13.9 and 15.8 ng/mL, respectively. However, if the pH of the juice was adjusted to pH 3.5, the initial acetaldehyde concentration of 10.8 ng/mL decreased after *L. plantarum* fermentation for 36 and 72 h to 1.1 and 1.2 ng/mL, respectively [42]; and (6) in watermelon juice fermented by either *L. plantarum* or *P. pentosaceus*, the acetaldehyde concentration was 4.6 and 3.2 ng/mL, respectively, compared to 2.3, 2.0, 0.5, and 0.5 ng/mL in the unfermented juice, and *L. brevis*, *L. casei,* or *L. rhamnosus* fermented juices, respectively [48]. The concentration of acetaldehyde in 6 studies reported here was still well below the concentration that has been reported to adversely affect flavour, indicating that acetaldehyde may have a positive impact on the overall flavour profile of fermented juices if it is above the minimum concentration required for perception. Note, in Chinese wolfberry juice fermented by either *L. plantarum*, *L. casei*, *L. paracasei*, *L. helveticus*, or *B. lactis*, the acetaldehyde concentration was reduced to 52.8, 124.2, 123.3, 23.4, and 13.3 µg/mL, respectively, compared to its concentration in the unfermented juice (155.9 µg/mL), where in the juice fermented by *L. acidophilus*, the acetaldehyde concentration was 188.2 µg/mL [50]. Further, the initial acetaldehyde concentration in a distillate prepared using simple distillation from unfermented melon by-product was 1320 ng/mL, which was reduced to 470 ng/mL in the *L. rhamnosus* fermented melon by-product distillate. Moreover, when using the vacuum distillation method, in the unfermented melon by-product distillate, the acetaldehyde concentration was 160 ng/mL, where it was only 20 ng/mL in the distillate from *L. rhamnosus* fermented melon by-product. Some LAB can convert acetaldehyde to ethanol and acetic acid, which could explain the decrease in acetaldehyde concentration in some fermentations [44].

Another important aldehyde from a flavour perspective as it imparts a pleasant aroma to fermented juices is benzaldehyde, which is generated by LAB from the amino acid phenylalanine. The conversion of phenylalanine to benzaldehyde by LAB is initiated by the aminotransferase enzyme. The resulting phenyl pyruvic acid is chemically converted to benzaldehyde in the presence of oxygen and manganese [58,69]. The benzaldehyde concentration increased after fermentation of vegetable and fruit juices in 7 studies: (1) In Chinese wolfberry juice fermented by either *L. plantarum*, *L. paracasei*, or *L. acidophilus*, the benzaldehyde concentration was 117.2, 68.1, and 40.7 µg/mL, respectively, where it was not detected in the unfermented juice nor in juice fermented by other LAB strains [50]; (2) in kiwifruit juice (Xuxiang and Hongyang cultivars), Xuxiang cultivar juice fermented by *L. acidophilus*, the benzaldehyde concentration was 490.3 ng/mL, compared to 369.7 ng/mL in the unfermented juice. In contrast, it was not detected in the unfermented Hongyang cultivar juice nor in the Xuxiang cultivar juice fermented by *L. helveticus* or *L. plantarum* and all LAB fermented Hongyang cultivar juices [49]; (3) in bog bilberry juice fermented by two *L. plantarum* strains, the benzaldehyde concentration was 55.5 and 62.3 ng/mL, compared to 41.8 ng/mL in the unfermented juice [34]; (4) in non-pH-adjusted (2.7) sea buckthorn juice, the benzaldehyde concentration was 2.7 ng/mL, which increased after fermentation for 36 and 72 h by *L. plantarum* to 5.4 and 7.9 ng/mL, respectively. However, if the pH of the juice was adjusted to pH 3.5, the initial benzaldehyde concentration of 2.3 ng/mL decreased after *L. plantarum* fermentation for 36 and 72 h to 1.1 and 1.7 ng/mL, respectively [42]; (5) in goji juice fermented by different combinations of bacterial strains (either *L. plantarum*, *L. rhamnosus*, *L. reuteri*, *B. velezensis*, or *B. licheniformis*), the benzaldehyde concentration ranged from 55.5 to 101.4 ng/g, compared to 46.3 ng/g in the unfermented juice [25]; (6) in durian pulp fermented by *L. casei* monoculture, the benzaldehyde concentration was 2.9 times higher, compared to its concentration in the sequential co-culture with yeast *W. saturnus*, and it was not detected in the unfermented pulp. This difference is because LAB can convert phenylalanine amino acid to benzaldehyde; however, yeast preferentially convert phenylalanine amino acid to phenylethyl alcohol via the Ehrlich pathway, resulting in a higher quantity of benzaldehyde in LAB fermentations [30]; and (7) in papaya juice fermented by *L. plantarum*, the benzaldehyde concentration was 2 times higher, compared to the concentration after the *L. acidophilus* fermentation or in the unfermented juice [23]. Though the benzaldehyde concentration increased after LAB fermentation, it also reduced in 4 studies: (1) in Hetain jujube juice fermented by LAB, the benzaldehyde concentration ranged from 22.1 to 29.7 µg/mL, compared to 33 µg/mL in the unfermented juice [20]; (2) in jujube pulp fermented by a mixture of *L. plantarum*, *L. rhamnosus*, and *S. thermophilus*, the benzaldehyde concentration was 3516 ng/g, compared to 4672 ng/g in the unfermented pulp [47]; (3) in cherry juice fermented by various *L. plantarum* strains, the benzaldehyde concentration ranged from 15.3 to 33.4 ng/mL, compared to 100.5 ng/mL in the unfermented juice, and in cherry juice fermented by *L. rhamnosus*, the benzaldehyde concentration was 76 ng/mL, compared to 90.5 ng/mL in the unfermented juice [29]; and (4) in okara fermented by LAB monocultures of *L. rhamnosus* or *P. acidilactici* and a co-culture (*L. acidophilus*, *L. rhamnosus*, and *P. acidilactici*), the benzaldehyde concentration was 22.4, 45, and 46.2 µg/g, respectively, compared to 114.6 µg/g in the unfermented okara, and in okara fermented by *L. acidophilus*, the benzaldehyde concentration was only slightly reduced to 109.9 µg/g [46].

Hexanal and nonanal are two other important aldehydes that may influence the flavour profile of fermented juices. Hexanal, which is produced from linoleic fatty acid, imparts a fresh, green, grassy, waxy, fatty, and unpleasant aroma to fermented fruit and vegetable juices [54]. After fermentation, the hexanal concentration was reduced in goji juice [25], mung bean [45], watermelon juice [48], sea buckthorn juice [42], kiwifruit juice [49], and okara pulp [38,46]. In addition, it was not detected after the fermentation of Chinese wolfberry juice [50]. Jin et al. [41] reported that in mango slurry fermented by LAB, the nonanal was detected, where it was not detected after yeast fermentation. Further, the nonanal concentration was reduced in fermented yam juice [33], okara pulp [46], mung bean [45], watermelon juice [48], kiwifruit juice [49], and sea buckthorn juice [42]. It was also not detected during the fermentation of goji juice and durian pulp [25,30].

Overall, the total combined aldehyde concentration was reduced after the fermentation of vegetable and fruit juices in 8 studies. This was probably due to the conversion of aldehydes either by reduction to alcohols or oxidation to acids [23]: (1) In Chinese wolfberry juice fermented by either *L. plantarum*, *L. casei*, *L. paracasei*, *L. acidophilus, L. helveticus,* or *B. lactis*, the total combined aldehyde concentration was 332.8, 680.1, 500.5, 983.3, 422.1, and 492.6 µg/mL, respectively, compared to 1841.2 µg/mL in the unfermented juice [50]; (2) in unfermented jujube (Muzao and Hetain varieties) juice, the total combined aldehyde concentration was 102.8 and 121.3 µg/mL, respectively, compared to 65.4–94.7 and 61.9–83.9 µg/mL in the fermented Muzao, and Hetain varietal juices by different LAB strains, respectively. However, the total combined aldehyde concentration increased by 38.3% in Hetain varietal juice fermented by *L. plantarum* and 158% in Muzao varietal juice fermented by *L. acidophilus* [20]; (3) in kiwifruit juice (Xuxiang and Hongyang cultivars), Xuxiang cultivar juice fermented by either *L. acidophilus*, *L. helveticus*, or *L. plantarum*, the total combined aldehyde concentration was 2411.9, 4248.7, and 4915.8 ng/mL, respectively, compared to 15,316.3 ng/mL in the unfermented juice, where in the Hongyang cultivar juice fermented by either *L. acidophilus*, *L. helveticus*, or *L. plantarum*, the total combined aldehyde concentration was 2466.1, 2528.9, and 3866.4 ng/mL, respectively, compared to 23,477.9 ng/mL in the unfermented juice [49]; (4) in cherry juice fermented by various *L. plantarum* strains, the total combined aldehyde concentrations ranged from 43.5 to 78.2 ng/mL, compared to 208.5 ng/mL in the unfermented juice [29]; (5) in okara fermented by *L. acidophilus*, *P. acidilactici*, or *L. rhamnosus*, and a co-culture (*L. acidophilus*, *P. acidilactici*, and *L. rhamnosus*), the total combined aldehyde concentration was 866.1, 282.9, 25.3, and 68.3 µg/g, respectively, compared to 1450.6 µg/g in the unfermented okara [46]; (6) in watermelon juice fermented by either *L. plantarum*, *L. rhamnosus*, *L. casei*, *L. brevis*, or *P. pentosaceus*, the total combined aldehyde concentration reduced by > 50% compared to the unfermented juice [48]; (7) in grape juice fermented by LAB, the total combined aldehyde concentration reduced by 45.4% [43]; and (8) in tomato juice fermented by either *L. plantarum* or *L. casei*, the total combined aldehyde concentration was 15 and 164 times lower, respectively, compared to the concentration in the unfermented juice [32]. Note, that in apple juice fermented by either *L. plantarum*, *L. helveticus*, *L. casei*, *L. paracasei*, *L. acidophilus*, or *B. lactis*, the total combined aldehyde concentration was reported to have increased to 22.4, 13.3, 7.6, 4.4, 5.4, and 6.0 ng/mL, respectively, compared to 2.5 ng/mL in the unfermented juice [19].

Higher overall combined aldehyde concentrations, particularly if these are dominated by lipid-derived aldehydes, are likely to have a negative impact on the flavour of fermented juices. Reducing the concentrations of lipid-derived aldehydes is likely to increase the fruity aroma of fermented fruits and vegetables while lowering their green odour character. In all of the studies reviewed, most lipid-derived aldehyde compounds were reduced after LAB fermentation (hexanal (8 papers) and nonanal (8 papers)), whereas acetaldehyde (6 papers) and benzaldehyde (7 papers) were increased or were only detected after fermentation and were responsible for the pleasant aroma of fermented fruit and vegetable juices.

### 3.7. Acids

Twenty-five studies out of the thirty-five reported that acids contributed to the flavour of fermented vegetables and fruits. An extensive range of short- to long-chain fatty acids, including acetic acid, propanoic acid (propionic acid), 2-methyl-propanoic acid (isobutyric acid), butanoic acid (butyric acid), 3-methyl-1-butanoic acid (isovaleric acid), 2-methyl-1-butanoic acid, caproic acid (hexanoic acid), enanthic acid (heptanoic acid), caprylic acid (octanoic acid), pelargonic acid (nonanoic acid), capric acid (decanoic acid), lauric acid (dodecanoic acid), palmitic acid (hexadecanoic acid), and oleic acid, were detected in the fermented fruits and vegetables, which were formed from sugars or amino acid catabolism [52].

Acetic acid, which is a key flavour compound of fermented juices, is produced mainly by heterofermentative LAB (*L. brevis*, *Limosilactobacillus fermentum* (*L. fermentum*), *L. reuteri*, *L. plantarum*, *L. rhamnosus*, and *L. casei*), which first utilise sugars via the PK pathway and produce acetyl phosphate, which is subsequently converted into acetic acid by the acetokinase enzyme [13]. However, homofermentative LAB (*L. acidophilus*, *S. thermophilus*, and *L. helveticus*), with high glycolytic flux rates, ferment sugars into solely lactic acid, while under slow growth conditions and low glycolytic flux rates, the homofermentative LAB change to mixed acid fermentation (formic acid, lactic acid, ethanol, and acetic acid) [70]. Acetic acid can also be produced from citrate, which is found in fruit and vegetable juices [65]. The acetic acid concentration increased after fermentation of fruit and vegetable juices in 15 studies: (1) In Chinese wolfberry juice fermented by either *L. casei*, *L. paracasei, L. helveticus,* or *B. lactis*, the acetic acid concentration was 19,773.7, 16,093.9, 12,698.4, and 14,011.1 µg/mL, respectively, and it was not detected in the unfermented juice or juice fermented by *L. plantarum* or *L. acidophilus* [50]; (2) In two varieties of jujube (Muzao and Hetain) juices fermented by different LAB strains, the acetic acid concentration ranged from 161 to 234.3 µg/mL, and 211.2 to 278.1 µg/mL, respectively, compared to 90.8 µg/mL in Muzao and 104.2 µg/mL in Hetain unfermented varietal juices. Interestingly, *L. helveticus* increased the acetic acid concentration by 158.2% in Muzao varietal juice and *L. casei* increased it by 166.9% in Hetian varietal juice [20]; (3) in elderberry juice fermented by either *L. plantarum*, *L. casei,* or *L. rhamnosus* strains, the acetic acid concentration ranged from 205.9–1012.3, 62.3–122.1, and 47.2–132.1 ng/mL, respectively, compared to 0.3–12.2 ng/mL in the unfermented juice [22]; (4) in cherry juice fermented by various *L. plantarum* strains, the acetic acid concentration ranged from 54.8 to 184.8 ng/mL, compared to 0.01 ng/mL in the unfermented juice [29]; (5) in non-pH-adjusted (2.7) sea buckthorn juice, the acetic acid concentration was 1.0 ng/mL which increased after fermentation for 36 and 72 h by *L. plantarum* to 2.1 and 3.5 ng/mL, respectively, where the pH of the juice was adjusted to pH 3.5, the initial acetic acid concentration of 0.8 ng/mL increased after fermentation with *L. plantarum* for 36 and 72 h to 50.5 and 85.9 ng/mL, respectively [42], 6. In grape juice fermented by LAB, the acetic acid concentration was 25.5 ng/mL, and it was not detected in the unfermented juice [43]; (7) in jujube juice fermented by a mixture of *L. plantarum*, *L. rhamnosus*, and *S. thermophilus*, the acetic acid concentration was 12.2 µg/g, compared to 2.8 µg/g in the unfermented juice [47]; (8) in horse gram sprouts fermented by *L. plantarum* strains, the acetic acid concentration ranged from 4.8 to 5 µg/g, and it was not detected in the raw seed [37]; (9) in goji juice fermented by different combinations of bacterial strains (either *L. plantarum*, *L. rhamnosus*, *L. reuteri*, *B. velezensis*, or *B. licheniformis*), the acetic acid concentration ranged from 25.4 to 88.9 ng/g, where it was not detected in the unfermented juice [25]; (10) in mung bean fermented by *L. plantarum* strains, the acetic acid concentration ranged from 0.17 to 0.29 ng/g, where it was not detected in the unfermented mung bean [45]; (11) in papaya juice fermented by either *L. acidophilus* or *L. plantarum*, the acetic acid concentration was 5.7 and 2.4 times higher, respectively, compared to the concentration in the unfermented juice [23]; (12) in mango slurry fermented by either *L. plantarum* or *S. thermophilus*, the acetic acid concentration was 2.4 and 2.7 times higher, respectively, compared to the concentration in the unfermented mango slurry, and in addition, it was not detected in *L. casei* or yeast fermentations [41]; (13) in tomato juice fermented by *L. plantarum,* the acetic acid concentration was 8.6 times higher compared to the concentration in the unfermented juice, where it was not detected in *L. casei* fermented juice [32]; (14) in yam juice fermented by LAB, the acetic acid concentration was 1.5 times higher compared to the concentration in the unfermented yam juice [33], and 15. in durian pulp fermented by *L. casei*, the acetic acid concentration was 1.4 times higher compared to the concentration in a co-culture of *L. casei* and yeast *W. saturnus*, where it was not detected in the unfermented pulp [30].

3-Methyl-1-butanoic acid is an important acid flavour compound in dairy foods, which is produced from the amino acid leucine by an aminotransferase enzyme [57]. However, when present at high concentrations, it may negatively impact on the flavour of fermented juices [26]. The 3-methyl-1-butanoic acid concentration increased in 3 studies after LAB fermentation: (1) In non-pH-adjusted (2.7) sea buckthorn juice, the 3-methyl-1-butanoic acid concentration was 19.2 ng/mL, which increased after fermentation for 36 and 72 h by *L. plantarum* to 25 and 39 ng/mL, respectively, where the pH of the juice was adjusted to pH 3.5, the initial 3-methyl-1-butanoic acid concentration of 13.1 ng/mL increased after *L. plantarum* fermentation for 36 and 72 h to 129.3 and 185 ng/mL, respectively [42]; (2) in tomato juice fermented by *L. plantarum*, the 3-methyl-1-butanoic acid concentration was 3 times higher compared to the concentration in the unfermented juice, where, it was not detected in *L. casei* fermented juice [32]; and (3) in papaya juice fermented by *L. plantarum*, the 3-methyl-1-butanoic acid concentration was 4.1 times higher compared to the concentration in *L. acidophilus* fermented juice, where it was not detected in the unfermented juice [23].

Butanoic acid, which is produced from the fermentation of sugars through the fatty acid biosynthesis pathway [71] and can confer a dairy/cheesy aroma to fermented juices, was detected in 3 studies after fermentation: (1) In jujube juice fermented by a mixture of *L. plantarum*, *L. rhamnosus*, and *S. thermophilus*, the butanoic acid concentration was 1487 ng/g, compared to 345 ng/g in the unfermented juice [47]; (2) in apple juice fermented by different LAB, the butanoic acid concentration ranged from 2.3 to 4.7 ng/g, where it was not detected in the unfermented juice [21]; and (3) in okara fermented by a combination of *R. oligosporus* and *Y. lipolytica*, the butanoic acid concentration was 34 times higher compared to the concentration in the juice fermented by *R. oligosporus* monoculture [38]. However, in mango slurry fermented by the yeast *S. cerevisiae*, the butanoic acid concentration was 4.4–5.6 times lower compared to the concentration in the unfermented mango slurry, where the butanoic acid concentration was slightly higher after LAB fermentation compared to the unfermented mango slurry [41].

Hexanoic acid, which can be produced from the fermentation of sugars through the fatty acid biosynthesis pathway or from the cleavage of linoleic acid via hexanal, may impart a fatty/cheesy/sour flavour to fermented juices [22,54,72,73]. Hexanoic acid was detected after fermentation in 4 studies: (1) In jujube juice fermented by a mixture of *L. plantarum*, *L. rhamnosus*, and *S. thermophilus*, the hexanoic acid concentration was 15.1 µg/g, compared to 7.1 µg/g in the unfermented juice [47]; (2) in non-pH-adjusted (2.65) bog bilberry juice fermented by two strains of *L. plantarum*, the hexanoic acid concentration was 266 and 272.1 ng/mL, compared to 147 ng/mL in the unfermented juice. However, if the pH of the juice was adjusted to pH 3.5, the initial hexanoic acid concentration of 132.6 ng/mL was slightly changed after fermented by two strains of *L. plantarum* to 136.9 and 123.2 ng/mL [34]; (3) in grape juice fermented by LAB, the hexanoic acid concentration was 14.2 ng/mL, where it was not detected in the unfermented juice [43]; and (4) in mango slurry fermented by different LAB, the hexanoic acid concentration was 1.4–1.7 times higher than the concentration in the unfermented slurry. However, in yeast fermentation, the hexanoic acid concentration was 1.2–1.6 times lower compared to the concentration in the unfermented juice [41]. According to Li et al. [20], in Muzao varietal jujube juice, the initial hexanoic acid concentration of 69 µg/mL was reduced after fermentation by different LAB to between 9.2 and 54.5 µg/mL, with the highest reduction being reported for *L. acidophilus* fermented juice. In the same study, in Hetain varietal jujube juice, the initial hexanoic acid concentration was 19.0 µg/mL, which increased after fermentation by different LAB to 31.2–75.9 µg/mL, with the highest concentration being obtained in *L. acidophilus* fermented juice.

Octanoic acid, which is synthesised from sugars (glucose) through the fatty acid biosynthesis pathway [74], was detected in 6 studies after the fermentation of juices: (1) In non-pH-adjusted (2.65) bog bilberry juice fermented by two strains of *L. plantarum*, the octanoic acid concentration was 3921.6 and 4013.2 ng/mL, compared to 1538.2 ng/mL in the unfermented juice. However, if the pH of the juice was adjusted to pH 3.5, the initial octanoic acid concentration of 1156.5 ng/mL was reduced after fermentation by two strains of *L. plantarum* to 655.3 and 722.9 ng/mL [34]. (2) in goji juice fermented by a bacterial mixture, the octanoic acid concentration ranged from 251.8 to 321.5 ng/g, and it was not detected in the unfermented juice [25]; (3) in grape juice fermented by LAB, the octanoic acid concentration was 5.7 ng/mL, and it was not detected in the unfermented juice [43]; (4) in yam juice fermented by LAB, the octanoic acid concentration was 2 times higher compared to the concentration in the unfermented juice [33]; (5) in durian pulp fermented by *L. casei*, the octanoic acid concentration was 2.3 times higher compared to the concentration in the unfermented pulp, and when the pulp was fermented by *L. casei* combined with yeast *W. saturnus*, the octanoic acid concentration was 2 times lower compared to the concentration in the unfermented pulp [30]; and (6) in mango slurry, the octanoic acid was detected after fermentation by the yeast *S. cerevisiae*, but it was not detected after LAB fermentation [41].

Chen et al. [33] reported that *L. plantarum* alone or in combination with *S. thermophilus* increased acetic, nonanoic, and decanoic acids in fermented yam juice, reducing the astringent odour of the fermented juice.

Overall, volatile acids after LAB fermentation made contributions to the flavour of fruit and vegetable juices, where acetic acid (15 papers) was the most commonly detected volatile acid present in fermented juices, followed by octanoic acid (6 papers), hexanoic acid (4 papers), 3-methyl-1-butanoic acid (3 papers), and butanoic acid (3 papers).

### 3.8. Terpenes and Norisoprenoids

Terpenes, which are comprised of isoprene (C5) units, impart floral, rose, and fruity flavours. In 13 studies reported here, terpenes were classified as being terpenes or norisoprenoids; interestingly, in 14 studies, some terpene compounds (β-linalool, α-terpineol, β-citronellol, geraniol, β-damascenone, D-limonene, and trans-β-ionone) were classified as either alcohol/hydrocarbon/ketone, or as alkenes in 2 studies. Terpenes are classified based on the number of isoprene units they contain: hemiterpenoids (C5), monoterpenoids (C10), sesquiterpenoids (C15), diterpenoids (C20), sesterterpenoids (C25), triterpenoids (C30), tetraterpenoids (C40) (β-carotene), and polyterpenoids (C > 40) [12,75]. β-carotene can be further oxidized into β-ionone, β-damascenone, and β-ionol. Bacteria biosynthesize terpenoids via the 2-C-methyl-D-erythritol-4-phosphate (MEP) pathway [76], while yeast produce them via the mevalonate pathway [75]. (β)-Myrcene, D-limonene (1-Methyl-4-(prop-1-en-2-yl) cyclohex-1-ene), ocimene, (β)-linalool (3,7-Dimethyl-1,6-octadien-3-ol), camphene, p-cymene, α-terpinolene, (α)-terpinene, 1,8-cineole, (ɤ)-terpinene, (α)-terpineol (2-(4-Methylcyclohex-3-en-1-yl)propan-2-ol), Citronellyl formate, (β)-damascenone ((E)-1-(2,6,6-Trimethyl-1-cyclohexa-1,3-dienyl) but-2-en-1-one), (β)-citronellol (3,7-Dimethyloct-6-en-1-ol), geraniol ((2E)-3,7-Dimethylocta-2,6-dien-1-ol), trans-(β)-ionone (4-(2,6,6-Trimethylcyclohex-1-en-1-yl) but-3-en-2-one), m-cymene, prenol, (α)-ionene, D-germacrene, valencene, cedrol, phytol, myrtenol, eugenol (2-Methoxy-4-(prop-2-en-1-yl) phenol), and β- phellandrene were the major terpene compounds detected in the reviewed papers.

Linalool, an important flavour terpene, was detected after fermentation of juices in 7 studies: (1) The linalool concentration of a distillate prepared using simple distillation from unfermented orange pomace was 1.6 µg/mL and after fermentation by *L. rhamnosus* of the orange pomace, the concentration in the resulting distillate was 2.3 µg/mL. Similarly, in distillates prepared using vacuum distillation, the linalool concentration in the unfermented orange pomace distillate was lower than that in the distillate from *L. rhamnosus* fermented orange pomace, at 0.1 µg/mL and 1.1 µg/mL, respectively. In the same study, in the unfermented melon by-product distillate prepared using simple distillation, the linalool concentration was 0.5 µg/mL and in the *L. rhamnosus* fermented melon by-product distillate, the concentration was 4.4 µg/mL. However, linalool was not detected in the distillates of fermented and unfermented melon by-products prepared using vacuum distillation [44]; (2) in elderberry juice fermented by either *L. plantarum* or *L. rhamnosus*, the linalool concentration was 328.5 and 339.7 ng/mL, respectively, compared to 79.3 and 189.3 ng/mL in the unfermented juices, respectively [22]; (3) in cherry juice fermented by either *L. rhamnosus*, *L. paracasei,* or *L. plantarum*, the linalool concentration was 39.6, 21.5, and 19.6–29 ng/mL, respectively, compared to 13.7–15.7 ng/mL in the unfermented juice [29]; (4) in goji juice fermented by a bacterial mixture (*L. plantarum*, *L. rhamnosus*, *B. velezensis*, and *B. licheniformis*), the linalool concentration was 22.3 ng/g, where it was not detected in the unfermented juice [25]; (5) in apple juice fermented by different LAB, the linalool concentration ranged from 3.0 to 4.5 ng/g, compared to 2.0 ng/g in the unfermented juice [21]; (6) in yam juice fermented by *L. plantarum*, the linalool concentration was 21 times higher compared to the concentration in the juice fermented by a combination of *L. plantarum* and *S. thermophilus*, and it was not detected in the unfermented yam juice [33]; and (7) in mango slurry fermented by LAB, linalool was detected, where it was not detected in yeast fermented or unfermented slurry [41]. Though the linalool concentration increased in these studies after fermentation, it was also reduced after fermentation in 2 studies: (1) In papaya juice fermented by either *L. acidophilus* or *L. plantarum*, the linalool concentration was 1.6 and 1.2 times lower, respectively, compared to the concentration in the unfermented juice [23]; and (2) in *Momordica charantia* juice fermented by *L. plantarum*, the linalool concentration was reduced compared to the concentration in the unfermented juice [18].

D-limonene, a significant flavour terpene compound, increased in 8 experiments after the fermentation of juices: (1) In Chinese wolfberry juice fermented by either *L. casei*, *L. paracasei, L. acidophilus*, *L. helveticus,* or *B. lactis*, the D-limonene concentration was 281.4, 218, 372.6, 271.8, and 273.5 µg/mL, respectively, compared to 143.5 µg/mL in the unfermented juice, where in fermented juice by *L. plantarum*, the D-limonene concentration was 67.4 µg/mL [50]; (2) the D-limonene concentration in distillates prepared using vacuum distillation from *L. rhamnosus* fermented melon by-product or unfermented melon by-product was 140 ng/mL and 10 ng/mL, respectively. In the same study, distillates prepared using vacuum distillation had concentrations of 100 ng/mL and 20 ng/mL in the *L. rhamnosus* fermented orange pomace distillate and the unfermented pomace distillate, respectively [44]; (3) in cherry juice fermented by either *L. rhamnosus* or *L. paracasei*, the D-limonene concentration was 7.9 and 3.0 ng/mL, respectively, compared to 2.8 ng/mL in the unfermented juice, where in cherry juice fermented by four *L. plantarum* strains, the D-limonene concentration was 1.8, 1.9, 3.1, and 4.0 ng/mL, compared to 2.3 ng/mL in the unfermented juice [29]; (4) in jujube juice fermented by a mixture of *L. plantarum*, *L. rhamnosus*, and *S. thermophilus*, the D-limonene concentration was 964 ng/g, where it was not detected in the unfermented juice [47]; (5) in apple juice fermented by different LAB, the D-limonene concentration ranged from 2.5 to 3.1 ng/g, compared to 1.1 ng/g in the unfermented juice [21]; (6) in mango slurry fermented by different LAB, the D-limonene concentration was 7.8–10 times higher compared to the concentration in the unfermented slurry, where in yeast fermentation, the D-limonene concentration was 2.2 to 4.5 times lower compared to the concentration in the unfermented juice [41]; (7) in pomegranate juice fermented by *L. plantarum* strains, the D-limonene concentration was increased compared to the concentration in the unfermented juice [24]; and (8) in tomato juice fermented by *L. casei*, D-limonene was detected, where it was not detected after fermentation by *L. plantarum* or in the unfermented juice [32]. However, the D-limonene concentration was reduced in 2 studies after fermentation: (1) The D-limonene concentration in a distillate prepared using simple distillation from *L. rhamnosus* fermented orange pomace was 2.3 µg/mL, compared to 4.8 µg/mL in the unfermented pomace distillate [44]; and (2) in blended apple, orange, carrot, and jujube juices fermented by a mixed starter culture (*L. plantarum*, *B. breve*, and *S. thermophilus*), the D-limonene concentration was 170 ng/mL, compared to 2093 ng/mL in the unfermented blended juice [35].

In 5 studies, α-terpineol was detected after LAB fermentation: (1) The α-terpineol concentration in a distillate prepared using vacuum distillation from *L. rhamnosus* fermented orange pomace was 1.5 µg/mL, compared to 0.3 µg/mL in the unfermented pomace distillate, where it was not detected in distillates prepared using simple distillation. In the same study, using simple distillation, in a distillate of melon by-product fermented by *L. rhamnosus*, the α-terpineol concentration was 2.0 µg/mL, compared to 1.0 µg/mL in the unfermented melon by-product distillate. Whereas using vacuum distillation, the α-terpineol concentration was slightly increased to 0.25 µg/mL in the distillate from the fermented melon by-product, compared to 0.18 µg/mL in the unfermented melon by-product distillate [44]; (2) in kiwifruit juice (Hongyang and Xuxiang cultivars), Hongyang cultivar juice fermented by either *L. acidophilus*, *L. helveticus*, or *L. plantarum*, the α-terpineol concentration was 84.2, 154.9, and 151.1 ng/mL, respectively, where it was not detected in the unfermented juice, and in addition, the α-terpineol was not detected in the unfermented and the fermented Xuxiang cultivar juices [49]; (3) in goji juice fermented by a bacterial mixture (*L. rhamnosus*, *L. reuteri*, and *B. velezensis*), the α-terpineol concentration was 38.7 ng/g, compared to 33.9 ng/g in the unfermented juice [25]; (4) in non-pH-adjusted (2.65) bog bilberry juice fermented by two strains of *L. plantarum*, the α-terpineol concentration was 5.2 and 5.9 ng/mL, compared to 4.3 ng/mL in the unfermented juice, where the pH of the juice was adjusted to pH 3.5, the initial α-terpineol concentration was 3 ng/mL, which was reduced after fermentation by two strains of *L. plantarum* to 2.8 and 2.5 ng/mL [34]; and (5) in mango slurry fermented by different LAB, α-terpineol was detected, where it was not detected after yeast fermentation or in the unfermented mango slurry [41].

β-damascenone, another important flavour terpene compound, was detected after fermentation in 4 studies: (1) In Hetain varietal jujube juice fermented by either *L. acidophilus*, *L. casei*, *L. helveticus*, or *L. plantarum*, the β-damascenone concentration was 16.7, 22.4, 19.0, and 21.4 µg/mL, respectively, where it was not detected in the unfermented juice [20]; (2) the β-damascenone concentration in a distillate prepared using simple distillation from a melon by-product fermented by *L. rhamnosus* was 8 µg/mL, compared to 1.2 µg/mL in the unfermented melon by-product distillate, and when using vacuum distillation, the β-damascenone concentration was 0.03 µg/mL in unfermented melon by-product distillate and 0.12 µg/mL in the distillate of *L. rhamnosus* fermented melon by-product [44]; (3) in goji juice fermented by different combinations of mixed bacterial cultures, the β-damascenone concentration ranged from 39.6 to 52 ng/g, where it was not detected in the unfermented juice [25]; and (4) in apple juice fermented by different LAB, the β-damascenone concentration ranged from 2.4 to 3.9 ng/mL, where it was not detected in the unfermented juice [19]. However, Chen et al. [21] reported in apple juice that the initial β-damascenone concentration was 12.8 ng/g and that after fermentation with LAB, it was not detected.

Eugenol is a volatile phenol that imparts clove, honey, and spicy notes to fermented fruit and vegetable juices. In the three papers, where it was mentioned, it was classified as a terpene: (1) In horse gram sprouts fermented by two *L. plantarum* strains, the eugenol concentration was 5.2 and 5.3 µg/g, and it was not detected in raw seeds [37]; (2) in cherry juice fermented by either *L. paracasei* or *L. rhamnosus*, the eugenol concentration was 3.9, and 26.8 ng/mL, respectively, compared to 2.4 ng/mL in the unfermented juice, and cherry juice fermented by four *L. plantarum* strains, the eugenol concentration was 11.1, 13.0, 14.7, and 22.4 ng/mL, compared to 3.2 ng/mL in the unfermented juice [29]; and (3) in apple juice fermented by either *L. plantarum*, *L. rhamnosus*, *L. casei*, or *L. acidophilus*, the eugenol concentration was 9.1, 6.2, 17.1, and 33.3 ng/g, respectively, compared to 5.4 ng/g in the unfermented juice [21].

In addition, geraniol was detected in 3 studies after fermentation: (1) In watermelon juice fermented by either *L. plantarum*, *L. brevis*, *P. pentosaceus, L. casei,* or *L. rhamnosus*, the geraniol concentration was 5.6, 1.7, 1.7, 1.5, and 1.3 ng/mL, respectively, compared to 1.0 ng/mL in the unfermented juice [48]; (2) in goji juice fermented by bacterial mixture, the geraniol concentration ranged from 119.3–246 ng/g, where it was not detected in the unfermented juice [25]; and (3) in apple juice fermented by either *L. plantarum*, *L. rhamnosus*, *L. casei,* or *L. acidophilus*, the geraniol concentration was 15.9, 13.2, 14.4, and 14.3 ng/g, respectively, compared to 5.5 ng/g in the unfermented juice [21].

Overall, a wide range of terpenes were found to increase after LAB fermentation of fruit and vegetable juices, with D-limonene being the most commonly reported (8 papers) terpene, followed by linalool (7 papers), α-terpineol (5 papers), β-damascenone (4 papers), eugenol (3 papers), and geraniol (3 papers). However, in some instances after fermentation, the concentration of linalool (2 papers), D-limonene (2 papers), and β-damascenone (1 paper) decreased.

### 3.9. Phenols

Nine out of thirty-five studies detected phenolic compounds, which were categorised into phenols or alcohols or others. Phenol, 4-vinylphenol-2-methoxy, 2,6-di-tert-butyl-4-methylphenol, 4-ethyl-2-methoxyphenol, 4-ethyl-phenol, 2-methoxy-phenol, 2,5-dimethyl-phenol, 2,4,5-trimethyl-phenol, and 2,6-dimethoxyphenol were the phenolic compounds most frequently reported. The concentration of 2-methoxy-4-vinylphenol increased after fermentation in 2 studies: (1) In goji juice fermented by a mixed bacterial culture, the 2-methoxy-4-vinylphenol concentration ranged from 532.5 to 678.6 ng/g, compared to 98.3 ng/g in the unfermented juice [25]; and (2) in apple juice fermented by either *L. plantarum*, *L. rhamnosus*, *L. casei,* or *L. acidophilus*, the 2-methoxy-4-vinylphenol concentration was 2.4, 1.7, 2.1, and 2.1 ng/g, respectively, compared to 0.2 ng/g in the unfermented juice [21]. In addition, the concentration of phenol increased after fermentation in 3 studies: (1) In apple juice fermented by either *L. plantarum* or *L. acidophilus*, the phenol concentration was 3.2 and 2.2 ng/g, respectively, compared to 0.1 ng/g in the unfermented juice [21]; (2) in papaya juice fermented by *L. acidophilus*, the phenol concentration was 1.6 times higher compared to the concentration in *L. plantarum* fermented juice, where it was not detected in the unfermented juice [23]; and (3) in okara fermented by the fungi *R. oligosporus* in combination with the yeast *Y. lipolytica*, the phenol concentration was 2.4 times higher compared to the concentration when fermented by *R. oligosporus* in monoculture [38].

### 3.10. Furans

Furfural, 2-ethyl-furan, 2-propyl-furan, 2-pentyl-furan, 2,5-dimethyl-furan, 2,4-dimethyl-furan, trans-2-(2-pentyl) furan, 2,3-Dihydrobenzofuran, and acetyl-furan were the major furans identified in 13 studies under furans/aldehydes/others/heterocyclic compounds. Furfural was detected in 8 studies, where it was mainly classified as an aldehyde. Production of furfural is linked to Maillard reactions, and higher levels of furfural may have a negative impact on the flavour of fermented substrates. However, LAB fermentation reduces the amount of furfural, most likely as a result of the consumption of precursors such as amino acids and reducing sugars [24,77,78]. The furfural concentration was reduced after LAB fermentation in 4 studies: (1) In jujube juice fermented by a mixture of *L. plantarum*, *L. rhamnosus*, and *S. thermophilus*, the furfural concentration was 1886 ng/g, compared to 3873 ng/g in the unfermented juice [47]; (2) in cherry juice fermented by four different *L. plantarum* strains, the furfural concentration was 26.1, 36.8, 43.5, and 51.6 ng/mL, compared to 101.7 ng/mL in the unfermented juice [29]; (3) in apple juice fermented by either *L. rhamnosus*, *L. casei,* or *L. acidophilus*, the furfural concentration was 95.1, 106.9, and 91.8 ng/g, respectively, compared to 114.2 ng/g in the unfermented juice [21]; and (4) in pomegranate juice fermented by different *L. plantarum* strains, the furfural concentration was reduced compared to the concentration in the unfermented juice [24]. However, in jujube (Muzao and Hetain varieties) juice, the initial furfural concentration in the Muzao varietal juice was 2.6 µg/mL, which was increased after fermentation by different LAB to a range of 2.9–5.2 µg/mL. In the same study, in Hetain varietal juice, the initial furfural concentration of 4.2 µg/mL was increased after fermentation by LAB to a range of 5.1–5.8 µg/mL [20]. Further, in goji juice fermented by a mixed bacterial culture, the 2-pentyl furan concentration ranged from 233.8 to 422.5 ng/g, compared to 36.3 ng/g in the unfermented juice [25], and in watermelon juice fermented by either *L. plantarum*, *L. brevis*, *P. pentosaceus, L. casei,* or *L. rhamnosus*, the 2-pentyl furan concentration was 96.7, 115, 117, 112, and 99 ng/mL, respectively, compared to 79.4 ng/mL in the unfermented juice [48]. However, the concentration of 2-pentyl furan was reduced in another 2 studies [18,38].

### 3.11. Sulphur Compounds

Seven studies found that sulphur compounds were present in fermented juices, and the major compounds detected were methanethiol, dimethyl sulfide, dimethyl disulfide, dimethyl trisulfide, methyl isopentyl disulfide, methyl ethyl disulphide, diethyl disulphide, 3,5-dimethyl, 1,2,4-Trithiolane (isomer 1), 3,5-dimethyl, 1,2,4-Trithiolane (isomer 2), methyl propyl sulfide, and methyl (methylthio) methyl disulfide. Sulphur compounds such as methanethiol, dimethyl sulfide, and dimethyl disulfide, depending upon concentration, have been found to cause off-flavour in fruit and vegetable substrates [79]. Of the seven studies, two reported that the concentration of sulphur compounds was reduced after fermentation: (1) In durian pulp fermented by either *L. casei* monoculture or *L casei* co-culture with the yeast *W. saturnus*, the diethyl disulfide concentration was 6.9 and 8.9 times lower, respectively, compared to the concentration in the unfermented pulp [30]; and (2) in pomegranate juice fermented by different *L. plantarum* strains, the methanethiol, dimethyl sulfide, and dimethyl disulfide concentrations were reduced compared to the concentration in the unfermented juice [24]. However, in non-pH-adjusted (2.7) sea buckthorn juice, the dimethyl disulfide concentration was 2.1 ng/mL, which was increased after fermentation for 36 and 72 h by *L. plantarum* to 12.2 and 10.9 ng/mL, respectively [42], and in watermelon juice fermented by either *L. plantarum*, *L. brevis*, *P. pentosaceus, L. casei,* or *L. rhamnosus*, the dimethyl disulfide concentration was 6.4, 3.4, 4.1, 5.8, and 9.6 ng/mL, respectively, compared to 1.1 ng/mL in the unfermented juice [48].

### 3.12. Alkanes, Alkenes, and Benzene Derivatives

Alkanes, alkenes, and benzene derivatives were reported less frequently, with only eight of thirty-five studies mentioning them under alkanes/alkenes/benzene derivative/hydrocarbons/others.

#### 3.12.1. Alkanes

In 5 studies, the alkanes, butane, pentane, heptane, octane, nonane, decane, undecane, dodecane, 6,10,14-tetramethyl-hexadecane, 2,6,10,14-tetramethyl-pentadecane, 4-methyloctane, and 2,4-dimethylheptane were detected after fermentation [24,41,42,46,48].

#### 3.12.2. Alkenes

After fermentation, the alkenes 2-methyl-1-propene, 2-methyl-1,3-butadiene, and 3,4-dimethyl octene were reported in 3 studies [22,24,44].

#### 3.12.3. Benzene Derivatives

Benzene, methyl benzene, 1,3,5-trimethyl benzene, naphthalene, p-methyl ethenyl toluene, ethyl benzene, styrene, 1,3-bis(1,1-dimethylethyl) benzene, and 1-1-6-trimethyl-1,2-dihydronaphthalene (TDN) were detected in two studies after fermentation [24,29].

## 4. Summary

Overall, a large array of VOCs was detected after the fermentation of fruit and vegetable juices. The major VOCs identified were ethanol, octanol, hexanol, benzyl alcohol, 3-methyl-1-butanol, and 2-phenylethyl alcohol (alcohols); ethyl acetate, 2-phenylethyl acetate, and ethyl butanoate (esters); acetoin, and diacetyl (ketones); acetaldehyde, and benzaldehyde (aldehydes); acetic acid, 3-methyl-1-butanoic acid, hexanoic acid, butanoic acid, and octanoic acid (acids); linalool, D-limonene, β-damascenone, α-terpineol, eugenol, and geraniol (terpenes and norisoprenoids).

The use of different fruit and vegetable substrates, micro-organisms, and fermentation conditions are all likely to have had an impact on the production of fermentation VOCs. With the exception of a few studies that used bacteria other than LAB, fungi, or yeast either as a monoculture or in combination with LAB, most studies used LAB either as a monoculture or in mixed cultures. The LAB most frequently used for producing desirable VOCs were *L. plantarum*, *L. casei*, *L. acidophilus*, *L. rhamnosus*, and *L. helveticus*. In studies that used two cultivars of a fruit, there were notable differences in the resulting fermented VOCs present and their concentrations [20,49]. Additionally, there were variations in the types and concentrations of VOCs between the pH-adjusted and non-adjusted juices of sea buckthorn [42] and bog bilberry [34]. Moreover, there were observable differences in the detected VOCs from distillates (simple or vacuum distillation) of LAB-fermented orange pomace and melon by-product, assessed by SPME [44]. The results demonstrate how VOCs detected after being produced during fermentation are greatly influenced by the substrate (species and cultivar) being fermented, the LAB strain being used, and the fermentation conditions.

Overall, in LAB-fermented fruit and vegetable juices, the concentrations of the main dairy flavour VOCs, namely acetoin and diacetyl, ranged between 0.04 and 528.1 µg/mL, and 0.01 and 71.9 µg/mL, respectively. It was apparent that LAB fermentation can yield high concentrations of acetoin and diacetyl from plant-based substrates. However, a wide variety of VOCs, including desirable and undesirable compounds, were detected in all of the studies reviewed. Once desirable dairy flavour components have been produced, extracting and purifying them from the other components present will be the next challenge. Current research is focusing on metabolic engineering techniques that involve overexpressing rate-limiting enzymes that produce desirable VOCs or inactivating the enzymes that produce undesirable VOCs in order to improve or create new metabolic pathways in micro-organisms. For instance, pyruvate is a crucial intermediate in the synthesis of the dairy flavours acetoin and diacetyl. Pyruvate in excess can be converted to α-acetolactate by modifying the metabolic flux of pyruvate. If acetoin production is of particular interest, α-acetolactate decarboxylase can be designed to be overexpressed, whereas when diacetyl production is of interest, NADH-oxidase can be overexpressed and α-acetolactate decarboxylase expression can be inactivated [66] (see acetoin/diacetyl production pathway in the ketones section/Figure 3). However, due to the complexity of plant matrices, the action of different metabolic processes, and the factors influencing the fermentation, such as temperature, pH, and aeration, desirable VOCs might be metabolised, or their presence masked by undesirable VOCs. To meet these challenges, research on the metabolic pathway analysis of various micro-organism(s) on complex matrices of plants is required.

## 5. Conclusions

In conclusion, differences in substrates, micro-organisms, and fermentation conditions influence the synthesis of microbial VOCs from vegetable and fruit substrates. In comparison to other bacteria, yeast, and fungi examined, LAB strains were most frequently used to ferment fruit and vegetable substrates. Among LAB strains, *Lactiplantibacillus plantarum* was the most frequently used species and it produced the highest concentration of VOCs. The most frequently used fermentation temperature and time combination was 37 °C for 48 h; however, in the papers reviewed, most of the papers used temperatures of 30 and 37 °C for time combinations ranging from 20 to 120 h. Acids, alcohols, aldehydes, esters, ketones, and terpenes/norisoprenoids were the most frequent VOCs reported after the fermentation of vegetable and fruit substrates, whereas sulphur compounds, phenols, furans, alkanes, alkenes, and benzene derivatives were reported less frequently. After LAB fermentation, the concentration of alcohols, esters, ketones, acids, and terpenes/norisoprenoids generally increased, whereas the concentration of aldehydes generally reduced. The fermentation of vegetable and fruit substrates by different LAB strains generates a wide range of desired VOCs, including the dairy flavours of acetoin and diacetyl. However, due to the complexity of plant matrices, fermenting conditions, and different LAB and their metabolic characteristics, producing dairy analogues with characteristic dairy flavours is still difficult. To achieve the dairy flavours of interest for dairy analogues, in-depth research is still required on the metabolic characteristics and pathways of LAB.

## Figures and Tables

**Table 1:** Inclusion and exclusion criteria for screening papers.

Inclusion Criteria	Exclusion Criteria
Original research papers published in recognized journals	Papers published in predatory journals
Fermented flavours of fruit and vegetable substrates	Fermented flavours of wine, beer, cocoa, meat, and milk-based substrates Plant juices added in milk substrates

**Table 2:** Overview of the study conditions used in the 35 papers that met the inclusion criteria on the VOCs associated with fermented vegetable and fruit substrates.

No	Substrate	Micro-organisms	Fermentation Conditions	Extraction and Analysis of Flavour Volatiles	Major Flavour Volatile Classes	Reference
1	Apple, carrot, tomato, cucumber, and haw mixed juice (40: 25: 15: 15: 5)	*L. rhamnosus* *L. plantarum* *L. casei* *L. acidophilus* *L. fermentum*	Inoculum: 2% (*v*/*v*) at 30 °C for 24 h	Extracted and analysed using HS-SPME-GC-MS	Acids (1) Alcohols (2) Aldehydes (3) Esters (2) Ketones (2)	[17]
2	*Momordica charantia* L. juice	*L. plantarum*	Inoculum: Strain powder 0.01% (*w*/*v*) (11.0 log CFU/mL) at 37 °C for 48 h	Extracted and analysed using HS-SPME-GC-MS	Acids (4) Alcohols (20) Aldehydes (17) Esters (3) Heterocyclic (2) Hydrocarbons (4) Ketones (8) Terpenes and norisoprenoids (13) Others (2)	[18]
3	Apple juice (Fuji apple (*Malus pumila* Mill.))	*L. plantarum* *L. helveticus* *L. casei* *L. paracasei* *L. acidophilus* *B. lactis*	Inoculum: 1% (*v*/*v*) (8.0 log CFU/mL) at 37 °C for 48 h pH adjusted to 5.0 using food-grade Na_2_CO_3_	Extracted and analysed using HS-SPME-GC-MS	Alcohols (13) Aldehydes (5) Esters (24) Ketones (10)	[19]
4	Jujube juice (Two varieties *Ziziphus Jujuba cv. Muzao* and *Hetian*)	*L. acidophilus* *L. casei* *L. helveticus* *L. plantarum*	Inoculum: 0.5% (*v*/*v*) at 37 °C for 48 h pH adjusted to 5.0 using food-grade Na_2_CO_3_ and brix adjusted to 10.0 °Brix with potable water	Extracted and analysed using HS-SPME-GC-MS	Acids (14) Alcohols (10) Aldehydes (19) Esters (8) Ketones (12)	[20]
5	Apple juice	*L. acidophilus* *L. rhamnosus* *L. casei* *L. plantarum*	Inoculum: 7.0 log CFU/mL pH adjusted to 6.0 using food grade Na_2_CO_3_ at 37 °C for 80 h	Aroma profile analysed by electronic nose system and characterized by HS-SPME/GC-MS.	Acids (2) Alcohols (16) Aldehydes (5) Esters (12) Ketones (6) Phenols (4) Terpenes and norisoprenoids (6)	[21]
6	Elderberry juice (*Sambucus nigra* L.)	*L. plantarum* *L. rhamnosus* *L. casei*	Inoculum: 7.0 log CFU/mL at 30 °C (*L. plantarum*), and 37 °C (*L. rhamnosus* and *L. casei*) for 48 h	Extracted and analysed using HS-SPME-GC-MS	Acids (6) Alcohols (19) Aldehydes (3) Alkenes (3) Esters (8) Ketones (9) Terpenes and norisoprenoids (26) Others (7)	[22]
7	Papaya juice (*Carica papaya* L.)	*L. acidophilus* *L. plantarum*	Inoculum: 5% mass ratio at 37 °C for 48 h 5% of edible glucose and 5% of skim milk powder added into the juice	Extracted and analysed using HS-SPME-GC-MS	Acids (8) Alcohols (20) Aldehydes (10) Esters (23) Ketones and lactones (13) Phenols (3)	[23]
8	Pomegranate juice (*Punica granatum* L.)	*L. plantarum*	Inoculum: 7.0 log CFU/mL at 30 °C for 120 h	Extracted and analysed by Purge and trap coupled with gas chromatography-mass spectrometry (PT-GC-MS)	Alcohols (23) Aldehydes (15) Alkanes (8) Alkenes (2) Benzene derivatives (5) Esters (14) Furans (9) Ketones (24) Sulphur compounds (6) Terpenes and norisoprenoids (8) Others (2)	[24]
9	Goji berry/wolfberry juice (*Lycium barbarum* L.)	*L. plantarum* *L. rhamnosus* *L. reuteri* *B. velezensis* *B. licheniformis* Different combinations of mixed culture	Inoculum: 0.5% (*v*/*v*) at 37 °C for 20h under anaerobic conditions pH adjusted to 6.5 using baking soda 5% sucrose added into the juice before pH adjustment	Extracted and analysed using HS-SPME-GC-MS	Acids (5) Alcohols (14) Aldehydes (12) Esters (6) Ketones (7) Phenols (5) Others (9)	[25]
10	Cashew apple juice (*Anacardium occidentale*)	*L. acidophilus* *L. casei* *L. plantarum*	Inoculum: 8.18 log CFU/mL at 37 °C for 48 h	Extracted and analysed using HS-SPME-GC-MS.	Acids (3) Alcohols (8) Aldehydes (1) Esters (6) Ketones (1) Others (1)	[26]
11	Carrot juice (*Daucus carota* L.)	*L. plantarum*	Inoculum: 4.0 log CFU/mL at 37 °C for 120 h	Extracted and analysed using HS-SPME-GC-MS	Alcohols (7) Aldehydes (5) Aromatics (9) Esters (3) Ketones (10) Terpenes and norisoprenoids (17) Others (3)	[27]
12	Broccoli juice (*Brassica oleracea* L. var. *italica*)	*P. pentosaceus*	Inoculum: 8.0 log CFU/mL at 37 °C for 36 h under anaerobic incubator	Extracted and analysed using HS-SPME-GC-MS	Acids (3) Alcohols (8) Aldehydes (4) Esters (2) Furans (3) Ketones (2) Sulfides (3) Others (8)	[28]
13	Cherry juice (*Prunus avium* L.)	*L. plantarum* *L. rhamnosus* *L. paracasei*	Inoculum: 7.0 log CFU/mL at 30 °C (*L. plantarum*), and 37 °C (*L. rhamnosus*, and *L. paracasei*) for 48 h	Extracted and analysed using HS-SPME-GC-MS	Acids (3) Alcohols (15) Aldehydes (9) Benzene derivatives (5) Esters (2) Ketones (7) Terpenes and norisoprenoids (11) Others (5)	[29]
14	Durian pulp (*Durio zibethinus* Murr.)	*L. casei* *W. saturnus* var. *saturnus*	Inoculum: 1% (*v*/*v*) *L. casei* monoculture: at 37 °C for 3 days and 30 °C for 32 days Sequential inoculation: after 3 days of *L. casei* inoculation, 1% (*v*/*v*) *W. saturnus* var. *saturnus* inoculated at 30 °C for another 32 days	Extracted and analysed using HS-SPME-GC-MS	Acids (3) Alcohols (2) Aldehydes (1) Esters (14) Ketones (1) Sulphur containing compounds (4)	[30]
15	Cashew apple juice	*L. plantarum*	Inoculum: 9.0 log CFU/mL at 30 °C for 72 h Fresh cashew apple juice with 11.4 °brix and concentrated cashew apple juice with 78.6 °brix was adjusted to 11.4 °brix with distilled water	Extracted and analysed using HS-SPME-GC-MS	Acids (2) Alcohols (5) Aldehydes (1) Esters (5) Ketones (1) Others (1)	[31]
16	Tomato juice	*L. plantarum* *L. casei*	Inoculum: not reported at 37 °C for 48 h under anaerobic condition Total brix was adjusted from 4.6 to 12.4 °Brix with glucose and sucrose	Extracted and analysed using HS-SPME-GC-MS	Acids (7) Alcohols (46) Aldehydes (10) Esters (6) Hydrocarbons (19) Ketones (17) Others (13)	[32]
17	Yam/Soil potato juice (*Rhizoma dioscoreae*)	*L. plantarum* or *L. plantarum* and *S. thermophilus*	Inoculum: 8.0 log CFU/mL at 37 °C for 24 h The sugar content was adjusted to 20 °Brix	Extracted and analysed using HS-SPME-GC-MS	Acids (13) Alcohols (33) Aldehydes (8) Esters (19) Ketones (8)	[33]
18	Bog bilberry juice (*Vaccinium uliginosum* L.)	*L. plantarum*	Inoculum: 9.0 log CFU/mL at 23 °C for 14 days Juice at nature (2.65) and pH adjusted to 3.50 using NaOH solid powder	Extracted and analysed using HS-SPME-GC-MS	Acids (3) Alcohols (11) Aldehydes (2) Esters (9) Terpenes and norisoprenoids (4) Others (5)	[34]
19	Apple juice, orange juice, carrot juice, and Chinese jujube juice blend	*L. plantarum* *B. breve* *S. thermophilus* mixed starters	Inoculum: 7.0 log CFU/mL at 37 °C for 48 h under anaerobic conditions Blended juice enriched with Se	Extracted and analysed using HS-SPME-GC-MS	Alcohols (11) Esters (4) Hydrocarbons (14) Ketones and Aldehydes (5)	[35]
20	Cabbage Celery Carrot	*L. plantarum* *Lentilactobacillus diolivorans*	Inoculum: 0.5% (*w*/*v*) at 25 °C for 7 days Vegetables (cabbages—8 kg, celeries—1 kg, and Carrots—1 kg) mixed with 500 mL, 4% brine	Extracted and analysed using HS-SPME-GC-MS	Acids (4) Alcohols (11) Aldehydes (6) Alkenes (12) Benzenes (6) Esters (13) Ketones (8) Phenols (3)	[36]
21	Horse gram sprouts (*Macrotyloma uniflorum* var. *uniflorum* Paiyur 1)	*L. plantarum*	Inoculum: 8.0 log CFU/mL (3mL inoculum added to 2 g of crushed sprouts) at 37 °C for 5 days	Extracted and analysed using HS-SPME-GC-MS	Acids (5) Alcohols (8) Aldehydes (3) Esters (6) Ketones (5) Phenols (4) Others (3)	[37]
22	Okara (Soybean pulp)	Co culture of *R. oligosporus* and *Y. lipolytica*	Initial fungal inoculum: 5.2 log CFU *R. oligosporus*/g okara (wet basis) After one day: inoculum 6 log CFU *Y. lipolytica*/g okara (wet basis) at 30 °C for 3 days under aerobic condition	Extracted and analysed using HS-SPME-GC-MS	Acids (6) Alcohols (12) Aldehydes (13) Esters (20) Furans (5) Ketones (10) Phenol and phenol derivatives (5) Others (2)	[38]
23	Cashew apple juice	*L. casei*	Inoculum: 9.0 log CFU/mL at 30 °C for 16 h pH adjusted to 6.4 with 3.0 M NaOH	Extracted and analysed using HS-SPME-GC-MS	Alcohols (1) Esters (9) Terpenes and norisoprenoids (1) Others (1)	[39]
	Melon juice	*L. casei*	Inoculum: 9.0 log CFU/mL at 31 °C for 8 h pH adjusted to 6.1 with 0.1 M citric acid	Extracted and analysed using HS-SPME-GC-MS	Alcohols (5) Aldehydes (8) Esters (18) Ketones (1)	[39]
24	Tomato pomace	*T. atroviride* *A. sojae*	Inoculum: 7.0–8.0 log spores /mL at 30 °C for 120 h in a shaking incubator at 120 rpm	Volatiles extracted by HS-SPME and analysed using GC-MS and GC-Olfactometry (GCO)	Acids (3) Alcohols (6) Aldehydes (7) Esters (1) Ketones (2) Pyrazines (1) Sulphur compounds (1)	[40]
	Red pepper pomace	*T. atroviride* *A. sojae*	Inoculum: 7.0–8.0 log spores/mL at 30 °C for 120 h in a shaking incubator at 120 rpm	Volatiles extracted by HS-SPME and analysed using GC-MS and GC-Olfactometry (GCO)	Acids (2) Alcohols (6) Aldehydes (4) Esters (2) Ketones (6) Pyrazines (1)	[40]
25	Mango slurry	*L. plantarum* *S. thermophilus* *L. casei* *S. cerevisiae*	Inoculum: mass ratio of 5% *S. cerevisiae* at 25 °C for 48 h LAB at 37 °C for 48 h Total brix adjusted to 22.3 °brix with glucose and sucrose	Extracted and analysed using HS-SPME-GC-MS	Acids (5) Alcohols (11) Aldehydes (2) Alkanes (5) Esters (15) Ketones (7) Terpenes and norisoprenoids (10) Others (2)	[41]
26	Sea buckthorn juice (*Hippophae rhamnoides* L.)	*L. plantarum* subsp. *plantarum* *L. plantarum* subsp. *argentoratensis*	Inoculum: 8.30 Log CFU/mL juice at 30 °C for 36 and 72 h Juice at native pH (2.7) and pH adjusted to 3.5 with 1 M NaOH	Extracted and analysed using HS-SPME-GC-MS	Acids (7) Alcohols (6) Aldehydes (7) Alkanes (3) Esters (53) Ketones (9) Terpenes and norisoprenoids (4) Sulphur compounds (3)	[42]
27	Grape juice (Jumeigui variety)	*L. plantarum* *L. brevis* mixed culture 1:2 ratio, respectively	Inoculum: 5% (*v*/*v*) at 36 °C for 60 h	Extracted and analysed using HS-SPME-GC-MS	Acids (8) Alcohols (26) Aldehydes (6) Alkenes (1) Esters (5) Ketones (3) Phenols (2)	[43]
28	Orange pomace (*Citrus sinensis*)	*L. rhamnosus*	Inoculum: 7.0 Log CFU/g at 37 °C for 72 h	Extracted by simple and vacuum distillation Distillates analysed using HS-SPME-GC-MS	Alcohols (18) Aldehydes (12) Esters (7) Furans (3) Ketones (10) Sulphur compounds (1) Terpenes and norisoprenoids (38) Others (2)	[44]
	Melon by-product (*Cucumis melon*)	*L. rhamnosus*	Inoculum: 7.0 Log CFU/g at 37 °C for 72 h	Extracted by simple and vacuum distillation Distillates analysed using HS-SPME-GC-MS	Alcohols (22) Aldehydes (19) Alkenes (1) Esters (25) Furans (1) Ketones (6) Sulphur compounds (2) Terpenes and norisoprenoids (11)	[44]
29	Mung beans	*L. plantarum*	Inoculum: 8.0 log CFU/mL at 37 ± 1 °C for 48 h Mass ratio of mung bean to water of 1:3 (g:g)	Extracted and analysed using a static headspace gas chromatography ion mobility spectrometry (GC-IMS) Aroma characteristics of samples were analysed by electronic nose (E-nose)	Acids (1) Alcohols (5) Aldehydes (9) Alkenes (1) Esters (11) Furans (1) Ketones (6) Others (3)	[45]
30	Okara	*L. acidophilus* *L. rhamnosus* *P. acidilactici* monoculture and co-culture	Inoculum: 6.0–7.0 log CFU/g okara at 37 °C for 72 h Solid state fermentation process	Extracted and analysed using HS-SPME-GC-MS	Alcohols (13) Aldehydes (13) Furans (4) Ketones (9) Others (2)	[46]
31	Jujube pulp (*Ziziphus jujuba* Mill.) (Charkhlik Hui-jujube, Xinjiang variety)	*L. plantarum* *L. rhamnosus* *S. thermophilus* Mixed culture	Inoculum: 8% (*v*/*v*) mixed lactic acid bacteria culture (*L. plantarum*/*L. rhamnosus*/ *S. thermophilus* = 1:1:2, *v*/*v*/*v*) at 37 °C for 24 h	Volatiles extracted by HS-SPME and analysed using GC-MS and GC-Olfactometry (GCO)	Acids (15) Alcohols (7) Aldehydes (9) Esters (5) Ketones (6) Others (7)	[47]
32	Watermelon juice (*Citrullus lanatus* [Thunb])	*L. plantarum* *L. rhamnosus* *L. casei* *L. brevis* *P. pentosaceus*	Inoculum: 1% (*v*/*v*) at 30 °C (*L. plantarum*, *P. pentosaceus* and *L. brevis*) and 37 °C (*L. casei* and *L. rhamnosus*) for 24 h	Extracted and analysed using HS-SPME-GC-MS	Acids (1) Alcohols (15) Aldehydes (14) Alkanes (2) Esters (1) Furans (4) Ketones (6) Terpenes and norisoprenoids (7) Sulphur compounds (1) Others (3)	[48]
33	Kiwifruit juice (*Actinidia deliciosa* cv. Xuxiang and *Actinidia chinensis* cv. Hongyang)	*L. acidophilus* *L. helveticus* *L. plantarum*	Inoculum: 1% (*v*/*v*) at 37 °C for 48 h pH adjusted to 4.0 using food-grade Na_2_CO_3_ (50.0 mg/mL) and brix adjusted to 12.0 °Brix with potable water	Extracted and analysed using HS-SPME-GC-MS	Alcohols (20) Aldehydes (13) Esters (6) Ketones (11) Terpenes and norisoprenoids (6)	[49]
34	Chinese wolfberry juice (*Lycium barbarum*)	*L. plantarum* *L. casei* *L. paracasei* *L. acidophilus* *L. helveticus* *B. lactis*	Inoculum: 0.5% (*v*/*v*) at 37 °C for 48 h	Extracted and analysed using HS-SPME-GC-MS	Acids (3) Alcohols (20) Aldehydes (8) Esters (18) Ketones (11) Terpenes and norisoprenoids (3) Others (1)	[50]
35	Apple juice	*L. plantarum* *S. cerevisiae*	Inoculum: 7% Sequential fermentation: *S. cerevisiae* at 25 °C for 5–6 days, after dealcoholization of fermented juice, then inoculate with *L. plantarum* for 16 h at 39 °C in static condition	Extracted and analysed using HS-SPME-GC-MS	Acids (7) Alcohols (11) Aldehydes (5) Esters (13) Ethers (1) Ketones (10) Olefins (3) Others (1)	[51]

**Table 3:** Flavour notes of important VOCs.

	Flavour Groups	Volatiles	Flavour Notes	Reference
1	Acids			
		Acetic acid	Sharp, pungent, vinegar, cheesy, fatty, sour	[20,22,25]
		Butanoic acid (Butyric acid)	Cheesy, sweet	[20,40]
		2-Methyl-1-butanoic acid	Cheese, sweaty	[31]
		3-Methyl-1-butanoic acid (Isovaleric acid)	Sweet, acid, rancid, stinky feet, cheese, pungent, sour, fruity	[22,26,31,40]
		Hexanoic acid	Sour, fatty, cheesy	[22]
		2-Hexenoic acid (E)	Fruity, sweet, warm, herbal must, fat	[20,22]
		Octanoic acid	Faint, fruity-acid, irritating, brandy, cheese, sweet	[18,20,34,41]
		Hexadecanoic acid	Waxy, fatty	[18]
2	Alcohols			
		Ethanol	Apple, sweet, strong, alcoholic	[20,22,24]
		1-Butanol	Sweetness	[24]
		2-Methyl-1-butanol	Malt, wine, onion, fruity	[17,20,21]
		3-Methyl-1-butanol (Isoamyl alcohol)	Whiskey, alcoholic, malt, burnt, overripe cashew apple, banana	[22,26,31,40]
		1-Pentanol	Fermented yeasty	[28]
		Phenylmethanol (Benzyl alcohol)	Floral	[22]
		2-Phenylethanol/phenyl ethyl alcohol	Floral, soft, rose, jasmine	[22,31,34,40]
		Benzenepropanol	Balsamic	[31]
		1-Hexanol	Light-apple, sweetness, resin, flower, green, herbal ethereal, fruity	[18,20,21,22,24,28,31]
		2-Ethyl hexanol	Rose, sweety, floral, green	[20,21,24]
		(Z)-3-Hexen-1-ol	Grassy-green, leafy	[22,28,34]
		Heptanol	Fruity, green	[20,21,22]
		4-Methyl-4-heptanol	Piney, citrus	[31]
		2,6-Dimethyl-4-heptanol	Fruity, sweet	[31]
		1-Octanol	Pungent, waxy, green, orange	[22,41]
		(E)-2-Octenol	Dust, cement, mushroom	[40]
		1-Octen-3-ol	Earthy, mushroom, vegetable-like	[22,28,34,40]
		Dodecanol	Earthy, soapy, waxy, fatty	[22]
3	Aldehydes			
		Acetaldehyde	Pungent, ether, fruity and juicy	[20,21]
		3-Methylbutanal (Isovaleraldehyde)	Ethereal, aldehydic	[22]
		Benzaldehyde	Almonds, cherry, sweetness, burnt sugar	[18,20,23,24,25,31]
		Hexanal	Fatty, grassy, green	[25,28,40]
		(E)-2-Hexenal	Green leaf, aldehydic, fatty	[19,28]
		Octanal	Fat, lemon, green, soap	[20]
		(E)-2-Octenal	Oxide nutty, mushroom	[40]
		Nonanal	Citrus, green, fruity, fat	[20,21,34]
		(2-Nonenal	Hay, cucumber	[40]
		Decanal	Soap, orange peel, tallow	[20]
4	Esters			
		Butyl acetate	Pear, solvent-like	[21,35]
		Ethyl acetate	Pineapple, fruity, ethereal	[21,22,26,41]
		Methyl 3-methyl butanoate (Methyl isovalerate)	Strong, apple, pineapple	[22]
		3-Methylbutyl acetate (Isoamyl acetate/Isopentyl acetate)	Banana, pear, fruity, sweet	[20,22,34,38,40]
		2-Phenylethyl acetate	Floral, rose, sweet, honey	[22]
		Ethyl butanoate (Ethyl butyrate)	Apple, strawberry, fruity, pineapple	[20,21,34,38]
		Ethyl isobutyrate	Sweet	[21]
		Ethyl 3-methyl butanoate (Ethyl isovalerate)	Fruit, sweet, apple	[22,31]
		Ethyl 2-methyl-2-butenoate	Fruity, cashew-like	[31]
		Propyl butanoate (Propyl butyrate)	Pineapple	[21]
		3-Methylbutyl 3-methyl-butanoate (Isoamyl isovalerate)	Fruity, sweet, green	[22,31]
		(E)-2-Hexenyl acetate	Green, fruity	[22]
		Methyl salicylate	Wintergreen, mint	[22]
		Ethyl hexanoate	Apple peel, fruit, green apple	[20,31,34]
		Ethyl 2-hydroxyhexanoate	Fruity	[31]
		Ethyl octanoate	Fruit, fat	[31]
5	Ketones			
		Acetone	Pungent, ethereal, apple, pear	[20,22,23]
		2-Butanone	Pungent	[23]
		2,3-Butanedione (Diacetyl)	Creamy, butter	[17,20,22,24,40]
		3-Hydroxy-2-butanone (Acetoin)	Milk/dairy, butter, cream, sweet, vanilla	[20,22,23,25,31]
		Methylheptenone	Lemon grass	[19]
		6-Methyl-5-hepten-2-one	Green	[25]
		3-Octanone	Butter, herb, resin	[20]
		2,3-Octanedione	Dill, cooked, broccoli	[22]
6	Furans			
		Furfural	Bread, almond, sweet	[20]
		2-Ethyl furan	Musty, earthy	[28]
		2-Pentyl furan	Floral, grassy, fruity, green, Earthy, beany	[25,28,38]
7	Sulphurs			
		Methanethiol	Pickle, sulphur	[28,40]
		Dimethyl disulfide	Sulfurous, vegetable, cabbage, onion	[28]
		Dimethyl trisulfide	Sulfurous, cooked onion, savory	[28]
8	Terpenes and norisoprenoids			
		(β)-Citronellol	Floral, rose, citrus	[19,22]
		Myrcene	Peppery, spicy	[22]
		Linalool	Flower, lavender, citrus leaf, fruity	[21,22,35]
		D-Limonene	Citrus, lemon, confectionery pineapple, fruity, anise	[22,35]
		(ɤ)-Terpinene	Oily, woody, terpenic, tropical	[22]
		Geraniol	Rose, geranium, sweet, floral, fruity	[21,22]
		(α)-Terpineol	Pine, terpene, lilac, minty, floral, citrusy, orange	[22,35]
		(β)-Ionone	Violet	[25]
		(β)-Damascenone	Woody, sweet, fruity, earthy, stewed apple, iced tea, rose, honey	[20,22,35]
		Myrtenol	Woody, pine, balsam, sweet, mint, medical	[18]

## Data Availability

Not applicable.

## Supplementary Materials

The following supporting information can be downloaded at: https://www.mdpi.com/article/10.3390/molecules28073236/s1, Table S1: The VOCs of fermented fruit and vegetable substrates.

## Sample Availability

Samples of the compounds are not available from the authors.

## References

[1] Lea E.J., Crawford D., Worsley A. (2006). Consumers’ readiness to eat a plant-based diet. Eur. J. Clin. Nutr..

[2] Austgulen M.H., Skuland S.E., Schjøll A., Alfnes F. (2018). Consumer readiness to reduce meat consumption for the purpose of environmental sustainability: Insights from Norway. Sustainability.

[3] Szejda K., Urbanovich T., Wilks M. (2020). Accelerating Consumer Adoption of Plant-Based Meat: An Evidence-Based Guide for Effective Practice.

[4] (2019). BIS Research Report. https://bisresearch.com/industry-report/plant-based-food-beverages-alternatives-market.html.

[5] (2016). Euromonitor Internationals. https://www.euromonitor.com/article/tech-industry-driving-innovation-meat-dairy-analogues.

[6] Astray G., García-Río L., Mejuto J.C., Pastrana L. (2007). Chemistry in food: Flavours. Electron. J. Environ. Agric. Food Chem..

[7] Van Ruth S.M., Roozen J.P., Taylor A.J., Linforth R.S.T. (2010). Delivery of Flavours from Food Matrices. Food Flavour Technology.

[8] Paravisini L., Guichard E. (2016). Interactions between aroma compounds and food matrix. Flavour: From Food to Perception.

[9] Zareian M., Silcock P., Bremer P. (2018). Effect of medium compositions on microbially mediated volatile organic compounds release profile. J. Appl. Microbiol..

[10] Janssens L., De Pooter H.L., Schamp N.M., Vandamme E.J. (1992). Production of flavours by microorganisms. Process Biochem..

[11] Marsili R. (2011). Flavors and off-flavors in dairy foods. Encyclopedia of Dairy Sciences.

[12] Kallscheuer N., Classen T., Drepper T., Marienhagen J. (2019). Production of plant metabolites with applications in the food industry using engineered microorganisms. Curr. Opin. Biotechnol..

[13] Bamforth C.W., Cook D.J. (2019). Food, Fermentation, and Micro-Organisms.

[14] Braga A., Guerreiro C., Belo I. (2018). Generation of flavors and fragrances through biotransformation and de novo synthesis. Food Bioprocess Techol..

[15] Sharma R., Garg P., Kumar P., Bhatia S.K., Kulshrestha S. (2020). Microbial fermentation and its role in quality improvement of fermented foods. Fermentation.

[16] Moher D., Liberati A., Tetzlaff J., Altman D.G., The Prisma Group (2009). Preferred reporting items for systematic reviews and meta-analyses: The PRISMA statement. Phys. Ther..

[17] Cui S., Zhao N., Lu W., Zhao F., Zheng S., Wang W., Chen W. (2019). Effect of different *Lactobacillus* species on volatile and nonvolatile flavor compounds in juices fermentation. Food Sci. Nutr..

[18] Gao H., Wen J.J., Hu J.L., Nie Q.X., Chen H.H., Nie S.P., Xiong T., Xie M.Y. (2019). *Momordica charantia* juice with *Lactobacillus plantarum* fermentation: Chemical composition, antioxidant properties and aroma profile. Food Biosci..

[19] Wu C., Li T., Qi J., Jiang T., Xu H., Lei H. (2020). Effects of lactic acid fermentation-based biotransformation on phenolic profiles, antioxidant capacity and flavor volatiles of apple juice. LWT—Food Sci. Technol..

[20] Li T., Jiang T., Liu N., Wu C., Xu H., Lei H. (2021). Biotransformation of phenolic profiles and improvement of antioxidant capacities in jujube juice by select lactic acid bacteria. Food Chem..

[21] Chen C., Lu Y., Yu H., Chen Z., Tian H. (2019). Influence of 4 lactic acid bacteria on the flavor profile of fermented apple juice. Food Biosci..

[22] Ricci A., Cirlini M., Levante A., Dall’Asta C., Galaverna G., Lazzi C. (2018). Volatile profile of elderberry juice: Effect of lactic acid fermentation using *L. plantarum*, *L. rhamnosus* and *L. casei* strains. Food Res. Int..

[23] Chen R., Chen W., Chen H., Zhang G., Chen W. (2018). Comparative evaluation of the antioxidant capacities, organic acids, and volatiles of papaya juices fermented by *Lactobacillus acidophilus* and *Lactobacillus plantarum*. J. Food Qual..

[24] Di Cagno R., Filannino P., Gobbetti M. (2017). Lactic acid fermentation drives the optimal volatile flavor-aroma profile of pomegranate juice. Int. J. Food Microbiol..

[25] Liu Y., Cheng H., Liu H., Ma R., Ma J., Fang H. (2019). Fermentation by multiple bacterial strains improves the production of bioactive compounds and antioxidant activity of Goji juice. Molecules.

[26] Kaprasob R., Kerdchoechuen O., Laohakunjit N., Sarkar D., Shetty K. (2017). Fermentation-based biotransformation of bioactive phenolics and volatile compounds from cashew apple juice by select lactic acid bacteria. Process Biochem..

[27] Zhang X., Duan W., Zou J., Zhou H., Liu C., Yang H. (2019). Flavor and antioxidant activity improvement of carrot juice by fermentation with *Lactobacillus plantarum* WZ-01. J. Food Meas. Charact..

[28] Xu X., Bi S., Lao F., Chen F., Liao X., Wu J. (2021). Comprehensive investigation on volatile and non-volatile metabolites in broccoli juices fermented by animal- and plant-derived *Pediococcus pentosaceus*. Food Chem..

[29] Ricci A., Cirlini M., Maoloni A., Del Rio D., Calani L., Bernini V., Galaverna G., Neviani E., Lazzi C. (2019). Use of dairy and plant-derived lactobacilli as starters for cherry juice fermentation. Nutrients.

[30] Lu Y., Putra S.D., Liu S.Q. (2018). A novel non-dairy beverage from durian pulp fermented with selected probiotics and yeast. Int. J. Food Microbiol..

[31] Kaprasob R., Kerdchoechuen O., Laohakunjit N., Thumthanaruk B., Shetty K. (2018). Changes in physico-chemical, astringency, volatile compounds and antioxidant activity of fresh and concentrated cashew apple juice fermented with *Lactobacillus plantarum*. J. Food Sci. Technol..

[32] Liu Y., Chen H., Chen W., Zhong Q., Zhang G., Chen W. (2018). Beneficial effects of tomato juice fermented by *Lactobacillus plantarum* and *Lactobacillus casei*: Antioxidation, antimicrobial effect, and volatile profiles. Molecules.

[33] Chen W., Zhu J., Niu H., Song Y., Zhang W., Chen H., Chen W. (2018). Composition and characteristics of yam juice fermented by *Lactobacillus plantarum* and *Streptococcus thermophilus*. Int. J. Food Eng..

[34] Wei M., Wang S., Gu P., Ouyang X., Liu S., Li Y., Zhang B., Zhu B. (2018). Comparison of physicochemical indexes, amino acids, phenolic compounds and volatile compounds in bog bilberry juice fermented by *Lactobacillus plantarum* under different pH conditions. J. Food Sci. Technol..

[35] Xu X., Bao Y., Wu B., Lao F., Hu X., Wu J. (2019). Chemical analysis and flavor properties of blended orange, carrot, apple and Chinese jujube juice fermented by selenium-enriched probiotics. Food Chem..

[36] Chen Z., Kang J., Zhang Y., Yi X., Pang X., Li-Byarlay H., Gao X. (2020). Differences in the bacterial profiles and physicochemical between natural and inoculated fermentation of vegetables from Shanxi Province. Ann. Microbiol..

[37] Goswami R.P., Jayaprakasha G.K., Shetty K., Patil B.S. (2018). *Lactobacillus plantarum* and natural fermentation-mediated biotransformation of flavor and aromatic compounds in horse gram sprouts. Process Biochem..

[38] Chan W., Yi X., Liu S. (2018). Solid-state fermentation with *Rhizopus oligosporus* and *Yarrowia lipolytica* improved nutritional and flavour properties of okara. LWT—Food Sci. Technol..

[39] de Godoy Alves Filho E., Rodrigues T.H.S., Fernandes F.A.N., Pereira A.L.F., Narain N., de Brito E.S., Rodrigues S. (2017). Chemometric evaluation of the volatile profile of probiotic melon and probiotic cashew juice. Food Res. Int..

[40] Güneşer O., Yüceer Y.K. (2017). Biosynthesis of eight-carbon volatiles from tomato and pepper pomaces by fungi: *Trichoderma atroviride* and *Aspergillus sojae*. J. Biosci. Bioeng..

[41] Jin X., Chen W., Chen H., Chen W., Zhong Q. (2018). Comparative evaluation of the antioxidant capacities and organic acid and volatile contents of mango slurries fermented with six different probiotic microorganisms. J. Food Sci..

[42] Markkinen N., Laaksonen O., Yang B. (2021). Impact of malolactic fermentation with *Lactobacillus plantarum* on volatile compounds of sea buckthorn juice. Eur. Food Res. Technol..

[43] Wu B., Liu J., Yang W., Zhang Q., Yang Z., Liu H., Lv Z., Zhang C., Jiao Z. (2021). Nutritional and flavor properties of grape juice as affected by fermentation with lactic acid bacteria. Int. J. Food Prop..

[44] Hadj Saadoun J., Ricci A., Cirlini M., Bancalari E., Bernini V., Galaverna G., Neviani E., Lazzi C. (2021). Production and recovery of volatile compounds from fermented fruit by-products with *Lacticaseibacillus rhamnosus*. Food Bioprod. Process..

[45] Yi C., Li Y., Zhu H., Liu Y., Quan K. (2021). Effect of *Lactobacillus plantarum* fermentation on the volatile flavors of mung beans. LWT—Food Sci. Technol..

[46] Hadj Saadoun J., Calani L., Cirlini M., Bernini V., Neviani E., Del Rio D., Galaverna G., Lazzi C. (2021). Effect of fermentation with single and co-culture of lactic acid bacteria on okara: Evaluation of bioactive compounds and volatile profiles. Food Funct..

[47] Pan X., Zhang S., Xu X., Lao F., Wu J. (2022). Volatile and non-volatile profiles in jujube pulp co-fermented with lactic acid bacteria. LWT—Food Sci. Technol..

[48] Mandha J., Shumoy H., Devaere J., Schouteten J.J., Gellynck X., de Winne A., Athanasia O.M., Raes K. (2021). Effect of lactic acid fermentation of watermelon juice on its sensory acceptability and volatile compounds. Food Chem..

[49] Wang Z., Feng Y., Yang N., Jiang T., Xu H., Lei H. (2022). Fermentation of kiwifruit juice from two cultivars by probiotic bacteria: Bioactive phenolics, antioxidant activities and flavor volatiles. Food Chem..

[50] Qi J., Huang H., Wang J., Liu N., Chen X., Jiang T., Xu H., Lei H. (2021). Insights into the improvement of bioactive phytochemicals, antioxidant activities and flavor profiles in Chinese wolfberry juice by select lactic acid bacteria. Food Biosci..

[51] Li H., Huang J., Wang Y., Wang X., Ren Y., Yue T., Wang Z., Gao Z. (2021). Study on the nutritional characteristics and antioxidant activity of dealcoholized sequentially fermented apple juice with *Saccharomyces cerevisiae* and *Lactobacillus plantarum* fermentation. Food Chem..

[52] Chen C., Zhao S., Hao G., Yu H., Tian H. (2017). Role of lactic acid bacteria on the yogurt flavour: A review. Int. J. Food Prop..

[53] Akhtar M.K., Dandapani H., Thiel K., Jones P.R. (2015). Microbial production of 1-octanol: A naturally excreted biofuel with diesel-like properties. Metab. Eng. Commun..

[54] Aguedo M., Ly M.H., Belo I., Teixeira J.A., Belin J. (2004). The use of enzymes and microorganisms for the production of aroma compounds from lipids. Food Technol. Biotechn..

[55] Pugh S., McKenna R., Halloum I., Nielsen D.R. (2015). Engineering *Escherichia coli* for renewable benzyl alcohol production. Metab. Eng. Commun..

[56] Valera M.J., Boido E., Ramos J.C., Manta E., Radi R., Dellacassa E., Carraua F. (2020). The mandelate pathway, an alternative to the phenylalanine ammonia lyase pathway for the synthesis of benzenoids in ascomycete yeasts. Appl. Environ. Microbiol..

[57] Marilley L., Casey M.G. (2004). Flavours of cheese products: Metabolic pathways, analytical tools and identification of producing strains. Int. J. Food Microbiol..

[58] McSweeney P.L.H., Sousa M.J. (2000). Biochemical pathways for the production of flavour compounds in cheeses during ripening: A review. Le Lait.

[59] Rapp A., Mandery H. (1986). Wine aroma. Experientia.

[60] Corre M., Lubachevsky G., Rankin S.A. (2005). A study of the volatile composition of Minas cheese. LWT—Food Sci. Technol..

[61] Suomalainen H. (1981). Yeast esterases and aroma esters in alcoholic beverages. J. Inst. Brew..

[62] De Carvalho B.T., Holt S., Souffriau B., Brandão R.L., Foulquié-Moreno M.R., Theveleina J.M. (2017). Identification of novel alleles conferring superior production of rose flavor phenylethyl acetate using polygenic analysis in yeast. MBio.

[63] Beresford T.P. (2011). Lactic acid bacteria: Citrate fermentation by lactic acid bacteria. Encyclopedia of Dairy Sciences.

[64] Laëtitia G., Pascal D., Yann D. (2014). The Citrate Metabolism in Homo- and Heterofermentative LAB: A Selective Means of Becoming Dominant over Other Microorganisms in Complex Ecosystems. Food Nutr. Sci..

[65] Quintans N.G., Blancato V., Repizo G., Magni C., López P., Mayo B., López P., Pérez-Martínez G. (2008). Citrate metabolism and aroma compound production in lactic acid bacteria. Molecular Aspects of Lactic Acid Bacteria for Traditional and New Applications.

[66] Wang Y., Wu J., Lv M., Shao Z., Hungwe M., Wang J., Bai X., Xie J., Wang Y., Geng W. (2021). Metabolism Characteristics of Lactic Acid Bacteria and the Expanding Applications in Food Industry. Front. Bioeng. Biotechnol..

[67] Le Bars D., Yvon M. (2007). Formation of diacetyl and acetoin by *Lactococcus lactis* via aspartate catabolism. J. Appl. Microbiol..

[68] Ardö Y. (2006). Flavour formation by amino acid catabolism. Biotechnol. Adv..

[69] Kranenburg R.V., Kleerebezem M., van Hylckama Vlieg J., Ursing B.M., Boekhorst J., Smit B.A., Eman H.E.A., Smit G., Siezen R.J. (2002). Flavour formation from amino acids by lactic acid bacteria: Predictions from genome sequence analysis. Int. Dairy J..

[70] Zaunmüller T., Eichert M., Richter H., Unden G. (2006). Variations in the energy metabolism of biotechnologically relevant heterofermentative lactic acid bacteria during growth on sugars and organic acids. Appl. Microbiol. Biotechnol..

[71] Tsvetanova F., Petrova P., Petrov K. (2018). Microbial production of 1-butanol—Recent advances and future prospects (review). J. Chem. Technol. Metall..

[72] Cheon Y., Kim J.S., Park J.B., Heo P., Lim J.H., Jung G.Y., Seo J.H., Park J.H., Koo H.M., Cho K.M. (2014). A biosynthetic pathway for hexanoic acid production in *Kluyveromyces marxianus*. J. Biotechnol..

[73] Lee S.M., Oh J., Hurh B.S., Jeong G.H., Shin Y.K., Kim Y.S. (2016). Volatile compounds produced by *Lactobacillus paracasei* during oat fermentation. J. Food Sci..

[74] Tan Z., Yoon J.M., Chowdhury A., Burdick K., Jarboe L.R., Maranas C.D., Shanks J.V. (2018). Engineering of *E. coli* inherent fatty acid biosynthesis capacity to increase octanoic acid production. Biotechnol. Biofuels.

[75] Zhang Y., Nielsen J., Liu Z. (2017). Engineering yeast metabolism for production of terpenoids for use as perfume ingredients, pharmaceuticals and biofuels. EMS Yeast Res..

[76] Moser S., Pichler H. (2019). Identifying and engineering the ideal microbial terpenoid production host. Appl. Microbiol. Biotechnol..

[77] Rannou C., Laroque D., Renault E., Prost C., Sérot T. (2016). Mitigation strategies of acrylamide, furans, heterocyclic amines and browning during the Maillard reaction in foods. Food Res. Int..

[78] Amann A. (2016). Characterization and Pathway Investigation of Off-Flavor Formation in Stored Commercial Apple and Orange Juice Products. Retrieved from the University of Minnesota Digital Conservancy. https://hdl.handle.net/11299/178913.

[79] Rauhut D., König H., Unden G., Fröhlich J. (2017). Usage and formation of sulphur compounds. Biology of Microorganisms on Grapes, in Must and in Wine.

