# Supplementary material for: Flavour Volatiles of Fermented Vegetable and Fruit Substrates: A Review
Source: Molecules. 2023 Apr 4;28(7):3236. doi: 10.3390/molecules28073236 (PMC10096934; doi:10.3390/molecules28073236)
Supplement: Supplementary file 1 [file molecules-28-03236-s001.zip › molecules-2212445-supplementary.pdf]

Table S1: The VOCs of fermented fruit and vegetable substrates

No	VOCs	Reference
1	Acid; acetic acid<br><br>Alcohols; 3,7-dimethyl-1,6-octadien-3-ol, and 2-methylbutanol<br><br>Esters; formic acid, hexyl ester, and acetic acid, and hexyl ester<br><br>Ketones; 2,3-butanedione (diacetyl), 3-hydroxybutanone (acetoin), and 6-methyl-5-hepten-2-one<br><br>Aldehydes; 3-methyl-benzaldehyde, and (e)-2-hexenal	[17]
2	Alcohols; (e)-2-penten-1-ol, 3-methyl-1-butanol, (e)-3-hexen-1-ol, (z)-3-hexen-1-ol, (e)-2-hexen-1-ol, 1-hexanol, 1-heptanol, 1-octen-3-ol, 1-cyclohexene-1-methanol, 2-ethyl-1-hexanol, benzyl alcohol, 2-octyn-1-ol, 3-octyn-1-ol, 1-nonen-4-ol, 2-phenylethanol, 2-nonen-1-ol, 1-nonanol, 2-undecanol, p-mentha-1,4-dien-7-ol, and hexadecanol	[18]

	Aldehydes; benzaldehyde, 1,2-benzenedicarboxaldehyde, (e,z)-2,4-heptadienal, octanal, (e,e)-2,4-heptadienal, phenylacetaldehyde, (e)-2-octenal, nonanal, (e)-2-nonenal, decanal, (e)-2-decenal, 2-undecenal, pentadecanal, hexanal, (e)-2-hexenal, heptanal, and (e,e)-2,4-hexadienal<br><br>Terpenoids; p-cymene, linalool, dihydrocarveol, α-terpineol, myrtenol, β-cyclocitral, geraniol, α-ionone, geranyl acetone, β-ionone, cedrol, widdrol, and hexahydrofarnesyl acetone<br><br>Ketones; 2-heptanone, 1-octen-3-one, 6-methyl-5-hepten-2-one, 2-nonanone, 2-undecanone, β-damascenone, 6,10-dimethyl-2-undecanone, and 2-tridecanone<br><br>Esters; dihydroactinidiolide, diisobutyl phthalate, and methyl hexadecanoate<br><br>Hydrocarbons; 1,4-hexadiene 3-ethyl-, tetradecane, pentadecane, and hexadecane<br><br>Heterocyclic compounds; furfural, and 2-pentylfuran<br><br>Acids; hexanoic acid, octanoic acid, nonanoic acid, and hexadecanoic acid<br><br>Others; 4-(2-methyl-3-oxocyclohexyl) butanal, and 2-tetradecyloxirane	
3	Alcohols; ethanol, 2-methyl-1-propanol, 1-butanol, isobutenylcarbinol, 3-methyl-1-butanol, 2-methyl-1-butanol, 1-pentanol, trans, 2-hexen-1-ol, hexyl alcohol, benzyl alcohol, 1-octanol, β-citronellol, and geraniol	[19]

	Esters; ethyl acetate, ethyl propionate, propyl acetate, methyl butyrate, isobutyl acetate, methyl 2-methylbutyrate, ethyl butyrate, propyl propionate, butyl acetate, ethyl 2-methylbutyrate, 2-methylbutyl acetate, propyl butyrate, butyl propionate, amyl acetate, prenyl acetate, methyl hexanoate, propyl 2-methylbutanoate, isobutyl butyrate, ethyl caproate, hexyl acetate, butyl 2-methylbutanoate, ethyl caprylate, phenethyl acetate, and ethyl caprate<br><br>Ketones; 2,3-butanedione, 2-pentanone, 2,3-pentanedione, acetoin, 2-heptanone, methylheptenone, 2-octanone, 2-nonanone, 2-undecanone, and damascenone<br><br>Aldehydes: acetaldehyde, hexanal, (e)-2-hexenal, nonanal, and decanal	
4	Alcohols; ethanol, pentanol, butanol, 3-methyl-1-butanol, 2-methyl-1-butanol, hexanol, heptanol, octenol, benzyl alcohol, and 2-ethylhexanol<br><br>Aldehydes: acetaldehyde, isobutyraldehyde, trans-crotonaldehyde, 3-methylbutanal, 2-methyl-1-butanal, pentanal, hexanal, furfural, heptanal, benzaldehyde, octanal, 2-dodecenal, 2-octenal, p-tolualdehyde, 2-methylbenzaldehyde, 3-methylbenzaldehyde, nonanal, decanal, and γ-nonalactone<br><br>Acids; formic acid, acetic acid, isobutyric acid, butyric acid, methylbutyric acid, pentanoic acid, caproic acid, (e)-2-hexenoic acid, heptanoic acid, 2-methylhexanoic acid, benzoic acid, octanoic acid, nonanoic acid, and decanoic acid	[20]

	Ketones; acetone, diacetyl, pentanone, acetoin, hexanone, heptanone, 4-hexen-3-one, octenone, 3-octanone, acetophenone, nonanone, and β-damascenone<br><br>Esters; ethyl acetate, ethyl butanoate, hexyl formate, isoamyl acetate, ethyl hexanoate, β-phenethyl acetate, ethyl decanoate, and isopropyl myristate	
5	Terpenes/norisoprenoids; isoprene, myrcene, d-limonene, ocimene, linalool, and eugenol<br><br>Ketones; 4-heptanone, 2-octanone, 6-methyl-5-hepten-2-one, 2-nonanone, 4-cyclopentene-1,3-dione, and geranylacetone<br><br>Alcohols; 2-methyl-1-butanol, 3-methyl-3-butenol, 2-heptanol, 1-hexanol, 6-methyl-5-hepten-2-ol, 2-ethylhexanol, 1-octanol, cis-5-octen-1-ol, menthol, 2-nonanol, 2-furanmethanol, α-terpineol, geraniol, benzyl alcohol, 1-butanol, and 1,3-octanediol<br><br>Aldehydes; hexanal, nonanal, furfural, benzaldehyde, and acetaldehyde<br><br>Esters; ethyl acetate, butyl acetate, ethyl isobutyrate, ethyl butyrate, methyl 2-methylbutyrate, ethyl propionate, ethyl-2-methylbutyrate, 2-methylbutyl acetate, propyl butyrate, propyl 2-methylbutanoate, isoamyl isovalerate, and butyl butyrate	[21]

	Acids; isobutyric acid, and butanoic acid<br><br>Phenols; 2,6-di-tert-butyl-4-methylphenol, phenol, 4-ethyl-2-methoxyphenol, and 4-vinylphenol-2-methoxy	
6	Ketones; acetone, diacetyl, acetoin, 2.3-octanedione, sulcatone (6-methyl-5-hepten-2-one), 2,3,4,5-tetramethyl-2-cyclopenten-1-one, herbal ketone, 2-undecanone, and 2-tridecanone<br><br>Esters; ethyl acetate, methyl isovalerate, ethyl isovalerate, isopentyl acetate, isoamyl isovalerate, (e)-2-hexenyl-acetate, methyl salicylate, and phenethyl acetate<br><br>Aldehydes; isovaleraldehyde, 3-(2,3,6-trimethyl-1-cyclohexen-1-yl)-2-propenal, and 3-(2,3,6-trimethyl-1-cyclohexen-1-yl)-2-propenal "like"<br><br>Alcohols; 1-pentanol, ethanol, isoamyl alcohol, 2-penten-1-ol, hexanol, (e)-3-hexen-1-ol, (z)-3-hexen-1-ol, 3-octanol, (e)-2-hexen-1-ol, 1-octen-3-ol, heptanol, octanol, 6-methyl-heptanol, 6-methyl-octanol, nonanol, 2-furanmethanol, 2-phenylmethanol, 2-phenylethanol, and dodecanol<br><br>Terpenes and norisoprenoids; 3-carene, myrcene, limonene, eucalyptol, γ-terpinene, m-cymene, terpinolene, α-ionene, cis-linalool oxide, β-linalool, β-caryophyllene, hotrienol, dihydro-citronellol, α-terpineol, cadina-1(10)-4-	[22]

	diene, β-damascenone “like”. B-citronellol (r), cis-geraniol, β-damascenone, trans-geraniol, cis-lanceol, trans-nerolidol, eugenol, and terpene not identified (3)<br><br>Alkenes; styrene, 3,4-dimethyl octene, and 1-2-4 trimethyl-1h-indene<br><br>Acids; acetic acid, isovaleric acid, caproic acid, caproic acid like, 2-hexenoic acid (e), and caprylic acid<br><br>Others; dimethyl disulfide, dimethyl trisulfide, ionone e benzaldehyde (co-elution), phenol, 4-ethyl-phenol, unknown (2)	
7	Acids; acetic acid, 2-methyl-propanoic acid, butanoic acid, 3-methyl-butanoic acid, 2-methyl-butanoic acid, 2-ethyl-hexanoic acid, octanoic acid, and nonanoic acid<br><br>Alcohols; ethanol, 1-propanol, 2-methyl-1-propanol, 1-butanol, 1-penten-3-ol, 3-methyl-1-butanol, 3-methyl-3-buten-1-ol, 1-pentanol, prenol, 3-pentanol, 1-hexanol, 1-heptanol, linalool, 1-octanol, 1-nonanol, 1-decanol, geraniol, benzyl alcohol, phenylethyl alcohol, and nerolidol<br><br>Aldehydes; acetaldehyde, pentanal, hexanal, 2-methyl-2-heptenal, heptanal, octanal, nonanal, benzaldehyde, dodecanal, and 2,4-dimethyl-benzaldehyde	[23]

	Esters; acetic, methyl ester, acetic, ethyl ester, butanoic acid, methyl ester, butanoic acid, ethyl ester, hexanoic acid, methyl ester, hexanoic acid, ethyl ester, thiocyanic acid, methyl ester, acetic acid, 2-ethylhexyl ester, octanoic acid, methyl ester, octanoic acid, ethyl ester, n-butyric acid 2-ethylhexyl ester, decanoic acid, methyl ester, decanoic acid, ethyl ester, hexanoic acid, 2-ethylhexyl ester, dodecanoic acid, methyl ester, dodecanoic acid, ethyl ester, tetradecanoic, methyl ester, tetradecanoic acid, ethyl ester, benzoic acid, 2-ethylhexyl ester, dodecanoic acid, ethyl ester, 9-hexadecenoate, ethyl ester, 1,2-benzenedicarboxylic acid, bis(2-methylpropyl) ester, and dibutyl phthalate<br><br>Ketones and lactones; acetone, 2-butanone, 2,3-butanedione, 2-heptanone, acetoin, 1-hydroxy-2-propanone, 6-methyl-5-hepten-2-one, 2-hydroxy-3-pentanone, 2-nonanone, 3-(hydroxymethyl)-2-nonanone, 2-octanone, 2-dodecanone, and 2-tetradecanone<br><br>Phenols; 2,6-bis(1,1-dimethylethyl)-4-(1-oxopropyl) phenol, phenol, and 2,4-di-tert-butylphenol	
8	Aldehydes; acetaldehyde, butanal, pentanal, propenal, 2-methyl-propenal, 2-butenal, 2-pentenal, 2-hexenal, 2-heptenal, 2-octenal, 2-methyl-propanal, 3-methyl-butanal, 2-methyl-butanal, benzaldehyde, and benzeneacetaldehyde<br><br>Alcohols; methanol, ethanol, 1-propanol, 1-butanol, 1-pentanol, 1-hexanol, 1-penten-3-ol, 2-penten1-ol (z), 3-hexen1-ol (e), 3-hexen-1-ol (z), 1-octen-3-ol, 2-propanol, 2-butanol, 2-pentanol, 2-heptanol, 2-methyl-1-propanol, 3-methyl-1-butanol, 2-methyl-1-butanol, 2-methyl-2-propanol, 2-methyl-2-butanol, 3-methyl-2-butanol, 3-methyl-3-buten-1-ol, and 2-ethyl-1-hexanol	[24]

	Ketones; 2-propanone, 2-butanone, 2-pentanone, 3-pentanone, 2-hexanone, 2-heptanone, 2-octanone, 2-nonanone, 2-decanone, 3-hexanone, 3-heptanone, 3-buten-2-one, 1-penten-3-one, 1-octen-3-one, 2,3-butanedione, 2,3-pentanedione, 2,3-hexanedione, 3-methyl-2-butanone, 4-methyl-2-pentanone, 2-methyl-3-pentanone, 3-methyl-2-pentanone, 4-methyl-2-heptanone, 6-methyl-2-heptanone, and 6-methyl-5-hepten-2-one<br><br>Esters; methyl acetate, methyl propanoate, methyl butanoate, methyl hexanoate, methyl 2-propenoate, methyl2-methyl-2-propenoate, ethyl acetate, ethyl propanoate, ethyl butanoate, ethyl hexanoate, propyl acetate, 2-pentyl acetate, isopropyl acetate, and 2-methyl-propyl acetate<br><br>Sulfur compounds; methanethiol, dimethyl-sulfide, dimethyl-disulfide, carbon disulfide, thiophene, and s-methyl thioacetate<br><br>Furans; 2-methyl-furan, 2-ethyl-furan, 2-propyl-furan, 2-pentyl furan, 3-methyl-furan, 2,5-dimethyl-furan, 2,4-dimethyl-furan, 2-acetyl furan, and furfural<br><br>Alkanes; butane, pentane, heptane, octane, nonane, decane, undecane, and dodecane<br><br>Alkenes; 2-methyl-1-propene, and 2-methyl-1,3-butadiene<br><br>Benzene derivatives; benzene, methylbenzene, 1,3,5-trimethyl-benzene, naphthalene, and p-methyl-ethenyl-toluene	

	Terpenes; camphene, beta-myrcene, p-cymene, limonene, alpha-terpinolene, alpha terpinene, 1,8-cineole, and gamma terpinene<br><br>Miscellaneous; diethyl-ether, and 2-ethoxy-2-methylpropane	
9	Acids; formic acid, acetic acid, heptanoic acid, octanoic acid, and n-hexadecanoic acid<br><br>Alcohols; dimethyl-silanediol, 1-hexanol, 1-octen-3-ol, benzyl alcohol, 1-octanol, isophytol, linalool, phenylethyl alcohol, (z)-3-nonen-1-ol, 1-nonanol, levomenthol, α-terpineol, geraniol, and 2,4-decadien-1-ol<br><br>Ketones; acetoin, 6-methyl-5-hepten-2-one, 6-methyl-3,5-heptadiene-2-one, 2-n-hexylcyclopentanone, (e)-1-(2,6,6-trimethyl-1,3-cyclohexadien-1-yl)-2-buten-1-one, (e)-6,10-dimethyl-5,9-undecadien-2-one, and trans-β-ionone<br><br>Aldehydes; hexanal, (e)-2-heptenal, benzaldehyde, (e,e)-2,4-heptadienal, benzeneacetaldehyde, (e)-2-octenal, (e)-2-nonenal, 2,6,6-trimethyl-1,3-cyclohexadiene-1-carboxaldehyde, 3,5-dimethyl-benzaldehyde, (e,e)-2,4-decadienal, (e,e)-2,6-nonadienal, and trans-4,5-epoxy-(e)-2-decenal<br><br>Esters; methoxy-phenyl-oxime, formic acid heptyl ester, 1,2-benzenedicarboxylic acid bis(2-methylpropyl) ester, hexadecanoic acid methyl ester, dihydro-5-pentyl-2(3h)-furanone, and hexadecanoic acid ethyl ester	[25]

	Phenols; 2-methoxy-phenol, 2,5-dimethyl-phenol, 2,4,5-trimethyl-phenol, 2-methoxy-4-vinylphenol, and 2,4-di-tert-butylphenol<br><br>Others; 2-pentyl-furan, naphthalene, 4-(9-borabicyclo [3.3.1] non-9-yloxy)-2-thiapentane, 2,3-dihydro-2,2-dimethyl-7-benzofuranol, 4-(1h-pyrazol-1-yl) benzeneamine, n, n-dibutyl-formamide, 2,3-nonadiene, (r)-5,6,7,7a-tetrahydro-4,4,7a-trimethyl-2(4h)-benzofuranone, and 3-ethyl-2-methyl-1,3-hexadiene	
10	Alcohols; 2,6-dimethyl-4- heptanol, 2-phenylethanol, 3,6-dimethoxy-9-(2-phenylethynyl)-fluoren-9-ol, benzenepropanol, 3-methyl-1-butanol, ethanol, 4-methyl-4-heptanol, and 1-hexanol<br><br>Acids; 2-methyl butanoic acid, ethyl-3-methyl butanoic acid, and acetic acid<br><br>Esters; ethyl-3-methyl-butanoate, ethyl-(2e)-2-methyl-butenoate, ethyl hexanoate, 3-methylbutyl-3-methyl-butanoate, ethyl acetate and ethyl octanoate<br><br>Ketones; 3-hydroxy-2-butanone (acetoin)<br><br>Aldehydes; benzaldehyde<br><br>Others; 1-methyl-2-phenyl-1h-indole	[26]

11	Terpenes; α-pinene, camphene, sabinene, β-mycrene, d-limonene, γ-terpinene, terpinolene, β-caryophyllene, α-caryophyllene, β-farnesene, valencene, germacrene d, α-farnesene, β-bisabolene, γ-bisabolene, α-patchoulene, and curcumene<br><br>Aldehyde; hexanal, heptanal, benzaldehyde, 2-nonenal, and β-cyclocitral<br><br>Ketones; 3-hydroxy-2-butanone, octanone, 6-methyl-5-hepten-2-one, 2-nonanone, camphor, damascenone, α-ionone, geranylaceton, β-ionone, and perhydrofarnesyl acetone<br><br>Alcohols; 1-heptanol, 2-ethylhexanol, 2,3-butanediol, linalool, 1-octanol, 4-terpineol, and 1-nonanol<br><br>Esters; vinyl acetate, bornyl acetate, and neryl acetate<br><br>Aromatics; p-cymene, 2,4-dimethyl styrene, benzeneacetaldehyde, p-methylacetophenone, 3,4-dimethylbenzaldehyde, guaiacol, phenethyl alcohol, 2-methoxy-4-vinylphenol, and elemicin<br><br>Others; furfural, 2-pentanecarboxylic acid, and myristicin	[27]
12	Acids; hexanoic acid, octanoic acid, and nonanoic acid	[28]

	Alcohols; 1-hexanol, 1-octen-3-ol, 2-hexen-1-ol, (e)-, 3-hexen-1-ol, (e), 3-hexen-1-ol, (z), 2-penten-1-ol, (z), 1-nonen-4-ol, and 1-pentanol<br><br>Aldehydes; 2,4-heptadienal, (e, e), 2-hexenal, benzaldehyde, and benzaldehyde, 4-ethyl<br><br>Ketones; 2-octanone, and 4-methyl-2- heptanone<br><br>Sulfides; dimethyl trisulfide, dimethyl disulfide, and thiophene,2-ethyl<br><br>Esters; thiocyanic acid, methyl ester, and methyl tetradecanoate<br><br>Furans; cis-2-(2-pentenyl) furan, furan, 2-ethyl-, and furan, 2-pentyl-<br><br>Others; p-xylene, ethyl benzene, benzene, 1,3-bis(1,1-dimethylethyl), benzenepropanenitrile, benzene, 1-2 dimethoxy, benzyl nitrile, 2,4-pentadienenitrile, and phenol, 2,4-bis (1,1-dimethylethyl)-	
13	Ketones; acetone, 2-heptanone, 4-methyl-2-heptanone, ethyl isoamyl ketone, 1-decen-3-one, acetoin, and 2-nonanone	[29]

	Alcohols; ethanol, isopentyl alcohol, 3-pentenol, isobutenylcarbinol, hexanol, (e)-2-hexen-1-ol, amyl vinyl carbinol, 1-heptanol, 2-ethyl-1-hexanol, octanol, 1-nonanol, 2-furanmethanol, decanol, benzene methanol, and benzene ethanol<br><br>Aldehydes; isovaleraldehyde, octanal, 2-methyl-2-octanal, nonanal, furfural, benzaldehyde, safranal, p-tolualdehyde, and α,4-dimethyl-3-cyclohexene-1-acetaldehyde<br><br>Terpenes, terpenic derivatives and norisoprenoids; unidentified terpene, limonene, m-cymene, prenol, α-ionene, trans-linalool dioxide, β-linalool, p-menthen-8-ol, trans-geraniol, p-mentha-1(7), 8(10)-dien-9-ol, and eugenol<br><br>Acids; acetic acid, pivalic acid, and 4-hydroxybutanoic acid<br><br>Esters; propyl acetate, and methyl salicylate<br><br>Derivatives of benzene; ethyl benzene, styrene, m-di-tert-butylbenzene, naphthalene, and 1-1-6-trimethyl-1,2-dihydronaphthalene (tdn)<br><br>Others; 2-ethoxy-2-methyl-propane, 2-pentylfuran, dimethyl trisulfide, 4-methyldihydro-2(3h)-furanone, and 4-ethyl-phenol	
14	Alcohols; ethanol, and 2-phenylethyl alcohol	[30]

	Aldehydes; benzaldehyde<br><br>Acids; acetic acid, octanoic acid, and decanoic acid<br><br>Ketones; acetoin<br><br>Sulphur containing compounds; methyl ethyl disulphide, diethyl disulphide, 1,2,4-trithiolane, 3,5-dimethyl (isomer 1), and 1,2,4-trithiolane, 3,5-dimethyl (isomer 2)<br><br>Esters; ethyl acetate, isoamyl acetate, 2-phenylethyl acetate, methyl-2methylbutanoate, methyl octanoate, methyl decanoate, ethyl 2-methylbutanoate, ethyl hexanoate, ethyl octanoate, ethyl decanoate, ethyl tetradecanoate, ethyl hexadecenoate, ethyl 9-hexadecenoate, and propyl 2-methylbutanoate	
15	Alcohols; 3-methyl-1-butanol, 1-hexanol, 2,6-dimethyl-4-heptanol, 4-methyl-4-heptanol, and benzenepropanol<br><br>Esters; ethyl-3-methyl-butanoate (ethyl isovalerate), ethyl-(2e)-2-methyl-2-butenoate ((e)- ethyl tiglate), ethyl hexanoate, 3-methylbutyl-3-methyl-butanoate (isoamyl isovalerate), and ethyl octanoate (ethyl caprylate)<br><br>Aldehydes; benzaldehyde	[31]

	Ketones; 3-hydroxy-2-butanone (acetoin)<br><br>Acids; 3-methyl butanoic acid (isovaleric acid), and 2-methyl butanoic acid<br><br>Others; 1-methyl-2-phenyl-1h-indole	
16	Alcohols; ethanol, 1-butanol, 3-methyl-, 1-pentanol, 1-penten-3-ol, 1-hexanol, 3-hexen-1-ol, (z)-, 2-hepten-1-ol, (e)-, 1-hexanol, 4-methyl-, (s)-, 1-octen-3-ol, 1-heptanol, 6-hepten-1-ol, 2-methyl-, 1-octanol, 1-methylcycloheptanol, phenylethyl alcohol, 2-hexen-1-ol, (z)-, alpha.-terpineol, 3-nonen-1-ol, (z)-, trans-2-undecen-1-ol, 2-propyl-1-pentanol, 1-pentanol, 3-methyl-, 2-penten-1-ol, (z)-, 3-hexen-1-ol, acetate, (z)-, 2-penten-1-ol, (z)-, 3-buten-1-ol, 3-methyl-, 4-hexen-1-ol, 2-nonanol, trans-(2-ethylcyclopentyl)methanol, cis-9-tetradecen-1-ol, 2-furanmethanol, 2-nonen-1-ol, (e)-, geraniol, 5,9-undecadien-2-ol, 6,10-dimethyl-, bicyclo[3.1.1]hept-2-ene-2-methanol, 6,6-dimethyl, 1,6-octadien-3-ol, 3,7-dimethyl-, 3-hexen-1-ol, (e)-, 1-hexanol, 2-ethyl-, 3-furanmethanol, 2-buten-1-ol, 3-methyl-, 1-pentanol, 4-methyl-, 3-hexen-1-ol, acetate, (e)-, 6-hepten-1-ol, 2-methyl-, 1-methylcycloheptanol, 2-tridecen-1-ol, (e)-, citronellol, 3-nonen-1-ol, (z)-, and 5,9-undecadien-2-ol, 6,10-dimethyl-<br><br>Hydrocarbons; benzene, 1,3-dimethyl-, 3-carene, o-xylene, styrene, cyclohexane, ethylidene-, pentane, 2-nitro-, 5-hepten-2-one, 6-methyl, bicyclo[2.2.1]hept-2-ene, 1,7,7-trimethyl, hexadecane, 2,6,10,14-tetramethyl, 1-tetradecyne, 1-hexene, 3,3,5-trimethyl, heneicosane, 4-tridecene, (z), d-limonene, butylated hydroxytoluene, benzene, 1-methoxy-4-methyl, 1-hexene, 3,3-dimethyl-, 3-nonen-5-yne, 4-ethyl-, and 3-buten-2-one, 4-(2,6,6-trimethyl-1-cyclohexen-1-yl)-	[32]

	Aldehydes: hexanal, 2-hexenal, (e), nonanal, decanal, 2-nonenal, (e), 2,6-octadienal, 3,7-dimethyl-, (e), 1h-pyrrole-2-carboxaldehyde, 2-undecenal, e, 2-octenal, (e), and 2,6-octadienal, 3,7-dimethyl-, (z)<br><br>Ketones; acetone, 4'-(trifluoromethyl)acetophenone, 3-buten-2-one, 4-(2,2,6-trimethyl-7-oxabicyclo[4.1.0]hept-1-yl), 4-hydroxy-2-methylacetophenone, 2-undecanone, 5-hepten-2-one, 6-methyl, 2-nonanone, 2-pentanone, 2-propanone, 1-hydroxy, acetoin, 2-cyclopenten-1-one, 3,4,4-trimethyl, 2-heptanone, 2,3-butanedione, 2h-pyran-2-one, tetrahydro-, 3,5,9-undecatrien-2-one, 6,10-dimethyl, 2(4h)-benzofuranone, and 5,6,7,7a-tetrahydro-4,4,7a-trimethyl-, (r)<br><br>Acids; acetic acid, butanoic acid, 3-methyl-, butanoic acid, anhydride, hexanoic acid, octanoic acid, cyclohexylmethyl formate, and ammonium acetate<br><br>Esters; 3-nonen-5-yne, 4-ethyl-, methyl undecyl ether, ethyl tridecanoate, acetic acid, butyl ester, dodecanoic acid, methyl ester, and tetradecanoic acid, ethyl ester<br><br>Others; 2-isobutylthiazole, oxime-, methoxy-phenyl-, benzofuran, 2,3-dihydro-, phenol, 2,4-bis(1,1-dimethylethyl)-, d-limonene, butanenitrile, 3-methyl-, oxepine, 2,7-dimethyl-, furfural, ethyl tridecanoate, phenol, methyl tetradecanoate, bicyclo[3.1.1]hept-2-ene-2-methanol, 6,6-dimethyl- , and 2-ethylhexyl salicylate	
17	Alcohols; 2-ethyl-1-hexanol, 1-dodecanol, 1-octanol, 1-nonanol, geraniol, 3,7-dimethyl-1,6-octadien-3-ol, 2-undecanol, 3,7,11-trimethyl-1-dodecanol, 1-heneicosanol, 2-tridecanol, α-terpineol, 2-(2-hydroxypropoxy)-1-	[33]

	propanol, 1-octen-3-ol, 5-methyl-2-(1-methylethyl)-(1alpha,2alpha,5alpha)- cyclohexanol, 2-nonanol, 2-methyl-tridecane-2,12-diol, 2-heptanol, acetate, n-tridecan-1-ol, n-heptadecanol-1, maltol, cyclododecanemethanol, phenylethyl alcohol, 2-decanol, 2-ethyl-2-methyl-tridecanol, 3,7,11,15-tetramethyl-1- hexadecanol, 2-hexyl-1-decanol, 2-octyl-1-decanol, n-tetracosanol-1, β-(1-ethoxyethoxy)-[2r-[2r*[r*(r*)]]]- oxiraneethanol, cis-9-tetradecen-1-ol, p-menthane-1,3-diol, 1-tetradecanol, and trans-2-dodecen-1-ol<br><br>Acids; octanoic acid, nonanoic acid, n-hexadecanoic acid, acetic acid, dodecanoic acid, oleic acid, 5-hexenoic acid, n-decanoic acid, pentadecanoic acid, 2-ethyl-hexanoic acid, methyliminodiacetic acid, (e)-3-decenoic acid, and cis-5-dodecenoic acid<br><br>Esters; diethylmalonic acid, di(2-(3,3-dimethyl- 2,4- oxacyclopentyl)ethyl) ester, 2,2,4-trimethyl-1,3-pentanediol diisobutyrate, sulfurous acid, cyclohexylmethyl heptadecyl ester, ethyl acetate, phthalic acid, butyl undecyl ester, sulfurous acid, cyclohexylmethyl hexadecyl ester, formic acid, octyl ester, heptafluorobutyric acid, n-octadecyl ester, cyclopropaneoctanoic acid, 2-hexyl-, methyl ester, ethyl dodecyl ether, glycine, n-[4-[(trimethylsilyl)oxy]benzoyl]- methyl ester, nitric acid, nonyl ester, decanoic acid, decyl ester, fumaric acid, non-5-yn-3-yl pentadecyl ester, formic acid, hexyl ester, hexadecanoic acid, methyl ester, methyl 7,9-tridecadienyl ether, 6-amino-5-cyano-4- (3-iodo-phenyl)-2- methyl-4h-pyran-3- carboxylic acid ethyl ester, and (z)-dodec-5-en-4-olide ester<br><br>Aldehydes; nonanal, octanal, hexanal, octadecanal, 2-undecenal, 2,5-bis[(trimethylsilyl)oxy]- benzaldehyde, undecanal, and tridecanal	

	Ketones; 2, heptanone, 2-nonanone, 2-undecanone, 3-methyl-4-methylene-2- hexanone, dihydro-5-pentyl-2(3h)-furanone, 3-[3-bromophenyl]-7-chloro-3,4- dihydro-10-hydroxy- 1,9(2h,10h)- acridinedione, 2-pentadecanone, and 3-amino-5-(4-morpholinylmethyl)- 2-oxazolidinone	
18	Terpenoids; α-terpineol, linalool, 6-methyl-5-hepten-2-one, and citronellyl formate<br><br>Alcohols; phenylethyl alcohol, (z)-3-hexen-1-ol, 1-hexanol, 1-heptanol, 1-octen-3-ol, 1-octanol, 3-octanol, 2-ethyl-1-hexanol, benzyl alcohol, 1-dodecanol, and 2-heptanol<br><br>Acids; octanoic acid, hexanoic acid, and pentanoic acid<br><br>Esters; ethyl nonanoate, ethyl octanoate, ethyl laurate, isopentyl acetate, ethyl hexanoate, ethyl butanoate, methyl salicylate, isobutyl acetate, and ethyl phenylacetate<br><br>Aldehydes; benzaldehyde, and nonanal<br><br>Others; styrene, cyclohexene, naphthalene, β-methylnaphthalene, and α-methylnaphthalene	[34]
19	Alcohols; 2-hexen-1-ol, (e)-, 1-hexanol, benzyl alcohol, linalool, fenchol, β-terpineol, borneol, terpinen-4-ol, α-terpineol, cis-carveol, and carveol	[35]

	Esters; formic acid, octyl ester, hexanoic acid, 3-hydroxy-ethyl ester, bornyl acetate, and neryl acetate<br><br>Ketones and aldehydes; ethanone, 1-(3-methylphenyl)-, decanal, benzaldehyde, 3,5-dimethyl-, d-carvone, and β-damascenone<br><br>Hydrocarbons; d-limonene, caryophyllene, cis-α-bergamotene, (e)-β-farnesene, humulene, γ-selinene, valencene, β-bisabolene, (+)-β-himachalene, cis-α-bisabolene, p-cymenene, eudesma-3,7(11)-diene, α-curcumene, and eremophilene	
20	Not reported	[36]
21	Ketones; acetoin, 2-tridecanone, (e)-β-damascenone, β-ionone, and γ-nonalactone<br><br>Alcohols; 1-hexanol, 2-ethyl-hexan-1-ol, linalool, 2,3-butanediol, geraniol, benzenemethanol, 2-phenylethanol, and 1-tridecanol<br><br>Acids; acetic acid, butyric acid, hexanoic acid, octanoic acid, and decanoic acid<br><br>Aldehydes; benzaldehyde, phenylacetaldehyde, and pentadecanal<br><br>Esters; ethyl benzoate, methyl salicylate, phenethyl acetate, ethyl tetradecanoate, methyl palmitate, and ethyl palmitate	[37]

	Others; 1-hexadecyne, benzothiazole, and 2-pyrrolidinone<br><br>Volatile phenols; phenol, guaiacol, eugenol, and 4-vinylguaiacol	
22	Acids; acetic acid, butanoic acid, hexanoic acid, benzoic acid, octanoic acid, and nonanoic acid<br><br>Alcohols; ethanol, 1-butanol, 2-butanol, 2-methylpropanol, methionol, 1-pentanol, 3-methylbutanol, 1-hexanol, phenylmethanol, 1-octanol, 1-octen-3-ol, and 2-phenylethanol<br><br>Aldehydes; hexanal, trans, trans-2,4-hexadienal, 2,4-heptadienal, benzaldehyde, trans-2-heptenal, 2-octenal, phenylacetaldehyde, ethylbenzaldehyde, nonanal, trans, trans-2,4-nonadienal, 2,4-decadienal, undecenal, and trans-2-tridecenal<br><br>Ketones; 2-butanone, 3-hydroxy-2-butanone, 2-acetylpyrrole, 3-ethylcyclopentanone, 1-octen-3-one, 2,3-octanedione, 3,5-octadien-2-one, 3-octanone, 3-octen-2-one, and 4-ethylcyclohexanone<br><br>Esters; methyl hexadecanoate, methyl octadecenoate, methyl pyrrole-2-carboxylate, methyl benzoate, methyl octanoate, ethyl 9,12-octadecadienoate, ethyl octadecenoate, ethyl octadecenoate, ethyl acetate, ethyl butanoate, ethyl hexadecanoate, ethyl heptanoate, ethyl octanoate, ethyl phenylacetate, 3-methylbutyl acetate, 2-methylpropyl	[38]

	butanoate, 3-methylbutyl butanoate, hexyl hexanoate, 2-phenylethyl acetate, and 2-phenylethyl 2-methylpropanoate<br><br>Furans; acetylfuran, 2,3-dihydrobenzofuran, 2-pentylfuran, 5-ethyldihydro-2(3h)-furanone, and dihydro-5-pentyl-2(3h)-furanone<br><br>Phenol and phenol derivatives; phenol, 2-methoxyphenol, 2,6-dimethoxyphenol, 4-ethyl-2-methoxyphenol, and 4-vinyl-2-methoxyphenol<br><br>Others; β-caryophyllene, and methylphenyl ether	
23	Esters; ethyl acetate, ethyl propanoate, ethyl 3-methylbutanoate, ethyl crotonate, ethyl isovalerate, ethyl pentanoate, ethyl hexanoate, ethyl 2-methyl-2- butanoate, and ethyl benzoate<br><br>Alcohols; isopentyl alcohol<br><br>Terpenes; limonene<br><br>Others; γ-dodelactone	[39]

	Esters; thioic acid, s-methyl ester, acetic acid, n-propyl ester, ethyl butanoate, ethyl butanoate, ethyl 2-methylbutyrate, isopropyl butanoate, isopropyl butanoate, ethyl 2-methylbutanoate, 3-methyl-2-butenyl acetate, buthyl butanoate, ethyl hexanoate, (e)-3-hexenyl acetate, methyl 3-(methylthio) propanoate, 3-(methylthio) propyl acetate, phenyl methyl acetate, ethyl octanoate, 3-phenylpropyl acetate, and isoamyl octanoate<br><br>Alcohols; 1-hexanol, 1-octen-3-ol, benzyl alcohol, octanol, and (z)-6-nonenol<br><br>Aldehydes; benzaldehyde, octanal, (z)-6-nonenal, (e,z)-2,6-nonadienal, (e)-2-nonenal, decanal, (e)-2-decanal, and hexadecanal<br><br>Ketones; 1-octen-3-one	
24	Alcohols; isoamyl alcohol, 1-octen-3-ol, 2-octanol, (z)-1,5-octadien-3-ol, (e)-2-octenol, and 2-phenylethanol<br><br>Ketones; diacetyl, and 2-acetyl-1-pyrroline<br><br>Acids; acetic acid, butyric acid, and isovaleric acid<br><br>Aldehydes; hexanal, methional, 2-phenylacetaldehyde, (e)-2-octenal, (e)-2-nonenal, (e,e) 2,4-nonadienal, and (e,e)-2,4-decadienal	[40]

	Esters; isoamyl acetate<br><br>Pyrazines; 2,3-diethyl-5-methyl-pyrazine<br><br>Sulfur compounds; methanethiol	
	Ketones; diacetyl, 2-butanone, 2-acetyl-1-pyrroline, 3-octanone, 3,5-octadien-2-one, and 3-carvomenthenon<br><br>Alcohols; isoamyl alcohol, 1-octen-3-ol, (z)-1,5 -octadien-3-ol, (e)-2-octenol, 2-phenylethanol, and (e,z)-2,6-nonadien-1-ol<br><br>Aldehydes; hexanal, methional, (e)-2-octenal, and (e,e)-2,4-nonadienal<br><br>Esters; ethyl butyrate, and hexyl acetate<br><br>Acids; butyric acid, and isovaleric acid<br><br>Pyrazines; 2,3-diethyl-5-methyl-pyrazine	

25	Terpenes; 1-methyl-4-(1-methylethylidene)-cyclohexene, 1-methyl-4-(1-methylethyl)-1,3-cyclohexadiene, (+)-4-carene, o-cymene, 3-carene, d-limonene, γ-terpinene, β-myrcene, α-phellandrene, and β-phellandrene<br><br>Alcohols; ethanol, 2-penten-1-ol, (z)-3-hexen-1-ol, 1-octanol, 3-methyl-1-butanol, phenylethyl alcohol, 3,7-dimethyl-1,6-octadien-3-ol, 1,1-bicyclopentyl-1,1-diol, 3-methyl-3-buten-1-ol, 2-ethyl-1-hexanol, and α-terpineol<br><br>Esters; ethyl acetate, octanoic acid, ethyl ester, 5-methyl-4-thiazoleethanol, acetate, butanoic acid, ethyl ester, cis-3-hexenyllactate, decanoic acid, ethyl ester, hexanoic acid, ethyl ester, dodecanoic acid, ethyl ester, 3-methyl-1-butanol, acetate, acetic acid,2-phenylethyl ester, octanoic acid,3-methylbutyl ester, isopentyl hexanoate, octanoic acid, methyl ester, 4-terpinenyl acetate, and formic acid, hexyl ester<br><br>Acids; butanoic acid, hexanoic acid, acetic acid, octanoic acid, and n-decanoic acid<br><br>Aldehydes; furfural, and nonanal<br><br>Ketones; 3-penten-2-one, 4-hydroxy-2-pentanone, tetrahydro-6-pentyl-2h-pyran-2-one, 5-ethyldihydro-2(3h)-furanone, 5-heptyldihydro-2(3h)-furanone, acetoin, and 2,3-butanedione<br><br>Alkanes; (2-methyl-1-propenyl)-benzene, styrene, 2,6,10,14-tetramethyl-hexadecane, 2,6,10,14-tetramethyl-pentadecane, and toluene	[41]

	Others; methoxy-phenyl-oxime, dimethyl ether, and trans-2-(2-pentenyl) furan	
26	Acids: acetic acid, 3-methylbutanoic acid, 2-hydroxy-2-methylbutyric acid, hexanoic acid, heptanoic acid, octanoic acid, and nonanoic acid<br><br>Alcohols: ethanol, 3-methyl-1-butanol, 1-heptanol, 2-ethyl-1-hexanol, 3,3-dimethyl-cyclohexanol, and benzyl alcohol<br><br>Aldehydes: acetaldehyde, hexanal, heptanal, 3-methyl-2-butenal, octanal, nonanal, and benzaldehyde<br><br>Alkanes: 4-methyloctane, decane, and 2,4-dimethylheptane<br><br>Esters: ethyl 2-methylpropanoate, methyl 3-methylbutanoate, ethyl butyrate, ethyl 2-methylbutanoate, ethyl 3-methylbutanoate, 3-methyl-1-butyl acetate, ethyl pentanoate, propyl 3-methylbutanoate, 2-methylpropyl butanoate, isobutyl 2-methylbutanoate, methyl hexanoate, isobutyl 3-methylbutanoate, 3-methylbutyl 2-methylpropanoate, ethyl 3-methyl-2-butenoate, ethyl hexanoate, 3-methylbutyl butanoate, 3-methylbutyl 2-methylbutanoate, 2-methylbutyl 2-methylbutanoate, 3-methylbutyl 3-methylbutanoate, propyl hexanoate, ethyl heptanoate, pentyl 3-methylbutanoate, 2-methylpropyl hexanoate, 3-methylbutyl pentanoate, 3-methyl-3-buten-1-yl 3-methylbutanoate, 4-methylpentyl 3-methylbutanoate, ethyl 3-hydroxy-3-methylbutanoate, 3-methylbut-2-enyl pentanoate, ethyl 2-hydroxy-3-methylbutanoate, ethyl octanoate, hexyl 3-methylbutanoate, 3-methylbutyl hexanoate, 3-methybutyl 3-methylbut-2-enoate, ethyl 4-octenoate, pentyl hexanoate, propyl octanoate, pent-4-enyl hexanoate, 2-methylpropyl	[42]

	octanoate, 3-methylbutyl heptanoate, methyl benzoate, ethyl decanoate, 3-methylbutyl octanoate, ethyl trans-4-decenoate, ethyl benzoate, 2-furylmethyl 3-methylbutanoate, methyl salicylate, ethyl phenylethanoate, ethyl dodecanoate, phenylmethyl 3-methylbutanoate, 3-methylbutyl benzoate, 2-phenylethyl pentanoate, 3-methylbut-3-enyl benzoate, and phenylacetic acid isoamyl ester<br><br>Ketones: acetone, 2-butanone, 2-pentanone, 2,3-butanedione, 2-heptanone, acetoin, 6-methyl-5-hepten-2-one, 2-nonanone, and 2-undecanone<br><br>Terpenes: β-ocimene, prenol, (z)-3-hexen-1-ol, and linalool<br><br>Sulphurs: methanethiol, dimethyl disulfide, and methyl propyl sulfide	
27	Alcohols: cis-2-hexen-1-ol, 2-ethylhexanol, trans-2-hexene-1-ol, n-hexanol, trans-3-hexen-1-ol, 2-nonanol, 1-octanol, 1-nonanol, phenylethyl alcohol, 1-dodecanol, 6-methyl-1-heptanol, isoamyl alcohol, linalool, cis-verbenol, terpinen-4-ol, l-menthol, alpha-terpineol, 2,2,6-trimethyl-6-vinyltetrahydro-2 h-pyran-3-ol, (r)-(+)-β-citronellol, citronellol, nerol, geraniol, lavandulol, isopulegol, (-)-terpinen-4-ol and (s)-(-)-alpha-terpineol<br><br>Esters: 1-methyl-4-(1-methylvinyl) cyclohexyl acetate, ethyl phenylacetate, isoamyl laurate, linalyl butyrate and geranyl isovalerate<br><br>Alkenes: myrcene	[43]

	Aldehydes: p-tolualdehyde, citronellal, 4-phenylbutanal, (e)-3,7-dimethyl-2,6-octadienal, 3-methylbenzaldehyde, and citral<br><br>Ketones: 2-octanone, damascenone, and geranylacetone<br><br>Phenols: isoeugenol, and 2,4-di-tert-butylphenol<br><br>Acids: 1-hexanoic acid, geranic acid, cis-8,11,14-eicosatrienoic acid, octanoic acid, nonanoic acid, acetic acid, 2-methyl-2-pentenoic acid, and 3-hydroxydodecanoic acid	
28	Aldehydes: 3-methyl-butanal, octanal, decanal, dodecanal, safranal, perillaldehyde, hexanal, heptanal, citral, benzaldehyde, lilac aldehyde, (e)-2-nonenal<br><br>Alcohols: ethanol, 3-methyl-1-butanol, 3-buten-2-ol, 2-methyl-, 2-methyl-3-buten-2-ol, 1-hexanol, p-mentha-1,8-dien-7-ol, 1-heptanol, perillic alcohol, 2-ethyl-1-hexanol, 1-nonanol, p-menth-1-en-9-ol, benzyl alcohol, 1-octanol, 1-decanol, 1- dodecanol, 2-menthoxy-4-vinyphenol, 1,5,7-octatrien-3-ol,2,6-dimethyl, and spathulenol<br><br>Esters: hexanoic acid, 3-hydroxy-, ethyl ester, ethyl 3-hydroxyhexanoate, citronellyl formate, methyl octanoate, isopropyl myristate, hexadecanoic acid, methyl ester, and methyl benzoate	[44]

	Ketones: acetone, acetoin, diacetyl, herbal ketone, isopiperitenone, 2-cyclopentyl cyclopentanone, p-methylacetophenone, ethenone,1-(2-hydroxy-5-methylphenyl)-, p-menth-8-en-2-one, and sulcatone<br><br>Terpenes: 1r-α-pinene, β-pinene, limonene, dihydrocarvone, valencene, β-sinensal, ɤ-terpinene, α -terpineol, 1,3,8-p-menthatriene, prenol, β-cyclocitral, cis)-rose oxide, β-linalool, citronellol, nerol, geraniol, cis-geraniol, trans-geraniol, geranyl acetone, trans-nerolidol, nerolidol, ɤ-eudesmol, β-isophorone, α-copaene, caryophyllene oxide, (-)-4-terpineol, 4-terpineol, (+)-trans-carveol, (+)-cis-carveol, (-)-cis-carveol, (-)-trans-carveol, carveol, eugenol, menthol, cis-p-mentha-2,8-dien-1-ol, eucarvone, carvone, and piperitenone<br><br>Furans: furfural, 5-methylfurfural, and 2-acetylfuran<br><br>Suphides: dimethyl sulfide<br><br>Others: 2-p-tolylpropene, and butylated hydroxytoluene	
	Aldehydes: acetaldehyde, butanal, 3-methyl, octanal, nonanal, decanal, undecanal, hexanal, heptanal, 2-butenal, 2-methyl, 2-hexenal, propanal,3-(methythio), 2,6-nonadienal, e, z, 2-phenyl-2-butenal, 2-pentenal, 2-methyl, benzaldehyde, (e)-2-octenal, (e)-2-nonenal, (e)-2-decenal, and 1-cyclohexene-1-acetaldehyde, 2,6,6-trimethyl<br><br>Esters: ethyl acetate, methyl 2- (methylthio)acetate, hexanoic acid, hexyl ester, benzeneacetic acid, 2-hexenyl ester, ethyl trans-4-decenoate, butanoic acid, ethyl ester, butanoic acid,2-methyl-, ethyl ester, butanoic acid, 2-methyl-	

	ethyl ester, butanoic acid, methyl ester, butanoic acid, 3-hydroxy-, ethyl ester, butanoic acid, 2-methyl-2-methylpropyl ester, hexanoic acid, ethyl ester, acetic acid, hexyl ester, butanoic acid, 2-methyl-2-methylbutyl ester, 3-hexenoic acid, ethyl ester, hexanoic acid, butyl ester, isopentyl hexanoate, 2.4-hexadienoic acid, ethyl ester, acetic acid, octyl ester, octanoic acid, ethyl ester, decanoic acid, ethyl ester, dodecanoic acid, ethyl ester, ethyl laurate, isopropyl myristate, and hexadecanoic acid, ethyl ester<br><br>Alcohols: 1-propanol, 2-methyl, ethanol, 1-hexanol, 3-hexen-1-ol, (z), 1-heptanol, 5-octen-1-ol, 3,6-nonadien-1-ol, 2-ethyl-1-hexanol, 6-nonenol, 1-nonanol, benzyl alcohol, phenylethyl alcohol, 1-octanol, 1-decanol, 5-decen-1-ol, 1-dodecanol, 2-methoxy-4-vinylphenol, 1-butanol, 2-methyl, 3-phenylpropanol, 1-butanol, 1-pentanol, and 1-octen-3-ol<br><br>Sulphides: disulfide dimethyl, and dimethyl trisulfide<br><br>Ketones: acetoin, 5-hepten-2-one,6-methyl, cyclohexanone,2,2,6-trimethyl, 5,9-undecadien-1-one, 6,10-dimethyl, 2-butanone, 4-(2,6,6-trimethyl-1-cyclohexen-1-yl), and ethanone, 1-(2-pyridinyl)<br><br>Terpenes: eucalyptol, d-limonene, α-terpineol, β-linalool, geraniol, β-damascenone, α-ionone, trans-β-ionone, α-cadinene, β-cadinene, and eugenol<br><br>Alkene: cadina-3,9-diene<br><br>Furans: furfural	

29	Alkenes: alpha-terpinene<br><br>Alcohols: benzyl alcohol, 2-hexanol, 2,3-butanediol, 1-octen-3-ol, and 3-methylbutan-1-ol<br><br>Aldehydes: nonanal-(M), hexanal-(M), hexanal-(D), 3-methylbutanal, (E)-2-heptenal, phenylacetaldehyde, heptanal, butanal, and benzaldehyde<br><br>Esters: ethyl hexanoate-(M), ethyl hexanoate-(D), methyl hexanoate, propyl butanoate, ethyl acetate, isoamyl acetate, butyl propanoate, butyl acetate, propyl acetate, ethyl 2-hydroxypropanoate, and ethyl acrylate<br><br>Ketones: 2-butanone, 2-propanone, 2-heptanone, 3-hydroxy-2-butanone, acetophenone, and 6-methyl-5-hepten-2-one<br><br>Acids: acetic acid<br><br>Furans: 2-pentylfuran<br><br>Others: 5-methyl-2-formyl-thiophene, 2,4,5-trimethyl-thiazle, and 1,4-dioxane	[45]

30	Aldehydes: pentanal, hexanal, heptanal, octanal, 2-heptenal, nonanal, 2-octenal (E), furfural, decanal, benzaldehyde, 4-ethyl, benzaldehyde, 2,5-dimethyl, and 2,4-decadienal<br><br>Alcohols: propan-2-ol, ethanol, 1-butanol,3-methyl, 1-pentanol, 1-hexanol, 3-octanol, 1-octen-3-ol, 1-heptanol, 4-ethylcyclohexanol, 1-octanol, 2-octen-1-ol, benzyl alcohol, and phenylethyl alcohol<br><br>Ketones: acetone, 2-butanone, 2-heptanone, 2-octanone, 2-butanone-3-hydroxy, 2-nonanone, 3-octen-2-one, 3,5-octadien-2-one, and 2-undecanone<br><br>Furans: furan, 2-ethyl, 2-n-butyl furan, furan, 2-pentyl, and furan, 2-(1-pentenyl)-(E)<br><br>Other: heptane, and octane	[46]
31	Acids: acetic acid, propanoic acid, crotonic acid, 2-methyl-propanoic acid, butanoic acid, 2-methyl-butanoic acid, pentanoic acid, hexanoic acid, heptanoic acid, octanoic acid, nonanoic acid, (e)-2-octenoic acid, n-decanoic acid, benzoic acid, and dodecanoic acid<br><br>Aldehydes: hexanal, (e)-2-hexenal, (e)-2-heptenal, (e)-2-octenal, furfural, (e)-2-nonenal, benzaldehyde, 5-methyl-2-furancarboxaldehyde, and benzeneacetaldehyde<br><br>Alcohols: 1-hexanol, (e)-2-hexen-1-ol, 1-octen-3-ol, 2-ethyl-1-hexanol, 2-furanmethanol, benzyl alcohol, and phenylethyl alcohol	[47]

	Esters: hexanoic acid methyl ester, benzoic acid methyl ester, 5-ethyldihydro-2(3h)-furanone, dodecanoic acid methyl ester, and 5-butyldihydro-2(3h)-furanone<br><br>Ketones: 3-heptanone, 2-heptanone, cyclohexanone, 2-octanone, 6-methyl-5-hepten-2-one, and trans-damascenone<br><br>Others: ethylbenzene, limonene, 2-methyl pyrrolidine, 2-methylpyrazine, 2-ethyl-5-methyl-pyrazine, butylated hydroxytoluene, and 1-(1h-pyrrol-2-yl)-ethanone	
32	Acids: nonanoic acid<br><br>Alcohols: ethanol, 2-methyl-1-butanol, 1-pentanol, 1-hexanol, 3-hexen-1-ol, 1-octen-3-ol, 1-heptanol, 6-methyl-5-hepten-2-ol, 2-hepten-1-ol, 1-octanol, 2-octen-1-ol, 1-nonanol, 3-nonen-1-ol, 2-nonen-1-ol, and 2,6-nonadien-1-ol<br><br>Aldehydes: acetaldehyde, butanal, pentanal, hexanal, heptanal, 2-hexenal, octanal, 2-heptenal, nonanal, e-2-octenal, 2-nonenal, 2,6-nonadienal, 2-decenal, and 2,4-decadienal<br><br>Alkanes: decane, and 2,6-dimethyl-2,6-octadiene<br><br>Esters: 1-hexyl acetate<br><br>Furans: 2-methylfuran, 2-ethylfuran, 2-pentylfuran, and 2-butyltetrahydrofuran	[48]

	Ketones: 2-propanone, 2,3-butanedione, 3-octanone, 2-octanone, 6-methyl-5-hepten-2-one, and 4-decanone<br><br>Sulphur compounds: dimethyl disulfide<br><br>Terpenes: α-terpinene, perillene, geraniol, geranyl acetone, eucalyptol, β-cyclocitral, and β-ionone<br><br>Others: chloroform, and unknown (2)	
33	Alcohols: ethanol, 1-penten-3-ol, isoprenol, isoamyl alcohol, 1-pentanol, prenyl alcohol, 2,3-butanediol, (e)-3-hexen-1-ol, (z)-3, hexen-1-ol, 4-hexen-1-ol, (e)-2-hexen-1-ol, hexanol, 1-heptanol, 6-methyl-5-hepten-2-ol, 2-ethyl-1-hexanol, trans-2-octen-1-ol, 1-octanol, 2-nonanol, 1-nonanol, and 1-undecanol<br><br>Aldehydes: acetaldehyde, valeraldehyde, hexanal, (e)-2-hexenal, heptanal, benzaldehyde, octanal, (e)-2-octenal Exo-isocitral, nonanal, 4-ethylbenzaldehyde, decanal, and (e)-2-decenal<br><br>Esters: ethyl lactate, ethyl butyrate, ethyl hexanoate, hexyl acetate, 2-hexenyl acetate, and ethyl benzoate<br><br>Ketones: acetone, 2,3-butanedione, acetoin, 2-heptanone, 1-octen-3-one, methyl heptanone, 2-octanone, acetophenone, 2, nonanone, piperitone, and 2-undecanone<br><br>Terpenes: (z)-β-ocimene, terpinen-4-ol, α-terpineol, cineole, (-)-α-cubebene, and δ-cadinene	[49]

34	Acids: acetic acid, 2-methyl butyric acid, and heptanoic acid<br><br>Alcohols: ethanol, 2-methyl-2-propanol, 3-methyl-3-buten-1-ol, 3-methyl-1-butanol, 2-methyl-1-butanol, 1-pentanol, 3-methyl-2-buten-1-ol, 2,3-butanediol, leaf alcohol, 1-hexanol, 1-heptanol, 1-octen-3-ol, 6-methyl-5-hepten-2-ol, (e)-oct-2-en-1-ol, 1-octanol, 2-nonanol, phenylethyl alcohol, 4-ethyl phenol, 1-nonanol, and (e)-5-decen-1-ol<br><br>Aldehydes: acetaldehyde, 2-methyl propanal, 3-methyl butyraldehyde, 2-methyl butyraldehyde, benzaldehyde, phenylacetaldehyde, decanal, and β-cyclocitral<br><br>Esters: 2-propenyl propanoate, ethyl acetate, ethyl propionate, propyl acetate, ethyl lactate, 2-methyl butyl butyrate, hexyl formate, isoamyl acetate, methyl 2-hydroxy-4-methylvalerate, ethyl hexanoate, hexyl acetate, ethyl octanoate, ethyl nonanoate, ethyl caprate, ethyl myristate, isopropyl myristate, ethyl palmitate, and ethyl pentadecanoate<br><br>Ketones: acetone, diacetyl, 2-pentanone, acetoin, 2-heptanone, 6-methyl-2-heptanone, 6-methyl-5-hepten-2-one, 2-octanone, 2,2,6-trimethylcyclohexanone, 2-nonanone, and 2-tridecanone<br><br>Terpenes: terpinolene, d-limonene, and g-terpinene<br><br>Others: theaspirane	[50]

35	Acids: isobutyric acid, isovaleric acid, dl-2-methylbutyric acid, hexanoic acid, acetic acid glacial, decanoic acid, and ethyl caprylic acid<br><br>Alcohols: ethanol, 1-pentanol, 1-butanol, hexyl alcohol, 3-methyl-1-butanol, 2-methyl-1-butanol, (r, r)-2,3-butanediol, (r)-(-)-1,3-butanediol, phenethyl alcohol, 1-octanol, and n-heptanol<br><br>Aldehydes: acetaldehyde, decanal, benzaldehyde, 4-ethylbenzaldehyde, and isovaleraldehyde<br><br>Esters: ethyl acetate, ethyl butyrate, butyl acetate, 2-methylbutyl acetate, banana oil, hexyl acetate, ethyl caprylate, ethyl caprate, isoamyl acetate, ethyl caproate, phenethyl acetate, methyl n-caprate, and ethyl laurate<br><br>Ethers: ethyl vinyl ether<br><br>Ketones: 2-heptanone, 2-octanone, 3-hydroxy-2-butanone, acetophenone, 4-ethylacetophenone, 3-nonanone, acetone, 2, nonanone, 2-tridecanone, and 2-undecanone<br><br>Olefins: dodecane, tetradecane, and eicosane<br><br>Others: 2,4-di-tert-butylphenol	[51]
